# Light-controlled inhibition of Gram-positive bacteria by photoswitchable amphiphilic lipids (PALs)

**DOI:** 10.1039/d6md00096g

**Published:** 2026-05-01

**Authors:** Guilherme F. S. Fernandes, Seong-Heun Kim, Charlotte K. Hind, Jessica Furner-Pardoe, Janis Romanopulos, Christian D. Lorenz, A. James Mason, J. Mark Sutton, Daniele Castagnolo

**Affiliations:** a Department of Chemistry, University College London 20 Gordon Street London WC1H 0AJ UK d.castagnolo@ucl.ac.uk; b Institute of Pharmaceutical Science, School of Cancer & Pharmaceutical Science, King's College London 150 Stamford Street SE1 9NH London UK; c Antimicrobial Discovery, Development and Diagnostics, Vaccine Development and Evaluation Centre, UKHSA Porton Down Salisbury SP4 0JG UK; d Biological Physics and Soft Matter Group, Department of Physics, King's College London London WC2R 2LS UK

## Abstract

Amphiphilic lipids are potent membrane-disrupting antibacterials, but their activity cannot normally be modulated after administration. Here we report photoswitchable amphiphilic lipids (PALs) that enable reversible, light-controlled modulation of antibacterial potency through photoinduced changes in molecular conformation. The PALs incorporate azobenzene photoswitches linking quaternary ammonium headgroups to variable alkyl chains, allowing their geometry and membrane affinity to be tuned by *trans*–*cis* isomerisation. Systematic studies against multidrug-resistant Gram-positive bacteria revealed a striking, chain-length-dependent photomodulation. Short-chain PALs lost activity upon irradiation, whereas long-chain derivatives became strongly antibacterial in their *cis*-enriched states, with up to 32-fold reductions in MICs. Molecular dynamics simulations correlated these changes with light-dependent differences in membrane insertion depth and molecular orientation, providing a structural basis for optical control of membrane disruption. These findings establish a mechanistic framework for designing photoswitchable amphiphiles that translate molecular photoisomerisation into controllable biological function, extending the reach of photopharmacology into antibacterial membrane-active agents.

## Abstract 2

Photoswitchable amphiphilic lipids (PALs) enable reversible light-controlled inhibition of Gram-positive bacteria. Azobenzene photoisomerization modulates membrane insertion, switching antibacterial activity on or off depending on alkyl chain length.

## Graphical abstract

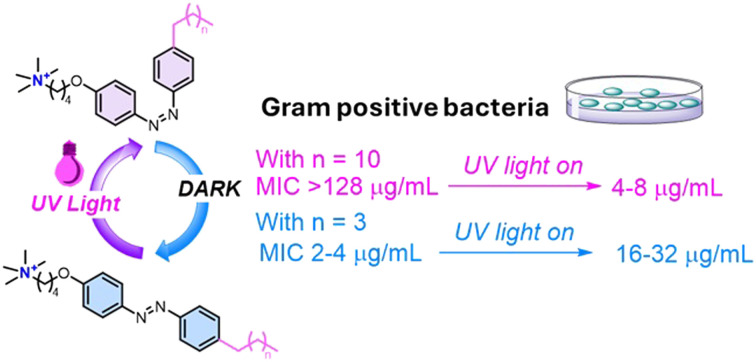

## Introduction

**Fig. 1:**
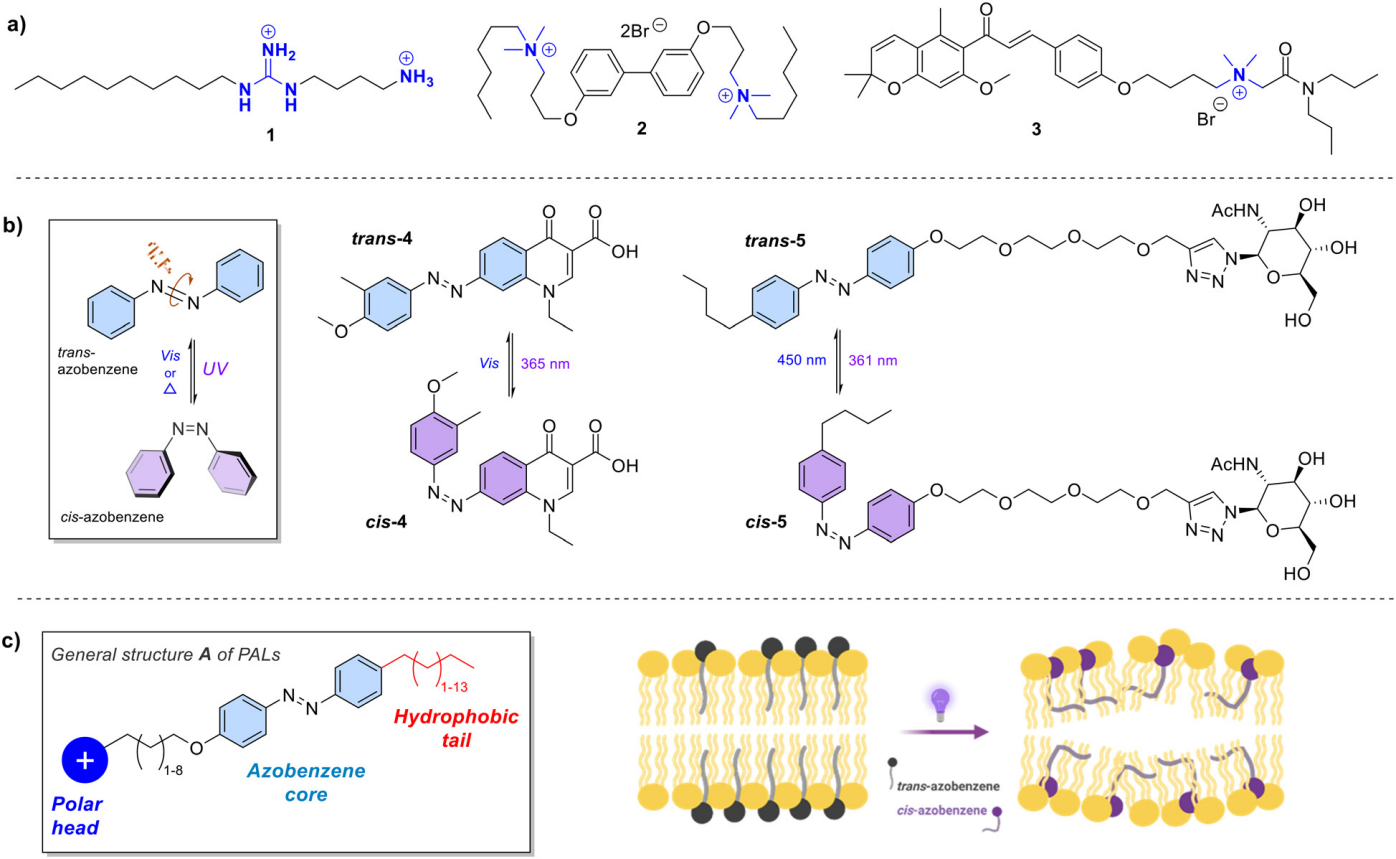
a) Cationic amphiphile antibacterials; b) azobenzenes with antibacterial activity; c) general structure of photoswitchable amphiphilic lipids (PALs) and their putative interaction with bacterial membranes. Created with https://www.BioRender.com.

Cell membranes are essential for bacterial viability, serving as dynamic platforms for metabolism, nutrient transport, and homeostatic regulation.^1–3^ Owing to their central role, they have emerged as compelling targets for the development of next-generation antibacterial agents.^4,5^ Among the most promising classes of membrane-damaging molecules are cationic amphiphiles, which exhibit potent antibacterial activity through membrane disruption.^6–8^ Although their precise mechanisms of action remain only partially elucidated, it is widely accepted that these molecules penetrate the bacterial cell wall and interact with membrane lipids and proteins, leading to structural disorganization, leakage of cytoplasmic contents, and degradation of intracellular components.^1,4,5,9,10^ Structurally, cationic amphiphiles typically contain at least one positively charged headgroup, such as a quaternary ammonium cation (QAC) or a guanidinium moiety, that electrostatically associates with the negatively charged phospholipids of bacterial membranes (Fig. 1a). Concurrently, their hydrophobic structural components intercalate into the membrane core, increasing surface pressure and inducing a transition toward a liquid-crystalline state. This process perturbs membrane integrity, alters osmoregulatory balance, and reduces the hydrophobic barrier of the lipid bilayer, ultimately compromising cell viability.^10–12^

Photopharmacology has recently emerged as a strategy to modulate drug conformation, activity, and selectivity using light-responsive photoswitches.^13–15^ Azobenzenes are particularly attractive photoswitches due to their efficient and reversible *trans*–*cis* isomerization upon light irradiation.^16–18^ Azobenzene-containing compounds have been applied in antitumor therapies,^19–21^ ion channels,^22,23^ HDACs,^24,25^ kinases,^26,27^ and antibacterials.^18,28^ Despite notable examples, such as the azobenzene–quinolone conjugate 4 that displays enhanced antibacterial activity against *E. coli*^29^ and the carbohydrate–azobenzene surfactant 5 capable of controlling biofilm growth (Fig. 1b),^30^ the development of photoswitchable antibacterials still remains limited. However, azobenzene-based photoswitchable antimicrobials still hold significant potential for localized therapeutic applications. Since the photoisomerisation of azobenzene derivatives is typically triggered by UV or blue light, which have limited tissue penetration, these systems are particularly well suited for topical or surface-associated treatments.

Potential scenarios include light-directed treatment of superficial infections such as wounds, as well as catheter- or implant-associated infections, where controlled illumination can be applied at the infection site. In addition, PALs could be envisioned as photoresponsive coatings for medical surfaces, enabling spatial and temporal control of antibacterial activity.

Building on our broader interest in membrane-interacting antibacterial agents such as lipoguanidines and in bacterial membranes as dynamic therapeutic targets,^6^ here we explore how light can be harnessed to modulate the interactions between bacterial membrane and lipid-based antibacterials in a controllable manner.

We designed and synthesized a new class of photoswitchable amphiphilic lipids (PALs) that combine the membrane-interacting features of amphiphilic molecules with the reversible conformational control of molecular photoswitches (general structure A, Fig. 1c). These PALs incorporate one or two quaternary ammonium (QAC) headgroups connected through an azobenzene unit to hydrophobic *n*-alkyl tails of variable lengths. We hypothesize that light-induced *trans*–*cis* isomerization of the azobenzene core will alter the molecular conformation and packing of the PALs at the membrane interface, thereby modulating their ability to insert into and potentially disrupt bacterial membranes. This approach provides a mechanistic framework for developing light-responsive, membrane-targeting antibacterials whose activity can be precisely tuned through external optical stimuli. Here we demonstrate that light-driven conformational control can switch antibacterial activity on or off depending on the amphiphile chain length, establishing a direct link between molecular geometry and membrane disruption efficiency.

## Results and discussion

**Scheme 1:**
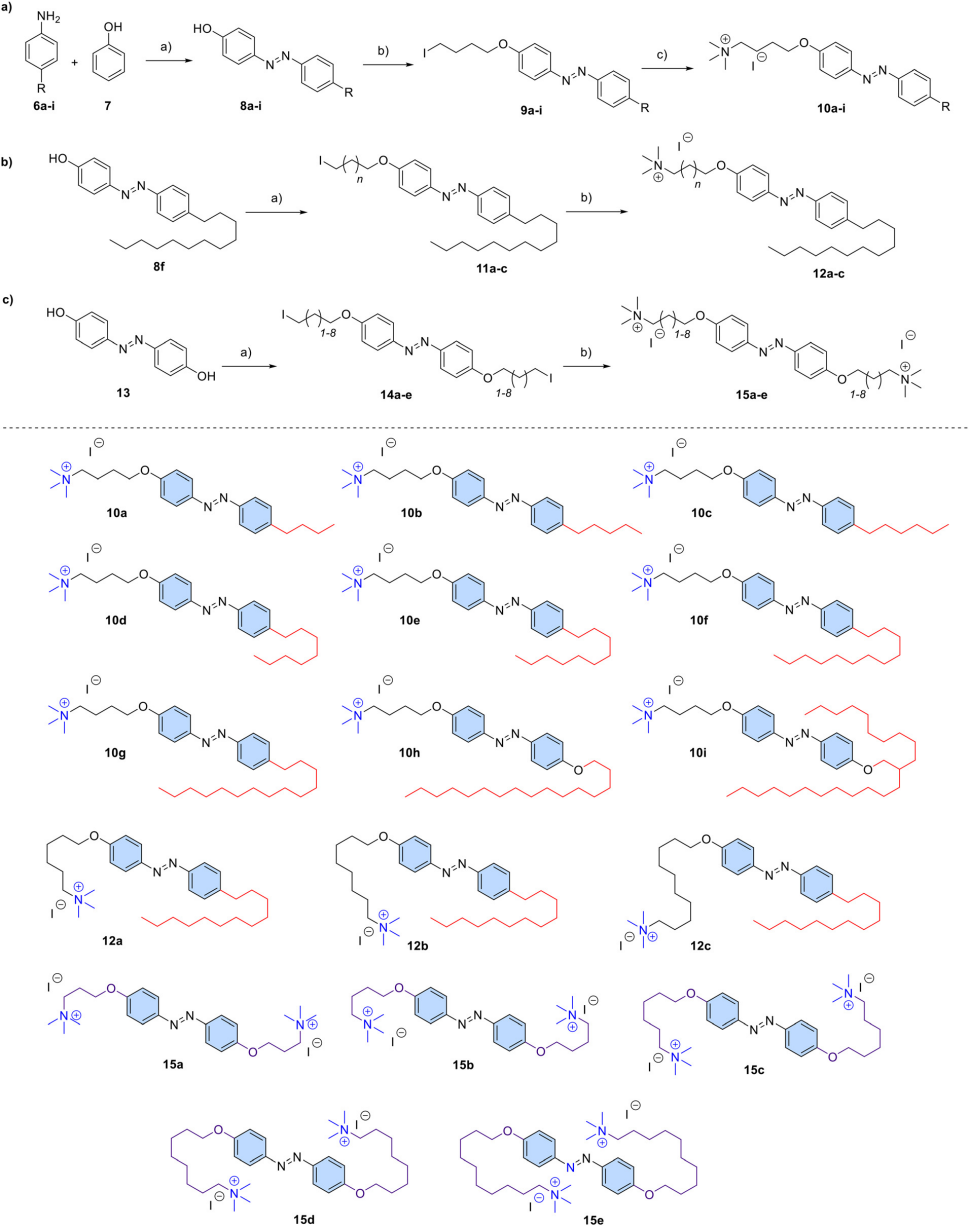
First series of photoswitchable amphiphilic lipids. Conditions: a) NaNO_2_, NaOH, Na_2_CO_3_, HCl, H_2_O, acetone, 0 °C > RT, 3 h, 22–69%; b) 1,4-diiodobutane, Cs_2_CO_3_, acetone, 80 °C, 1 h, 46–83%; c) trimethylamine, THF, 75 °C, 1 h, 90–99%.

A library of PALs was designed and synthesized as reported in Scheme 1. PALs 10a–i, presenting different hydrophobic alkyl chains while keeping the cationic ammonium group at four carbon unit distance from the azobenzene switch, were first synthesized. The azobenzene motifs 8a–i were obtained through an azo coupling reaction between appropriate anilines 6a–i and phenol 7. Subsequently, the derivatives 8a–i underwent alkylation with 1,4-diiodobutane *via* an SN_2_ reaction, resulting in alkylated intermediates 9a–i. Finally, the quaternary ammonium cation was inserted through nucleophilic substitution of iodines with trimethylamine, leading to the formation of the positively charged PALs 10a–i.

A second series of derivatives 12a–c having the same aliphatic hydrophobic chain as 10f and the polar ammonium cation at different distances from the azobenzene switch, was then synthesized (Scheme 1b). The intermediate 8f was alkylated with appropriate diiodoalkanes to provide the iodo-derivatives 11a–c, which were in turn converted into the PALs 12a–c through reaction with Me_3_N.

Finally, a series of PAL derivatives presenting two QAC moieties (Scheme 1c) was prepared. Five bis-QAC-azobenzenes 15a–e were designed, featuring diverse carbon chain lengths connecting the azobenzene core and the polar QAC heads.

The phenol derivative 13 was first reacted with 2 equivalents of the appropriate diiodoalkanes to give the diiodo-derivatives 14a–e. The latter were finally converted into the bis-QAC-azobenzenes 15a–e through reaction with Me_3_N.

**Fig. 2:**
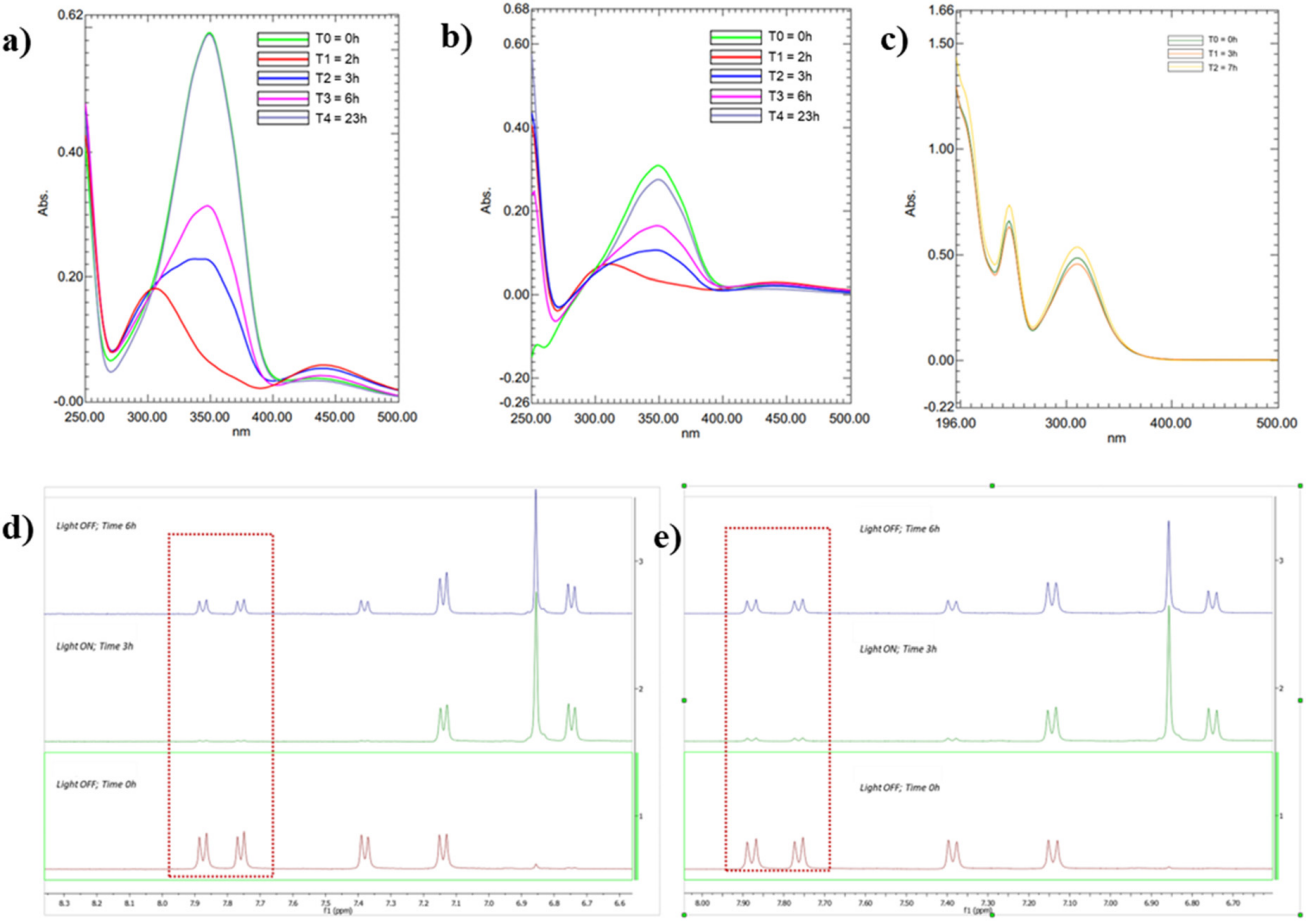
UV-vis spectrophotometric analysis of a) compound 10b and b) compound 10f, showing *trans*-to-*cis* isomerization and *cis*-to-*trans* photorelaxation; c) UV-vis spectrophotometric analysis of the bi-cationic PAL 15c showing no *trans*-to-*cis* isomerization upon 365 nm UV light irradiation; NMR analysis of the photoisomerization of d) 10b and e) 10f, showing the shifting of the aromatic signals due to *trans*-to-*cis* isomerization upon 365 nm UV light irradiation and *cis*-to-*trans* photorelaxation in the dark.

The photoisomerization properties of the PAL compounds were then characterized by UV-vis spectroscopy. Representative spectra for compounds 10b and 10f, which were later found to exhibit opposite trends in the microbiological assays, as well as for the bi-cationic compound 15c are shown in Fig. 2. UV-vis spectroscopic data for other compounds are available in the SI (Fig. S8–S13). PALs 10b and 10f were first irradiated in CH_3_CN with 365 nm UV light for 1.5 hours at 37 °C, to replicate the antibacterial assay conditions. Following UV-vis irradiation, the spectra exhibited a characteristic π → π* transition band for the *trans*-isomers with maximum intensity at *ca.* 350 nm and a characteristic n → π* transition band for their *cis*-isomers with a maximum at *ca.* 440 nm. After UV irradiation, 10b and 10f were left for 24 hours in darkness at 37 °C resulting in the photorelaxation of *cis*-isomers back to *trans*-isomers (Fig. 2a and b). The photorelaxation initiates approximately 4 hours after the UV irradiation was stopped, culminating in the peak *trans*-form after 24 hours. The *trans*-to-*cis* isomerization and the *cis*-to-*trans* photorelaxation of 10b and 10f were also confirmed by ^1^H NMR analysis in DMSO-*d*_6_ solution (Fig. 2d and e). Notably, under dark conditions, PALs exist as thermally stable *trans*-isomers (*trans*-dominated photostationary state (PSS): 89–96%) when freshly prepared. Following irradiation with 365 nm UV light in dark-adapted conditions at 37 °C for 1 hour, the *trans*-isomers efficiently transitioned into *cis*-isomers (*cis*-dominated photostationary state (PSS): 89–94%). Then, when the light was switched off and the PALs were left 6 hours in dark-adapted conditions at 37 °C, the ratio between *trans*- and *cis*-PSS of PALs adjusted at 60% and 40% respectively after 6 hours.

Furthermore, photoisomerization studies in TSB bacterial growth media were carried out to examine the azobenzenes' photoisomerization properties in the media used in antibacterial assays (Fig. S6 and S7). Remarkably, the UV-vis spectra in TSB media displayed analogous transition band characteristics to those observed in CH_3_CN solution. A similar photoisomerization and photorelaxation behaviour was observed for all PALs 10 and 12. Interestingly, no *trans*-to-*cis* isomerization was observed for the bi-cationic compounds 15, as shown in Fig. 2c for 15c when irradiated with 365 nm UV light.

The antibacterial efficacy of PALs 10a–i, 12a–c and 15a–e against a panel of drug-sensitive and MDR Gram-positive bacterial strains (*S. aureus* MSSA ATCC 9144, MRSA NCTC 13616, MRSA USA 300, and MRSA SA 1199B; *E. faecalis* VSE NCTC 775 and VRE NCTC 12201; *E. faecium* VRE NCTC 12204) was then assessed through an OD_600_ bacterial growth assay (see SI for experimental details).^6^ Two parallel sets of experiments were carried out. First, bacteria were incubated with each PAL in their native relaxed *trans*-PSS under dark conditions at 37 °C (Table 1, light OFF (LOFF) experiments). Secondly, to induce photoisomerization to *cis*-enriched PSS, microplates containing bacteria and PALs were exposed to 365 nm UV light for 1 hour under incubation conditions. After this light-on period, the plates were kept in the dark for a further 24-hour incubation at 37 °C (Table 1, light ON (LON) experiments). Importantly, a control experiment involving only bacteria and UV irradiation was carried out, demonstrating the non-toxic nature of the 365 nm UV light on bacteria. The minimum inhibitory concentration (MIC) was employed to evaluate antibacterial activity (Table 1).

**Table 1:** Light control of antibacterial activity in photoswitchable amphiphilic lipids (PALs) against Gram-positive bacteria^a^

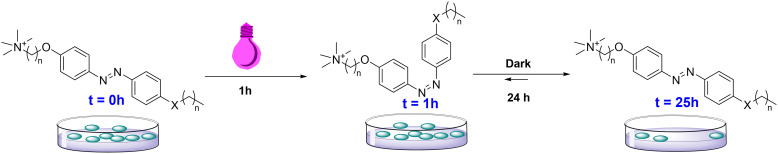															
Entry	Cmpd	MIC (μg mL^−1^)													
		*S. aureus*								*E. faecalis*				*E. faecium*	
		MSSA ATCC 9144		MRSA NCTC 13616		MRSA USA 300		MRSA SA 1199B		VSE NCTC 775		VRE NCTC 12201		VRE NCTC 12204	
		LOFF	LON	LOFF	LON	LOFF	LON	LOFF	LON	LOFF	LON	LOFF	LON	LOFF	LON
*1*	10a	2	**8**	4	**16**	2	**16**	4	**16**	2	**16**	8	16	8	16
*2*	10b	2	**8**	4	**16**	2	**16**	4	**32**	4	**16**	4	**32**	2	**32**
*3*	10c	4	4	4	8	2	8	4	**16**	2	8	4	**16**	2	**16**
*4*	10d	4	4	8	4	4	4	4	4	4	4	4	4	4	4
*5*	10e	16	**2**	16	**2**	16	4	>128	**4**	4	2	16	2	4	2
*6*	10f	>128	**8**	>128	**8**	>128	**4**	>128	**4**	>128	**4**	>128	**4**	>128	**4**
*7*	10g	>128	**16**	>128	**8**	>128	**16**	>128	**8**	>128	**8**	>128	**16**	>128	**16**
*8*	10h	>128	**16**	>128	**2**	>128	>128	>128	>128	>128	>128	>128	>128	>128	>128
*9*	10i	>128	>128	>128	>128	>128	>128	>128	>128	>128	>128	>128	>128	>128	>128
*10*	12a	>128	ND	>128	**4**	>128	**4**	>128	**8**	>128	**4**	>128	**8**	>128	**8**
*11*	12b	>128	**32**	>128	**16**	>128	**16**	>128	**32**	>128	**16**	>128	**32**	>128	**8**
*12*	12c	>128	>128	>128	>128	>128	>128	>128	>128	>128	>128	>128	>128	>128	>128
*13*	15a	>128	>128	>128	>128	>128	>128	>128	>128	>128	>128	>128	>128	>128	>128
*14*	15b	>128	>128	>128	>128	>128	>128	>128	>128	>128	>128	>128	>128	>128	>128
*15*	15c	>128	>128	>128	>128	>128	>128	>128	>128	>128	>128	>128	>128	>128	>128
*16*	15d	128	64	>128	128	128	128	>128	>128	>128	>128	>128	>128	128	>128
*17*	15e	16	8–16	64	16	32	16–32	64	32	32	16–32	64	32–64	64	32

a Color coding: red, ≥3–4-fold increase in MIC after UV irradiation; green, ≥4-fold reduction in MIC.

The majority of PALs demonstrated good micromolar activity against all seven tested Gram-positive bacterial strains, underscoring both potency and broad-spectrum efficacy. Notably, a distinct photomodulation of the antibacterial activity was observed across various compounds. Compounds 10a–c bearing short alkyl chains (4 to 6 carbons) exhibited a substantial loss of potency against all tested strains following UV light irradiation, resulting in an increase in MIC values, from 2–4 μg mL^−1^ to 16–32 μg mL^−1^ (Table 1, *entries 1–3*). The antibacterial activity of PAL 10a, also known as AzoTAB, against *S. aureus* under LOFF conditions is in agreement with that previously described.^30–32^ However, while in previous reports no difference in the antibacterial activity of 10a in both the *cis*-PSS and the *trans*-PSS was observed, we found an increase in the MIC values against all the four strains of *S. aureus* following photoirradiation. Such variation in results can be attributed to different conditions employed for the bacterial assay, such as 5 minutes of irradiation of 10a before incubation with bacteria,^30^*versus* the exposure of 10a to UV light for 1 hour during incubation in our assay. It is plausible that the different exposure time to UV light may determine a different *cis*-PSS/*trans*-PSS ratio or a faster *cis*-to-*trans* relaxation and that the presence of higher amount of *cis*-PSS in our assay may be responsible for the observed increased MICs.

**Fig. 3:**
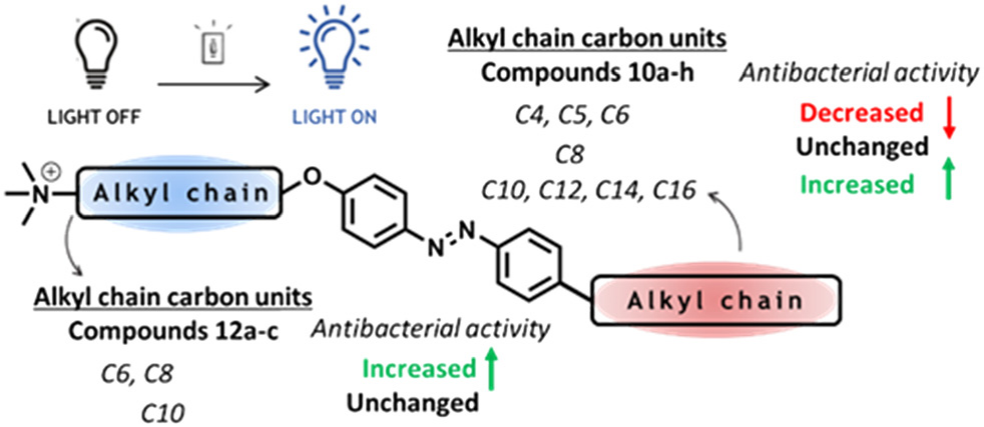
Summary of the structure–activity relationships (SAR) of antimicrobial compounds 10a–h and 12a–c, highlighting the effect of alkyl chain length on minimum inhibitory concentration (MIC) under light-activated conditions. For compounds 10a–c bearing shorter alkyl chains (*C4*–*C6*) an increase of MIC values was observed following UV irradiation, while compounds 10e–h bearing longer chains (*C10*–*C16*) showed an improved activity (decreased MIC) after UV irradiation. Compound 10d with *C8* chain showed no variation in MIC. In contrast, for compound series 12a–c, shorter chains (*C6*–*C8*) are associated with increased potency (reduced MIC), whereas *C10* shows no significant variation.

Compounds 10f–h bearing carbon chains spanning 10–14 carbons, displayed an increased potency against the Gram-positive panel when irradiated with UV-light (Table 1, *entries 6–8*). Compounds 10f and 10g, characterized by 12 and 14 carbons in the alkyl tail, showed no antibacterial activity against all the Gram-positive strains (MIC = ≥128 μg mL^−1^) when in *trans*-PSS respectively, while a remarkable decrease in the MICs (up to 4 μg mL^−1^) was observed upon UV irradiation (MIC reduction >32 fold). Interestingly, PALs 10d and 10e, bearing respectively medium-length 8 and 10 carbon chains, showed good activity against all Gram-positive strains, and no differences in MIC between the *trans*-PSS and the *cis*-PSS (Table 1, *entries 4–5*). Compound 10d was the sole PAL maintaining the same antibacterial activity in both *trans* and *cis* isomeric forms (MIC = 4–8 μg mL^−1^), while compound 10e showed moderate MIC decrease upon UV irradiation on MSSA ATCC 9144 and MRSA NCTC 13616 strains, and a remarkable increase of activity (MIC = 4 μg mL^−1^) on MRSA SA 1199B upon UV light irradiation (*entry 5*). Interestingly, PAL 10h, featuring a 16-carbon alkyl tail, was inactive against all tested Gram-positive strains in both *trans*- and *cis*-PSS, with the exceptions against the MSSA ATCC 9144 and MRSA NCTC 13616 strains, where it exhibited a noteworthy reduction in MIC of 8–64-fold, respectively. Finally, PAL 10i, bearing a double alkyl tail comprising 10 and 12 carbon atoms did not show any activity in either isomeric form, with MIC >128 μg mL^−1^. A clear structure–activity pattern is evident for PALs 10a–i, with shorter chain PALs exhibiting an increase of MIC values upon UV irradiation while a decrease of MICs was observed for PALs with longer chains. PALs 12a–c, bearing different alkyl linkers between the azo-benzene photoswitch and the QAC unit, showed a similar trend confirming the linear correlation between the length of the PAL alkyl chains and the antibacterial activity. As the carbon chain lengthened, PALs 12a–c showed no activity on the *trans*-PSS, while, following photoisomerization upon UV irradiation, they displayed a significant reduction in MICs (4–32 μg mL^−1^, Table 1, *entries 10–11*). On the other hand, PAL 12c, characterized by a 10-carbon long chain, exhibited no antibacterial activity either in the *cis*-PSS or the *trans*-PSS (Table 1, *entry 12*). It is evident that an ideal length of the PAL is required to have an antibacterial activity in the *trans*-PSS (LOFF experiments), Fig. 3.

Previous studies on QAC derivatives clearly showed a correlation between the antibacterial activity and the length of their alkyl tails, with the most effective QAC compounds containing seven to twelve carbon atoms.^33,34^ This behaviour is consistent with the known “cutoff effect” in amphiphilic systems, where activity initially increases with alkyl chain elongation up to an optimal length and then sharply declines due to reduced solubility or membrane partitioning.^35–39^ Interestingly, UV-induced isomerization appears to reverse this inactivity for long-chain PALs, restoring antibacterial efficacy in compounds that are otherwise inactive in their *trans* forms. This finding suggests that photoisomerization can modulate the balance between hydrophobicity and solubility, enabling antibacterial activity even beyond the conventional cutoff length.

Finally, PALs 15a–e, bearing two QACs on each end of the azobenzene core, did not show any antibacterial activity before and after UV light irradiation (Table 1, *entries 13–17*). Since compounds 15a–e are not subject to *trans*-to-*cis* isomerization, and they remain in the *trans*-enriched PSS upon UV irradiation, no differences are observed in the MIC under both LOFF or LON experiments.

All the synthesized PALs were also assayed against a panel of Gram-negative bacteria (*K. pneumoniae* NCTC 13368 and M6; *A. baumannii* AYE and ATCC 17978; *P. aeruginosa* PAO1 and NCTC 13437; and *E. coli* NCTC 12923) under LOFF and LON conditions. In all cases, no antibacterial effect was observed, with MIC values consistently >64 μg mL^−1^ across the studied microorganisms (Table S1). This behaviour can be rationalised by the presence of the LPS-rich outer membrane of Gram-negative bacteria, which limits the penetration of cationic amphiphiles. Short-chain PALs likely lack sufficient hydrophobicity to traverse this barrier, whereas longer-chain derivatives and bis-quaternary ammonium compounds (15a–e), due to their increased hydrophobicity, charge density, and steric bulk, may be preferentially retained at the membrane surface rather than efficiently translocating across it.

**Fig. 4:**
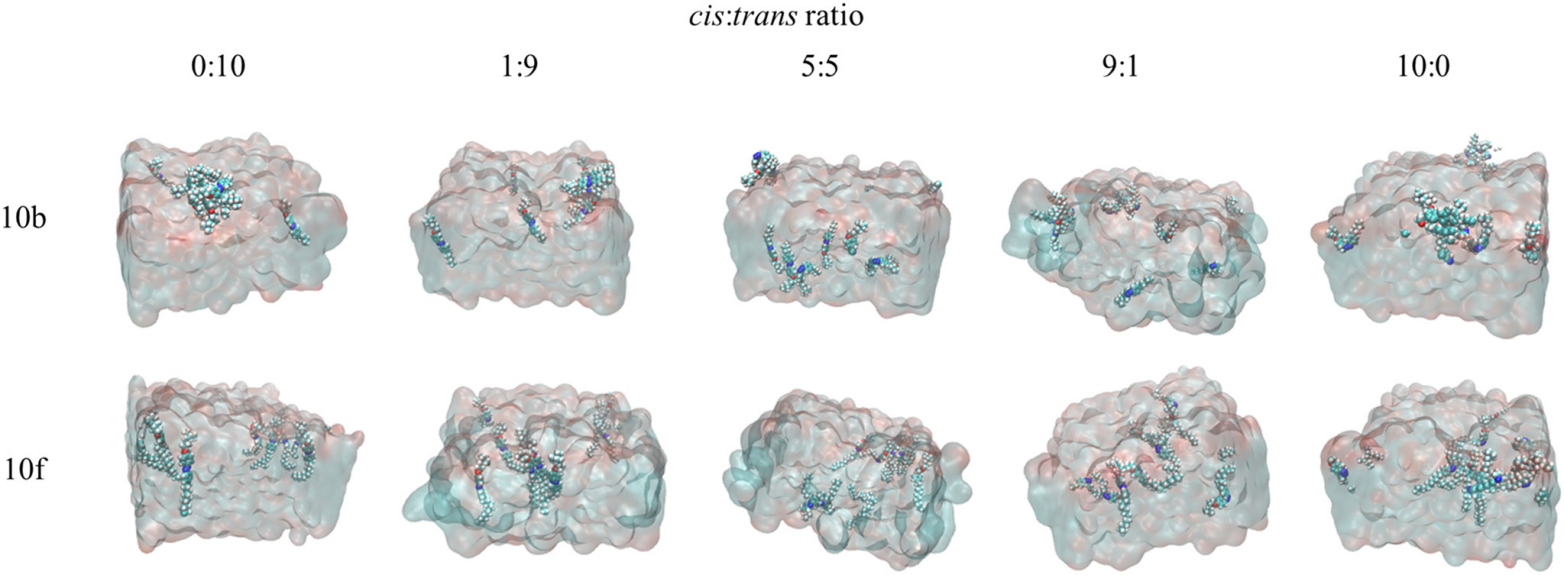
Snapshot of 10 compounds interacting with a model Gram-positive membrane (POPG) during molecular dynamics (MD) simulations. Compounds are shown in vdW representation; membrane is shown in QuickSurf representation. All snapshots were taken at 175 ns, except for compound 10f at a 5 : 5 *cis* : *trans* ratio, which was taken at 140 ns.

The observed reversal of antibacterial activity upon photoisomerization, particularly in long-chain PALs, suggests that molecular conformation may play a crucial role in modulating membrane interactions. To probe this hypothesis, we conducted molecular dynamics (MD) simulations on representative short- and long-chain derivatives, 10b and 10f, to examine how photoinduced isomerisation affects their structural behaviour and interaction with a model Gram-positive membrane (Fig. 4).

Analysis of C–N=N–C dihedral angles and the fraction of these dihedrals in the *cis* conformation across both single- and mixed-conformer systems revealed that both compounds largely retained their initial conformations throughout the simulation. In single-conformer systems, *cis* conformers consistently exhibited dihedral angles near 0°, while *trans* conformers remained clustered around ±180°, indicating high conformational stability.^40^

This trend persisted across all *cis* : *trans* ratios in mixed-conformer systems, with each conformer maintaining its characteristic dihedral angle distribution. Notably, *cis* conformers displayed slightly broader dihedral angle distributions and greater variability in *cis* fraction, particularly at higher *cis* content, suggesting increased conformational flexibility within the membrane environment.^41^

**Fig. 5:**
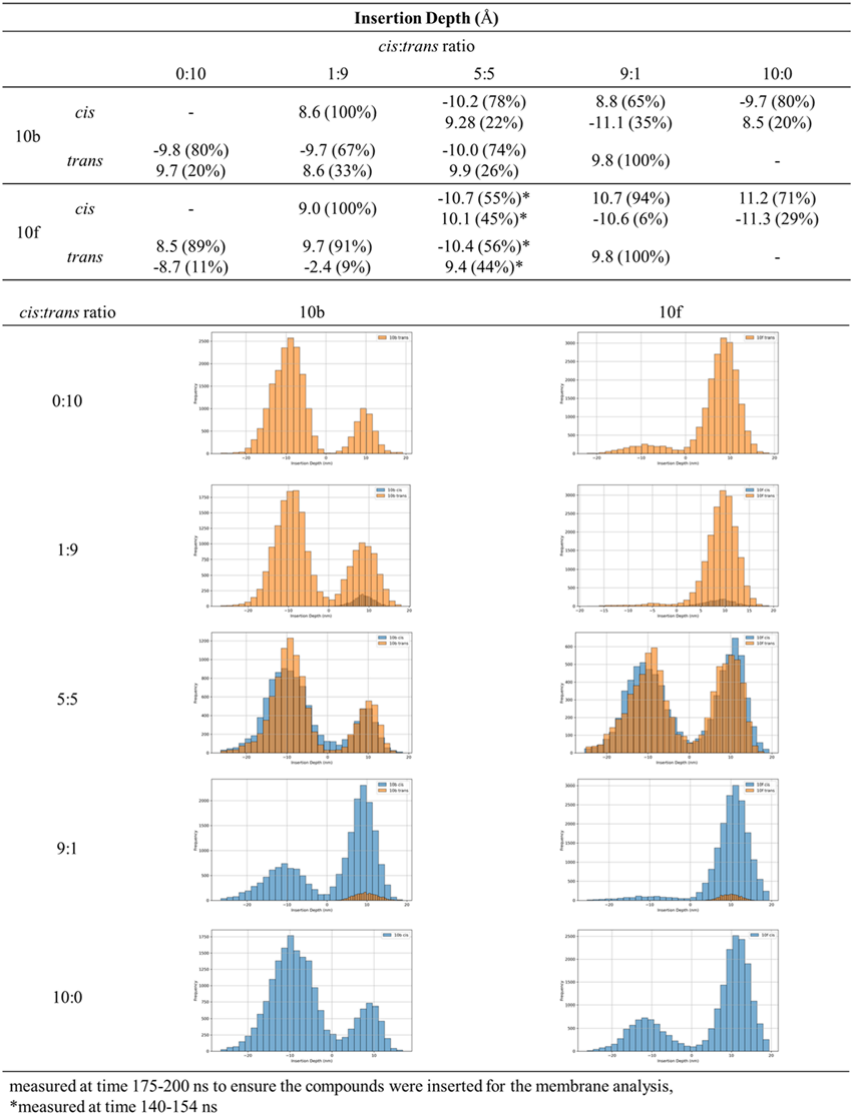
Membrane insertion profiles of compound 10b and 10f across varying *cis* : *trans* conformer ratios. Histograms demonstrate bimodal distributions with inserted (positive depths) and non-inserted (negative depths) indicating population distribution.

Compound 10f consistently demonstrated increased membrane insertion in comparison to compound 10b across all simulations (Fig. 5). The membrane insertion profiles reveal bimodal distributions for both compounds, indicating two distinct molecular populations: inserted into the hydrophobic tails of the lipid membrane (positive insertion depths) and non-inserted (negative insertion depths). Those with negative insertion depths may be interacting with the lipid headgroups.^42^ In single-conformer systems, for compound 10b, the majority population exhibited mean insertion depths of −9.7 Å (*cis*, 80%) and −9.8 Å (*trans*, 80%), indicating that most of the molecules remained outside the hydrophobic core of the membrane. However, a minor population showed membrane insertion with depths of 8.5 Å (*cis*, 20%) and 9.7 Å (*trans*, 20%). In contrast, compound 10f showed significantly enhanced insertion with the majority of the molecules successfully embedded within the membrane. The predominant population showed deeper insertion depths of 11.2 Å (*cis*, 71%) and 8.5 Å (*trans*, 89%), while the minority population remained outside the membrane at −11.3 Å (*cis*, 29%) and −8.7 Å (*trans*, 11%). This represents a fundamental difference in membrane affinity between the two compounds.^43^

Mixed conformer systems reinforced this trend across varying *cis* : *trans* ratios (1 : 9, 5 : 5, 9 : 1). Compound 10f consistently maintained higher proportions of inserted molecules and achieved greater insertion depths across all ratios. Interestingly, compound 10f at the 5 : 5 ratio showed reduced inserted population (44–45%) compared to the other ratios, suggesting that the ratio of the conformers present in solution may influence membrane insertion efficiency.

These trends were further supported by simulations of mixed *cis* : *trans* conformer ratios.

**Fig. 6:**
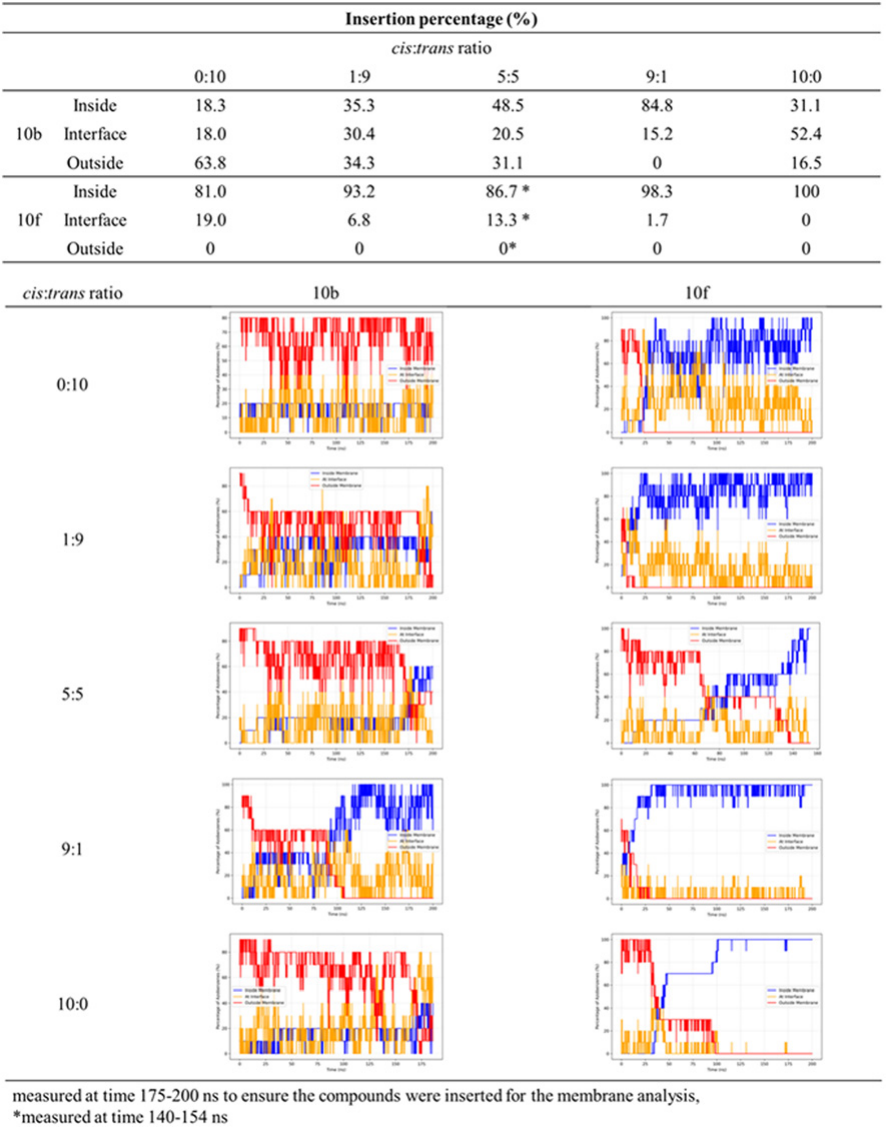
Spatial distribution of membrane insertion for compound 10b and 10f across varying *cis* : *trans* conformer ratios. Data presented as mean. Inside (blue) = molecules embedded within membrane, interface (orange) = at the membrane–water interface (±5 Å), outside (red) = in the aqueous phase.

Compound 10b was frequently found to remain in the aqueous or interfacial regions of the simulated systems during the simulations. Compound 10f demonstrated rapid and sustained membrane insertion, with molecules quickly achieving stable positions within the lipid bilayer core across most conformer ratios (Fig. 6). Compound 10b exhibited limited and variable insertion depending on the conformer ratio (Fig. 5). When only *trans* conformers were present, compound 10b showed that only 18.3 ± 0.3% of the molecules inserted into the hydrophobic core of the membranes, while another 18.0 ± 0.8% were found at the interface of the membrane. The system with the *cis*-dominant (9 : 1) ratio showed the highest percentage of inserted molecules (84.8 ± 0.7%), while the *cis*-only system showed 31.1 ± 0.9% of the molecules had inserted, and a significant proportion of the molecules (52.4 ± 0.9%) were found at the interface of the membrane. In contrast, compound 10f consistently exhibited superior membrane insertion across all conformer ratios. In the *trans*-only system, 81.0 ± 0.9% of the molecules inserted into the membrane and 19.0 ± 0.9% were found at the membrane interface. The *trans*-dominant (1 : 9) system showed 93.2 ± 0.4% insertion, while the *cis*-dominant (9 : 1) and *cis*-only (10 : 0) systems showed 98.3 ± 0.3% and 100.0% insertion, respectively. Interestingly, the equimolar (5 : 5) system exhibited fewer inserted molecules (86.7 ± 0.8%) compared to other systems, suggesting conformer-specific interactions that affect membrane penetration dynamics as demonstrated in the insertion depth analysis.

These results indicate that the enhanced hydrophobic character of compound 10f promotes consistent and deep membrane insertion, while compound 10b with limited hydrophobic characteristics results in conformer-dependent and often significantly reduced membrane association.^44^

**Fig. 7:**
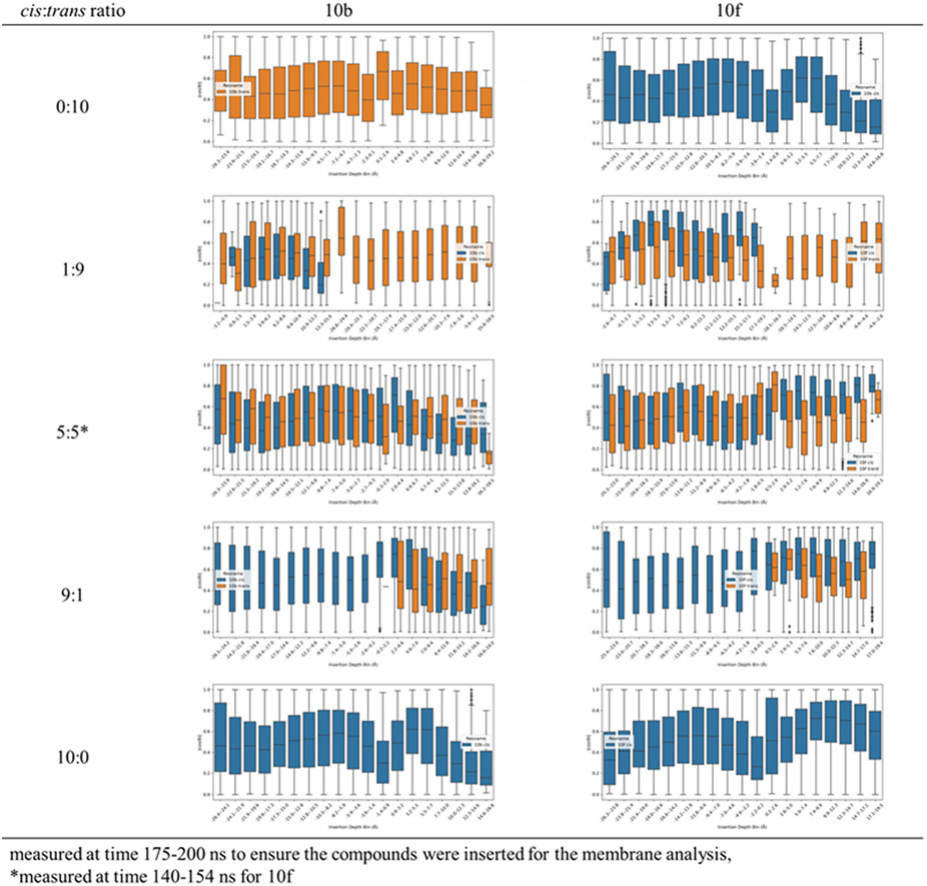
Molecular orientation as a function of membrane insertion depth for compounds 10b and 10f across varying *cis* : *trans* conformer ratios. Orientation values approaching 1.0 indicate upright molecular alignment, while values near 0 suggest tilted or horizontal orientations. Error bars represent SEM.

To further investigate how the conformational state affects the interaction of the compound with the membrane, the orientation of the compound was evaluated as a function of insertion depth (Fig. 7). In all the simulated systems, the *cis* conformer of both compounds exhibited larger orientation values at shallow insertion depths (0–5 Å), with values approaching 1, indicative of the primary axis of the compound being parallel to the normal of the membrane during initial membrane engagement.^45,46^ At deeper insertion depths (10–20 Å), compound 10b showed a decrease in orientation, suggesting a transition toward a more tilted orientation (or perpendicular) in relation to the normal vector of the membrane. The *trans* conformers also exhibited relatively large orientation values near the membrane interface, though this trend was less pronounced than in the *cis* conformers. In contrast, compound 10f maintained large orientation values across all depths for both conformers, indicating that it remains vertical within the membrane irrespective of insertion depth.

**Fig. 8:**
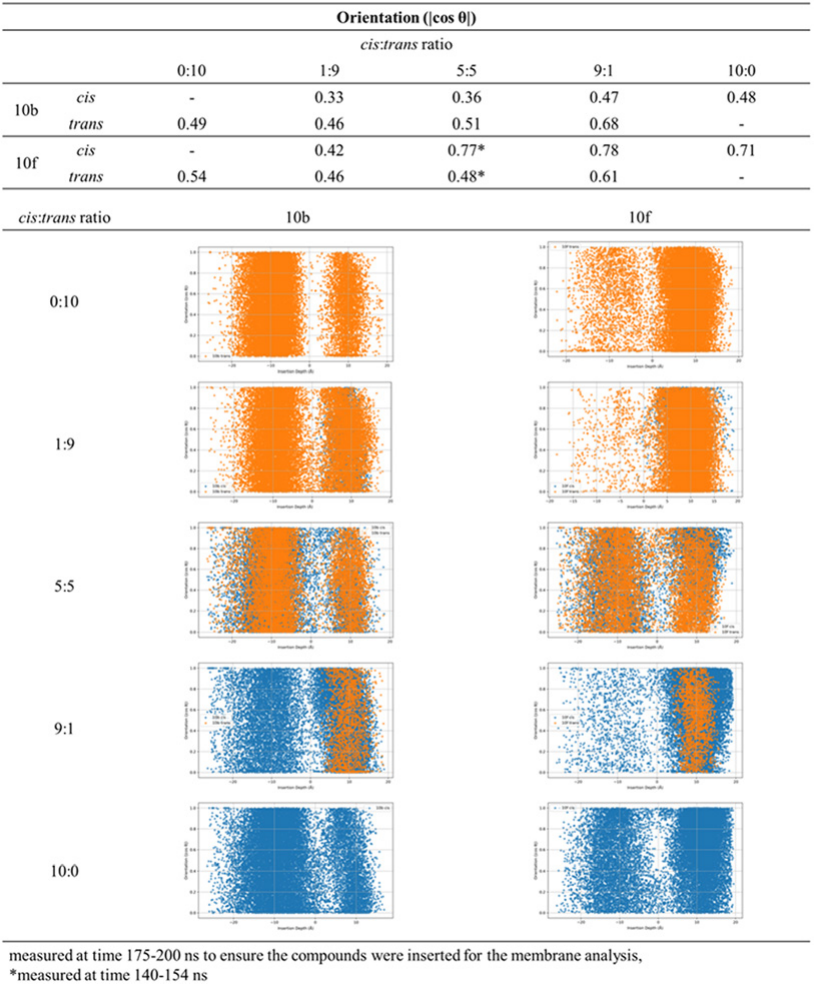
Molecular orientation analysis showing mean orientation values (|cos *θ*|) for compounds 10b and 10f across *cis* : *trans* conformer ratios.

This analysis revealed significant differences between the orientations taken by compounds 10b and 10f within the membrane (Fig. 8). For compound 10b, the *cis* conformer showed a moderate increase in mean orientation with increasing *cis* content (cos(*θ*) ∼ 0.33 to cos(*θ*) ∼ 0.48), while the *trans* conformer remained relatively stable across most of the *cis* : *trans* ratios (cos(*θ*) ∼ 0.46–0.51) but showed a notable increase in orientation in the systems with large *cis* ratios (cos(*θ*) ∼ 0.68). Compound 10f demonstrated markedly larger orientation values, with the *cis* conformer maintaining a consistently more tilted orientation (cos(*θ*) ∼ 0.42–0.78) and the *trans* conformer became less tilted with decreasing *trans* content (cos(*θ*) ∼ 0.46–0.61).

The differential antibacterial activity between compounds 10b and 10f correlates with their membrane insertion and orientation profiles. Compound 10f demonstrated up to 32-fold increase in antibacterial efficacy upon irradiation, corresponding to the *cis*-dominant conditions. This enhanced activity aligns with the results from the molecular dynamics simulations showing optimal membrane insertion (98.3% at 9 : 1 ratio, 100% at 10 : 0 ratio) and superior orientational stability (0.78 *cis* orientation) when most of the molecules were in a *cis* conformation. In contrast, compound 10b showed reduced antibacterial activity upon irradiation and showed only a modest reduction in potency, consistent with its limited and variable membrane insertion capabilities (maximum 84.8% at 9 : 1, only 31.1% at 10 : 0) and lower orientational alignment (maximum 0.48 for *cis* conformer).

The mechanistic basis for this structure–activity relationship lies in the membrane disruption requirements for antibacterial efficacy. Effective membrane disruption requires both deep membrane insertion and stable molecular orientation to create sufficient perturbation of the lipid bilayer integrity.^47,48^ Compound 10f achieved these critical parameters specifically in *cis*-dominant conditions, where high insertion percentages combined with organised spatial distribution and optimal molecular alignment created the necessary membrane destabilization for bacterial cell death.

Compound 10b, despite showing some membrane insertion and notable antibacterial activity in its *trans*-dominant state, lacked the consistent deep insertion and orientational stability required for effective membrane disruption. The superior hydrophobic character of compound 10f enables the sustained membrane integration necessary for antimicrobial mechanism of action, while compound 10b remains primarily at the interface without sufficient membrane disruption capacity to translate conformational changes into pronounced differences in antibacterial activity upon irradiation.

## Conclusions

This study demonstrates that the antibacterial activity of amphiphilic lipids can be modulated by light through controlled photoisomerisation of azobenzene cores. The synthesised photoswitchable amphiphilic lipids (PALs) exhibit clear, chain-length-dependent responses to irradiation, with short-chain derivatives losing potency upon UV exposure, whereas longer-chain analogues become markedly more active in their *cis*-enriched states. Molecular dynamics simulations reveal that these changes arise from light-dependent differences in membrane insertion depth and molecular orientation, providing a mechanistic explanation for the observed photomodulation. Together, these findings show that reversible structural changes in lipid amphiphiles can translate into tunable antibacterial outcomes, establishing a rational framework for designing light-responsive membrane-active agents. Beyond their antibacterial potential, the PALs offer a model system for studying how molecular conformation governs membrane interactions, with implications for broader applications of photochemical control in chemical biology, pharmacology and medicinal chemistry.

## Author contributions

G. F. S. F. and S.-H. K. performed the synthesis and microbiological evaluation of PALs and wrote of the manuscript; C. K. H., J. F.-P., J. R. performed the microbiology studies; S.-H. K. and C. D. L. performed the MD studies; A. J. M. and J. M. S. contributed to the supervision, conceptualization and design of the work; D. C. conceived and designed the project, contributed to the overall supervision of the team, and wrote the manuscript. All authors contributed to the preparation of this manuscript.

## Conflicts of interest

There are no conflicts to declare.

## Supplementary Material

MD-017-D6MD00096G-s001

## Data Availability

The data supporting this article, including synthetic procedures, compound characterisation data, UV-vis and NMR photoisomerisation studies, antibacterial assays, and molecular dynamics simulation methods, are available in the supplementary information (SI). Supplementary information is available. See DOI: https://doi.org/10.1039/d6md00096g.
