# Supplementary material for: Light-controlled inhibition of Gram-positive bacteria by photoswitchable amphiphilic lipids (PALs)
Source: RSC Med Chem. 2026 May 1;17(6):3042–53. doi: 10.1039/d6md00096g (PMC13215007; doi:10.1039/d6md00096g)
Supplement: MD-017-D6MD00096G-s001 [file MD-017-D6MD00096G-s001.pdf]

Supplementary Information (SI) for RSC Medicinal Chemistry.
This journal is © The Royal Society of Chemistry 2026

Light-Controlled Inhibition of Gram-Positive Bacteria by Photoswitchable Amphiphilic Lipids (PALs)

Guilherme F. S. Fernandes,[a,‡,†] Seong-Heun Kim,[b,‡,] Charlotte K. Hind,[c] Jessica Furner-Pardoe,[c] Janis Romanopulos,[b] Christian D. Lorenz,[d] A. James Mason,[b] J. Mark Sutton,[c] Daniele Castagnolo,[a,*]

[a.]Department of Chemistry, University College London, 20 Gordon Street, London WC1H 0AJ, United Kingdom. [b.]Institute of Pharmaceutical Science, School of Cancer & Pharmaceutical Science, King's College London, 150 Stamford Street, SE1 9NH, London, United Kingdom. [c.]Antimicrobial Discovery, Development and Diagnostics, Vaccine Development and Evaluation Centre, UKHSA Porton Down, Salisbury SP4 0JG, United Kingdom. [d.]Biological Physics and Soft Matter Group, Department of Physics, King's College London, London WC2R 2LS, United Kingdom

*Corresponding Author. d.castagnolo@ucl.ac.uk

†Present Address: Chemical Biology Science Technology Platform, The Francis Crick Institute, London, NW1 1AT, UK

‡These authors contributed equally.

Supplementary Information

Table of Content

1. Chemistry S2

2. Photoisomerization studies S17

3. Microbiology S27

4. Molecular dynamic simulation S30

5. References S30

6. NMR spectra S31

7. HLPC and HRMS spectra for compounds 10b and 10f S68

1. Chemistry

Unless otherwise stated, all reagents were obtained from commercial suppliers and used without further purification. Most chemical reactions were carried out under a nitrogen atmosphere in oven-dried glassware. Analytical thin-layer chromatography (TLC) was performed using commercially available pre-coated plates and visualized under UV light at 254 nm. Flash column chromatography was conducted on silica gel (Sigma-Aldrich, 40–63 µm particle size, 60 Å pore size). Reaction products were characterized by ^{1}H and ^{13}C NMR spectroscopy, where applicable, using an Ascend400 spectrometer (Bruker, Germany) in either DMSO-d_6 or $CDCl_3$ (δH 400 MHz, δC 101 MHz). Chemical shifts (δ) are reported in parts per million (ppm), referenced to residual solvent signals. Coupling constants (*J*) are given in hertz (Hz), and multiplicities are designated as follows: singlet (s), doublet (d), triplet (t), doublet of doublets (dd), doublet of quartets (dq), doublet of doublet of triplets (ddt), and multiplet (m). Accurate mass measurements were carried out using an ASAP-HESI ionisation source coupled to a Q Exactive Plus mass spectrometer.
Due to their very limited solubility in common deuterated solvents, compounds **10h**, **10i**, and **15a–15e** could not be fully characterised by ^{13}C NMR spectroscopy. In all cases, precipitation was observed during acquisition, leading to insufficient signal intensity. Structural assignment was therefore supported by ^{1}H NMR and HRMS data.

General procedure for the photoswitchable azobenzenes 8a-i

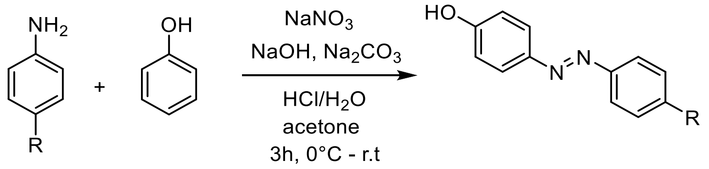

6a-i **8a-i**

In a 50 mL round-bottom flask, the corresponding aniline (1 eq.) was dissolved in a mixture of water and acetone (6 mL, 2:1 ratio) with the addition of HCl (2 mL). The resulting solution was stirred for 1 hour at 0°C. In a separate flask, sodium nitrite (1 eq.) was dissolved in water (2 mL), cooled to 0°C, and then added to the first solution. The combined mixture was stirred for 90 minutes at 0°C. In a separate 100 mL flask, phenol (1 eq.), NaOH (1 eq.), and Na_2CO_3 (1 eq.) were dissolved in water (7 mL) and stirred at room temperature. The diazonium salt solution obtained (noted by its purple colour) was added dropwise to this solution, maintaining a temperature below 5°C using an ice bath. The mixture was stirred for an additional hour. Upon completion, the product was extracted with dichloromethane, and the organic layer was washed with water (4 times) and brine. The solvent was then removed under reduced pressure, and the crude product was further purified using flash chromatography.

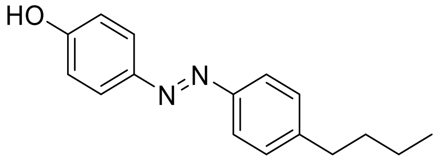

(*E*)-4-((4-butylphenyl)diazenyl)phenol (8a): Orange solid; yield 48%. ^{1}H NMR (400 MHz, Chloroform-*d*) δ: 7.86 (d, *J* = 8.6 Hz, 2H), 7.79 (d, *J* = 8.1 Hz, 2H), 7.30 (d, *J* = 8.0 Hz, 2H), 6.93 (d, *J* = 8.6 Hz, 2H), 2.68 (t, *J* = 7.7 Hz, 2H), 1.64 (dt, *J* = 15.3, 7.7 Hz, 2H), 1.43 – 1.33 (m, 2H), 0.94 (t, *J* = 7.3 Hz, 3H). ^{13}C NMR (100 MHz, Chloroform-*d*) δ: 158.1, 151.1, 147.4, 146, 129.2 (2C), 124.9 (2C), 122.6 (2C), 115.9 (2C), 35.7, 33.6, 22.4, 14.

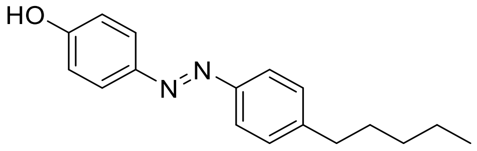

(*E*)-4-((4-pentylphenyl)diazenyl)phenol (8b): Orange solid; yield 50%. ^{1}H NMR (400 MHz, Chloroform-*d*) δ: 7.86 (d, *J* = 8.5 Hz, 2H), 7.80 (d, *J* = 8.1 Hz, 2H), 7.30 (d, *J* = 8.1 Hz, 2H), 6.93 (d, *J* = 8.6 Hz, 2H), 5.28 (s, 1H), 2.67 (t, *J* = 7.7 Hz, 2H), 1.70 – 1.61 (m, 4H), 1.39 – 1.29 (m, 4H), 0.90 (t, *J* = 6.5 Hz, 3H). ^{13}C NMR (100 MHz, Chloroform-*d*) δ: 160.3; 158.1, 151.0, 147.4, 146.1, 129.2 (2C), 124.9 (2C), 122.7 (2C), 115.9 (2C), 35.9, 31.6, 22.6, 14.1.

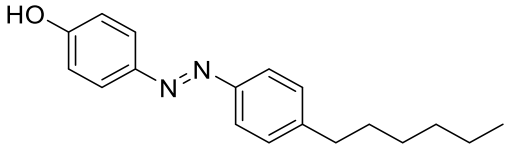

(*E*)-4-((4-hexylphenyl)diazenyl)phenol (8c): Orange solid; yield 48%. ^{1}H NMR (400 MHz, Chloroform-*d*) δ: 7.86 (d, *J* = 8.6 Hz, 2H), 7.80 (d, *J* = 8.1 Hz, 2H), 7.30 (d, *J* = 8.1 Hz, 2H), 6.93 (d, *J* = 8.6 Hz, 2H), 2.67 (t, *J* = 7.7 Hz, 2H), 1.65 (dt, *J* = 15.1, 7.5 Hz, 2H), 1.40 – 1.26 (m, 6H), 0.89 (t, *J* = 6.4 Hz, 3H). ^{13}C NMR (100 MHz, Chloroform-*d*) δ: 158.1, 151.0, 147.4, 146.1, 129.2 (2C), 124.9 (2C), 122.6 (2C), 115.9 (2C), 36, 31.8, 31.4, 22.7, 14.2.

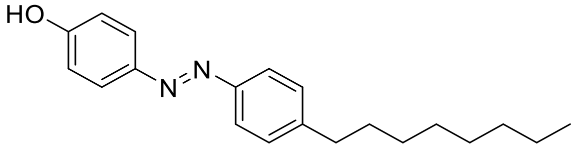

(*E*)-4-((4-octylphenyl)diazenyl)phenol (8d): Orange solid; yield 34%. ^{1}H NMR (400 MHz, Chloroform-*d*) δ: 7.86 (d, *J* = 8.4 Hz, 2H), 7.80 (d, *J* = 8.0 Hz, 2H), 7.30 (d, *J* = 8.0 Hz, 2H), 6.93 (d, *J* = 8.4 Hz, 2H), 2.67 (t, *J* = 7.7 Hz, 2H), 1.64 (dd, *J* = 14.3, 7.0 Hz, 2H), 1.38 – 1.22 (m, 10H), 0.88 (t, *J* = 6.5 Hz, 3H). ^{13}C NMR (100 MHz, Chloroform-*d*) δ: 158.1, 151.0, 147.3, 146.1, 129.2 (2C), 124.9 (2C), 122.6 (2C), 115.9 (2C), 36, 32, 31.4, 29.6, 29.4 (2C), 22.8, 14.2.

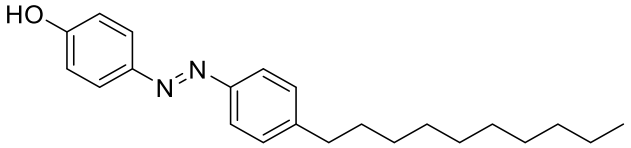

(*E*)-4-((4-decylphenyl)diazenyl)phenol (8e): Orange solid; yield 62%. ^{1}H NMR (400 MHz, Chloroform-*d*) δ: 7.86 (d, *J* = 8.4 Hz, 2H), 7.79 (d, *J* = 8.0 Hz, 2H), 7.30 (d, *J* = 8.0 Hz, 2H), 6.93 (d, *J* = 8.4 Hz, 2H), 2.67 (t, *J* = 7.7 Hz, 2H), 1.71 – 1.58 (m, 2H), 1.39 – 1.20 (m, 14H), 0.88 (t, *J* = 6.6 Hz, 3H). ^{13}C NMR (100 MHz, Chloroform-*d*) δ: 158, 151, 147.4, 146.1, 129.2 (2C), 124.9 (2C), 122.7 (2C), 115.9 (2C), 36, 32, 31.4, 29.7 (4C), 29.6, 29.4, 14.2.

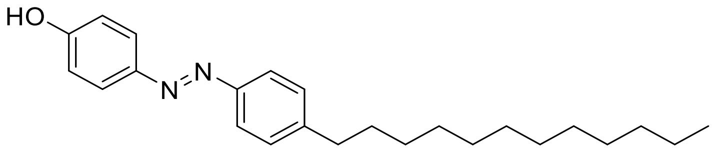

(*E*)-4-((4-dodecylphenyl)diazenyl)phenol (8f): Dark yellow solid; yield 22%. ^{1}H NMR (400 MHz, $CDCl_3$) δ: 7.88 (d, *J* = 8.8 Hz, 2H), 7.81 (d, *J* = 8.4 Hz, 2H), 7.30 (d, *J* = 8.5 Hz, 2H), 6.95 (d, *J* = 8.9 Hz, 2H), 2.66 (dd, *J* = 17.3, 9.4 Hz, 2H), 1.65 (dt, *J* = 15.0, 7.4 Hz, 2H), 1.29 – 1.23 (m, 18H), 0.88 (t, *J* = 6.9 Hz, 3H). ^{13}C NMR (100 MHz, $CDCl_3$) δ: 158.1, 151.1, 147.4, 146.1, 129.2 (2C), 124.9 (2C), 122.7 (2C), 115.9 (2C), 36.0, 32.0, 31.4, 29.8, 29.8, 29.7, 29.7, 29.6, 29.5, 29.4, 22.8, 14.2.

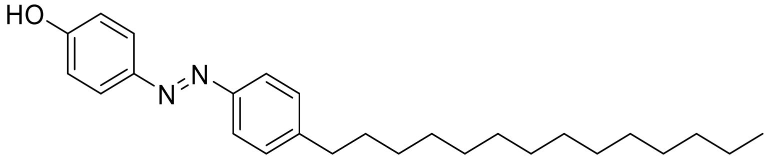

(*E*)-4-((4-tetradecylphenyl)diazenyl)phenol (8g): Orange solid; yield 69%. ^{1}H NMR (400 MHz, Chloroform-*d*) δ: 7.86 (d, *J* = 8.7 Hz, 2H), 7.80 (d, *J* = 8.1 Hz, 2H), 7.30 (d, *J* = 8.1 Hz, 2H), 6.93 (d, *J* = 8.7 Hz, 2H), 2.67 (t, *J* = 7.7 Hz, 2H), 1.69 – 1.61 (m, 2H), 1.36 – 1.24 (m, 22H), 0.88 (t, *J* = 6.7 Hz, 3H). ^{13}C NMR (100 MHz, Chloroform-*d*) δ: 158, 151, 147.3, 146, 129.1 (2C), 124.8 (2C), 122.6 (2C), 115.8 (2C), 35.9, 32, 31.4, 29.7 (5C), 29.6, 29.5, 29.4, 29.3, 22.7, 14.2.

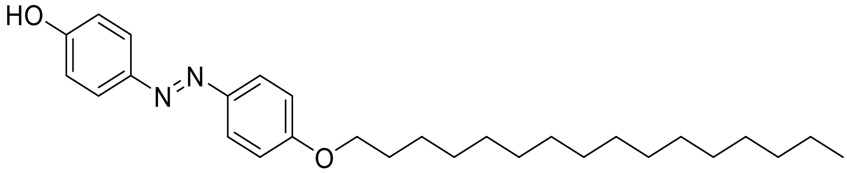

(*E*)-4-((4-(hexadecyloxy)phenyl)diazenyl)phenol (8h): Brown solid; yield 31%. ^{1}H NMR (400 MHz, DMSO-*d6*) δ: 10.20 (s, 1H), 7.78 (d, *J* = 8.7 Hz, 2H), 7.73 (d, *J* = 8.5 Hz, 2H), 7.07 (d, *J* = 8.7 Hz, 2H), 6.91 (d, *J* = 8.6 Hz, 2H), 4.04 (t, *J* = 6.3 Hz, 2H), 1.43 – 1.35 (m, 2H), 1.23 (s, 26H), 0.85

(t, J = 6.5 Hz, 3H). ^{13}C NMR (100 MHz, DMSO-$d6$) δ: 162.3, 160.4, 147.8, 147.2, 125.2 (2C), 124.9 (2C), 116.5 (2C), 115.6 (2C), 68.9, 33.8, 32.6, 30.3, 29.6, 26.7, 23.3, 14.3.

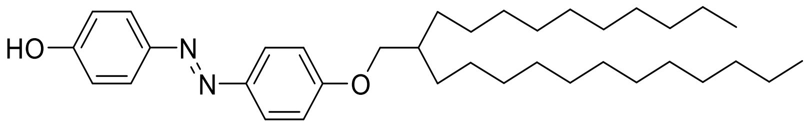

(*E*)-4-((4-((2-decyltetradecyl)oxy)phenyl)diazenyl)phenol (8i): Yellow oil; yield 36%. ^{1}H NMR (400 MHz, Chloroform-d) δ: 7.84 (dd, J = 12.2, 8.7 Hz, 4H), 6.99 (d, J = 8.7 Hz, 2H), 6.93 (d, J = 8.6 Hz, 2H), 3.90 (d, J = 5.6 Hz, 2H), 1.84 – 1.76 (m, J = 5.6 Hz, 1H), 1.37 – 1.21 (m, 40H), 0.88 (t, J = 6.6 Hz, 6H). ^{13}C NMR (100 MHz, Chloroform-d) δ: 161.7, 157.7, 147.7, 146.9, 124.6 (2C), 124.4 (2C), 115.8 (2C), 114.8 (2C), 71.4, 38, 32 (2C), 31.4 (2), 30 (2), 29.8 (10C), 26.9 (2C), 22.8 (2C), 14.2 (2C).

General procedure for the alkylation reaction to obtain derivatives 9a-i, 11a-c, 14a-e

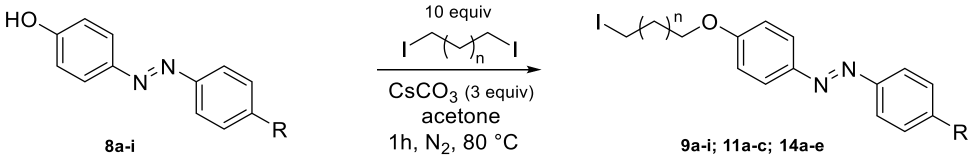

In a microwave flask, the relevant azobenzene compound (1 eq.) was dissolved in acetone. Subsequently, the corresponding diiodo alkyl compound was added in a significant excess (10 eq.), along with caesium carbonate (3 eq.). The mixture was then transferred to a microwave reactor and stirred for 1 hour at 80°C. Upon completion, the solvent was evaporated, and the residue was dissolved in DCM before being subjected to a series of washes with water and brine. The resulting crude product was subsequently purified using column chromatography.

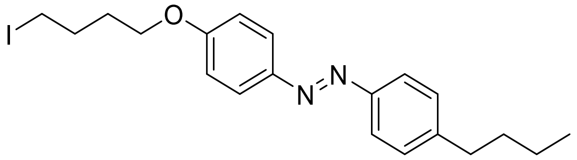

(*E*)-1-(4-butylphenyl)-2-(4-(4-iodobutoxy)phenyl)diazene (9a): Off-white solid; yield 49%. ^{1}H NMR (400 MHz, Chloroform-*d*) δ: 7.89 (d, J = 8.6 Hz, 2H), 7.80 (d, J = 8.0 Hz, 2H), 7.30 (d, J = 8.0 Hz, 2H), 6.99 (d, J = 8.6 Hz, 2H), 4.07 (t, J = 5.9 Hz, 2H), 3.28 (t, J = 6.7 Hz, 2H), 2.68 (t, J = 7.7 Hz, 2H), 2.11 – 1.90 (m, 4H), 1.69 – 1.59 (m, 2H), 1.44 – 1.33 (m, 2H), 0.94 (t, J = 7.3 Hz, 3H). ^{13}C NMR (100 MHz, Chloroform-*d*) δ: 161.2, 151.1, 147.2, 145.9, 129.2 (2C), 124.7 (2C), 122.6 (2C), 114.7 (2C), 67.1, 35.7, 33.6, 30.2 (2C), 22.4, 14.0, 6.4.

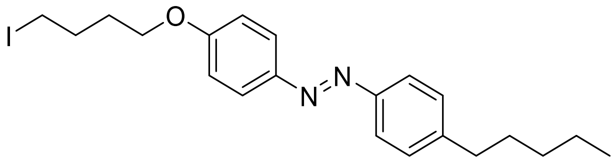

(*E*)-1-(4-(4-iodobutoxy)phenyl)-2-(4-pentylphenyl)diazene (9b): Orange solid; yield 46%. ^{1}H NMR (400 MHz, Chloroform-*d*) δ: 7.89 (d, *J* = 8.8 Hz, 2H), 7.80 (d, *J* = 8.1 Hz, 2H), 7.30 (d, *J* = 8.1 Hz, 2H), 6.99 (d, *J* = 8.8 Hz, 2H), 4.07 (t, *J* = 6.0 Hz, 2H), 3.28 (t, *J* = 6.7 Hz, 2H), 2.67 (t, *J* = 7.7 Hz, 2H), 2.10 – 1.91 (m, 4H), 1.67 (dd, *J* = 14.5, 7.3 Hz, 2H), 1.34 (m, 4H), 0.90 (t, *J* = 6.6 Hz, 3H). ^{13}C NMR (100 MHz, Chloroform-*d*) δ: 160.3; 161.2, 151.1, 147.2, 146.0, 129.2 (2C), 124.7 (2C), 122.6 (2C), 114.7 (2C), 67.1, 35.9, 31.6, 31.1, 30.2 (2C), 22.6, 14.1, 6.4.

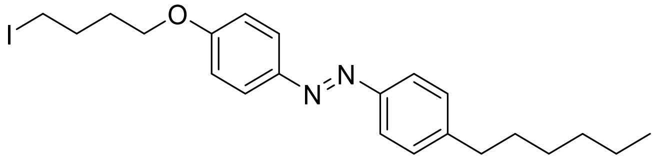

(*E*)-1-(4-hexylphenyl)-2-(4-(4-iodobutoxy)phenyl)diazene (9c): Orange solid; yield 49%. ^{1}H NMR (400 MHz, Chloroform-*d*) δ: 7.89 (d, *J* = 8.7 Hz, 2H), 7.80 (d, *J* = 8.1 Hz, 2H), 7.30 (d, *J* = 8.1 Hz, 2H), 6.99 (d, *J* = 8.7 Hz, 2H), 4.07 (t, *J* = 5.9 Hz, 2H), 3.28 (t, *J* = 6.7 Hz, 2H), 2.67 (t, *J* = 7.7 Hz, 2H), 2.11 – 1.91 (m, 4H), 1.70 – 1.61 (m, 2H), 1.39 – 1.27 (m, 6H), 0.89 (t, *J* = 6.4 Hz, 3H). ^{13}C NMR (100 MHz, Chloroform-*d*) δ: 161.2, 151.1, 147.2, 146.0, 129.1 (2C), 124.7 (2C), 122.6 (2C), 114.7 (2C), 67.1, 36.0, 31.8, 31.4, 30.2 (3C), 22.7, 14.2, 6.4.

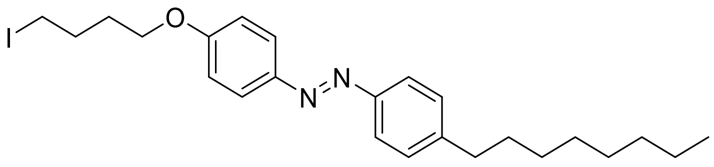

(*E*)-1-(4-(4-iodobutoxy)phenyl)-2-(4-octylphenyl)diazene (9d): Orange solid; yield 51%. ^{1}H NMR (400 MHz, Chloroform-*d*) δ: 7.89 (d, *J* = 8.7 Hz, 2H), 7.80 (d, *J* = 8.1 Hz, 2H), 7.30 (d, *J* = 8.1 Hz, 1H), 6.99 (d, *J* = 8.7 Hz, 2H), 4.07 (t, *J* = 5.9 Hz, 2H), 3.28 (t, *J* = 6.7 Hz, 2H), 2.67 (t, *J* = 7.7 Hz, 2H), 2.11 – 1.90 (m, 4H), 1.69 – 1.61 (m, 2H), 1.38 – 1.23 (m, 9H), 0.88 (t, *J* = 6.6 Hz, 3H). ^{13}C NMR (100 MHz, Chloroform-*d*) δ: 161.2, 151.1, 147.2, 146.0, 129.1 (2C), 124.7 (2C), 122.6 (2C), 114.7 (2C), 67.1, 36.0, 32.0, 31.4, 30.2 (3C), 29.6 (2C), 22.8, 14.2, 6.4.

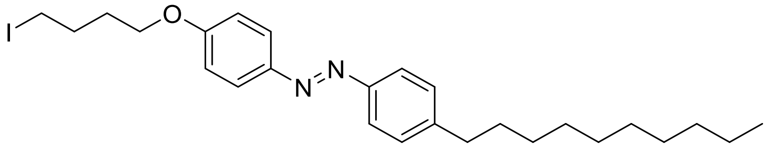

(*E*)-1-(4-decylphenyl)-2-(4-(4-iodobutoxy)phenyl)diazene (9e): Orange solid; yield 52%. ^{1}H NMR (400 MHz, Chloroform-*d*) δ: 7.89 (d, *J* = 8.8 Hz, 2H), 7.80 (d, *J* = 8.1 Hz, 2H), 7.30 (d, *J* = 8.1 Hz, 2H), 6.99 (d, *J* = 8.8 Hz, 2H), 4.07 (t, *J* = 6.0 Hz, 2H), 3.28 (t, *J* = 6.7 Hz, 2H), 2.67 (t, *J* = 7.7 Hz, 2H), 2.12 – 1.90 (m, 4H), 1.70 – 1.60 (m, 2H), 1.39 – 1.22 (m, 17H), 0.88 (t, *J* = 6.6 Hz, 3H). ^{13}C NMR (100 MHz, Chloroform-*d*) δ: 161.2, 151.1, 147.2, 146.0, 129.1 (2C), 124.7 (2C), 122.6 (2C), 114.7 (2C), 67.1, 36.0, 32.0, 31.4, 30.2 (3C), 29.7 (2C), 29.4 (2C), 22.8, 14.2, 6.3.

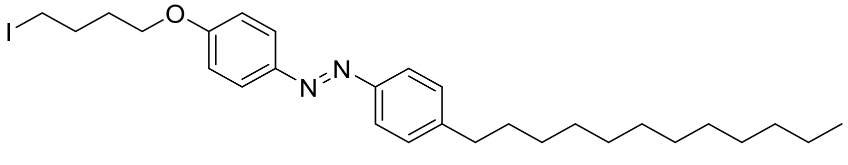

(*E*)-1-(4-dodecylphenyl)-2-(4-(4-iodobutoxy)phenyl)diazene (9f): Orange solid; yield 83%. ^{1}H NMR (400 MHz, Chloroform-*d*) δ: 7.89 (d, *J* = 8.7 Hz, 2H), 7.80 (d, *J* = 8.1 Hz, 2H), 7.30 (d, *J* = 8.1 Hz, 2H), 6.99 (d, *J* = 8.7 Hz, 2H), 4.07 (t, *J* = 5.9 Hz, 2H), 3.28 (t, *J* = 6.7 Hz, 2H), 2.67 (t, *J* = 7.6 Hz, 2H), 2.10 – 2.02 (m, 2H), 1.69 – 1.61 (m, 2H), 1.37 – 1.21 (m, 18H), 0.88 (t, *J* = 6.6 Hz, 3H). ^{13}C NMR (100 MHz, Chloroform-*d*) δ: 161.2, 151.1, 147.2, 146.0, 129.1 (2C), 124.7 (2C), 122.6 (2C), 114.7 (2C), 67.1, 36.0, 33.9 (3C), 32.0, 31.4, 30.2, 29.7 (2C), 29.6, 29.5, 29.4, 22.8, 14.2, 6.3.

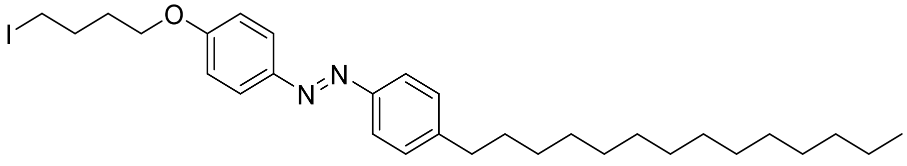

(*E*)-1-(4-(4-iodobutoxy)phenyl)-2-(4-tetradecylphenyl)diazene (9g): Orange solid; yield 58%. ^{1}H NMR (400 MHz, Chloroform-*d*) δ: 7.89 (d, *J* = 8.8 Hz, 2H), 7.80 (d, *J* = 8.1 Hz, 1H), 7.30 (d, *J* = 8.1 Hz, 2H), 6.99 (d, *J* = 8.8 Hz, 2H), 4.07 (t, *J* = 6.0 Hz, 2H), 3.28 (t, *J* = 6.7 Hz, 2H), 2.67 (t, *J* = 7.7 Hz, 2H), 2.11 – 1.90 (m, 2H), 1.70 – 1.60 (m, 2H), 1.36 – 1.22 (m, 21H), 0.88 (t, *J* = 6.7 Hz, 3H). ^{13}C NMR (100 MHz, Chloroform-*d*) δ: 161.2, 151.1, 147.2, 146.0, 129.1 (2C), 124.7 (2C), 122.6 (2C), 114.7 (2C), 67.1, 36.0, 32.0, 31.4, 30.2 (2C), 29.8 (5C), 29.7, 29.6, 29.5, 29.4, 22.8, 14.2, 6.4.

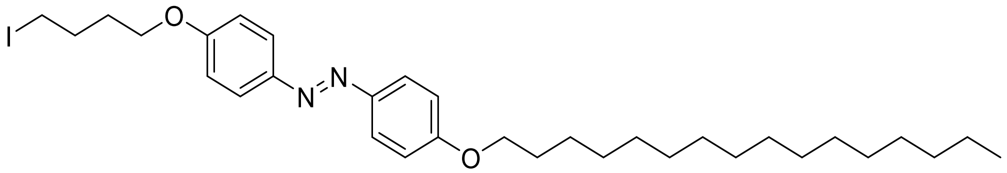

(*E*)-1-(4-(hexadecyloxy)phenyl)-2-(4-(4-iodobutoxy)phenyl)diazene (9h): Yellow solid; yield 15%. ^{1}H NMR (400 MHz, $CDCl_3$) δ: 7.89 – 7.82 (m, 4H), 6.99 (dd, *J* = 8.5, 5.5 Hz, 4H), 4.12 (t, *J* = 5.7 Hz, 2H), 4.03 (t, *J* = 6.6 Hz, 2H), 3.40 (t, *J* = 6.7 Hz, 2H), 2.32 (p, *J* = 6.2 Hz, 2H), 1.86 – 1.77 (m, 2H), 1.46 (dd, *J* = 14.5, 6.9 Hz, 2H), 1.40 – 1.14 (m, 23H), 0.88 (t, *J* = 6.6 Hz, 3H). ^{13}C NMR

(100 MHz, $CDCl_3$) δ: 160.7 (2C), 147.0 (2C), 124.5 (4C), 114.8 (4C), 68.4, 67.7, 33.0, 32.0, 29.8 (7C), 29.7 (2C), 29.5 (2C), 29.3, 26.1, 22.8, 14.2.

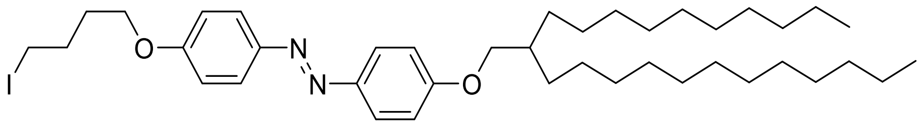

(*E*)-1-(4-((2-decyltetradecyl)oxy)phenyl)-2-(4-(4-iodobutoxy)phenyl)diazene (9i): Yellow oil. The compound was used on the following step without any purification.

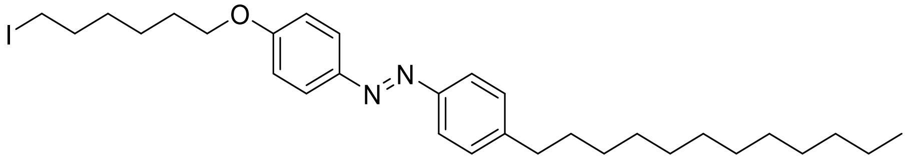

(*E*)-1-(4-dodecylphenyl)-2-(4-((6-iodohexyl)oxy)phenyl)diazene (11a): Orange solid; yield 35%. ^{1}H NMR (400 MHz, Chloroform-*d*) δ: 7.89 (d, *J* = 8.9 Hz, 2H), 7.79 (d, *J* = 8.2 Hz, 2H), 7.30 (d, *J* = 8.2 Hz, 2H), 6.99 (d, *J* = 8.9 Hz, 2H), 4.04 (t, *J* = 6.4 Hz, 2H), 3.22 (t, *J* = 7.0 Hz, 2H), 2.67 (t, *J* = 7.7 Hz, 2H), 1.91 – 1.80 (m, 4H), 1.70 – 1.61 (m, 2H), 1.54 – 1.48 (m, 4H), 1.33 – 1.22 (m, 17H), 0.88 (t, *J* = 6.9 Hz, 3H). ^{13}C NMR (100 MHz, Chloroform-*d*) δ: 161.4, 151.1, 147.1, 145.9, 129.1 (2C), 124.7 (2C), 122.6 (2C), 114.7 (2C), 68.1, 36.0, 33.5, 32.0, 31.4, 30.3, 29.8 (2C), 29.7 (2C), 29.6, 29.5, 29.4, 29.1, 25.2, 22.8, 14.2, 7.1.

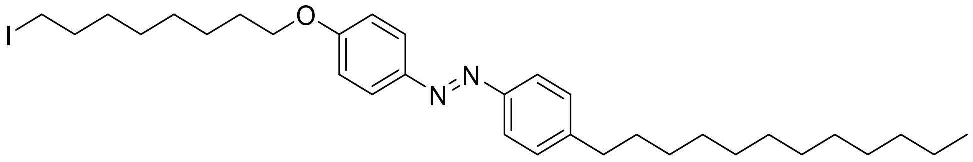

(*E*)-1-(4-dodecylphenyl)-2-(4-((8-iodooctyl)oxy)phenyl)diazene (11b): Orange solid; yield 33%. ^{1}H NMR (400 MHz, Chloroform-*d*) δ: 7.89 (d, *J* = 8.9 Hz, 2H), 7.79 (d, *J* = 8.3 Hz, 2H), 7.30 (d, *J* = 8.2 Hz, 2H), 6.99 (d, *J* = 8.9 Hz, 2H), 4.04 (t, *J* = 6.5 Hz, 2H), 3.20 (t, *J* = 7.0 Hz, 2H), 2.67 (t, *J* = 7.7 Hz, 2H), 1.87 – 1.78 (m, 4H), 1.68 – 1.61 (m, 2H), 1.46 – 1.23 (m, 25H), 0.88 (t, *J* = 6.9 Hz, 3H). ^{13}C NMR (100 MHz, Chloroform-*d*) δ: 161.4, 151.1, 147.0, 145.9, 129.1 (2C), 124.6 (2C), 122.6 (2C), 114.8 (2C), 68.3, 36.0, 33.6, 32.0, 31.4, 30.5, 29.8 (2C), 29.7 (3C), 29.6, 29.5, 29.4, 29.3 (2C), 26.0, 22.8, 14.2, 7.4.

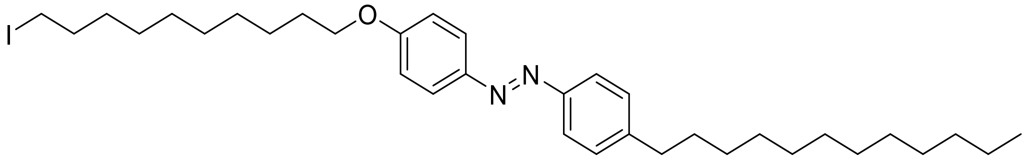

(*E*)-1-(4-dodecylphenyl)-2-(4-((10-iodododecyl)oxy)phenyl)diazene (11c): Orange solid; yield 43%. ^{1}H NMR (400 MHz, Chloroform-*d*) δ: 7.89 (d, *J* = 8.9 Hz, 2H), 7.79 (d, *J* = 8.3 Hz, 2H), 7.30 (d, *J* = 8.2 Hz, 2H), 6.99 (d, *J* = 8.9 Hz, 2H), 4.04 (t, *J* = 6.5 Hz, 2H), 3.19 (t, *J* = 7.0 Hz, 2H), 2.67 (t, *J* = 7.7 Hz, 2H), 1.86 – 1.78 (m, 4H), 1.69 – 1.61 (m, 2H), 1.51 – 1.45 (m, 2H), 1.37 – 1.23 (m, 27H), 0.88 (t, *J* = 6.9 Hz, 3H). ^{13}C NMR (100 MHz, Chloroform-*d*) δ: 161.5, 151.1, 147.0, 145.9, 129.1 (2C), 124.6 (2C), 122.6 (2C), 114.8 (2C), 68.4, 36.0, 33.6, 32.0, 31.4, 30.6, 29.8 (2C), 29.7 (3C), 29.6, 29.5 (2C), 29.4 (3C), 29.3, 26.1, 22.8, 14.2, 7.4.

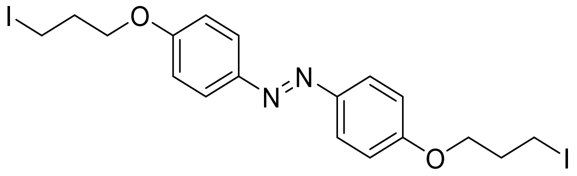

(*E*)-1,2-bis(4-(3-iodopropoxy)phenyl)diazene (14a): Off-white solid; yield 50%. ^{1}H NMR (400 MHz, Chloroform-*d*) δ: 8.21 (d, *J* = 9.0 Hz, 4H), 6.97 (d, *J* = 9.0 Hz, 4H), 4.15 (t, *J* = 5.8 Hz, 4H), 3.37 (t, *J* = 6.6 Hz, 4H), 2.31 (p, *J* = 6.2 Hz, 4H).

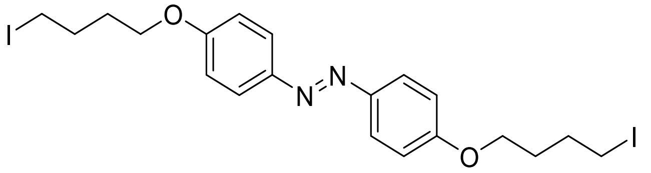

(*E*)-1,2-bis(4-(4-iodobutoxy)phenyl)diazene (14b): Off-white solid; yield 53%. ^{1}H NMR (400 MHz, Chloroform-*d*) δ: 8.20 (d, *J* = 9.1 Hz, 4H), 6.94 (d, *J* = 9.1 Hz, 4H), 4.09 (t, *J* = 5.9 Hz, 4H), 3.27 (t, *J* = 6.6 Hz, 4H), 2.08 – 1.92 (m, 8H). ^{13}C NMR (100 MHz, Chloroform-*d*) δ: 164.0 (2C), 141.7 (2C), 126.0 (4C), 114.5 (4C), 67.6 (2C), 30.0 (4C), 5.9 (2C).

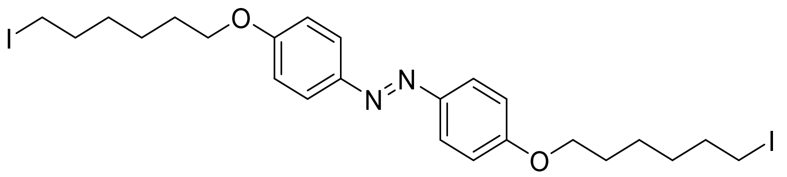

(*E*)-1,2-bis(4-((6-iodohexyl)oxy)phenyl)diazene (14c): Off-white solid; yield 42%. ^{1}H NMR (400 MHz, Chloroform-*d*) δ: 8.20 (d, *J* = 8.9 Hz, 4H), 6.94 (d, *J* = 8.9 Hz, 4H), 4.05 (t, *J* = 6.3 Hz, 4H), 3.21 (t, *J* = 6.9 Hz, 4H), 1.92 – 1.79 (m, 8H), 1.51 (dd, *J* = 13.9, 10.7 Hz, 8H). ^{13}C NMR (100 MHz, Chloroform-*d*) δ: 164.4 (2C), 141.5 (2C), 126.0 (4C), 114.5 (4C), 68.7 (2C), 33.3 (2C), 30.2 (2C), 28.9 (2C), 25.0 (2C), 6.9 (2C).

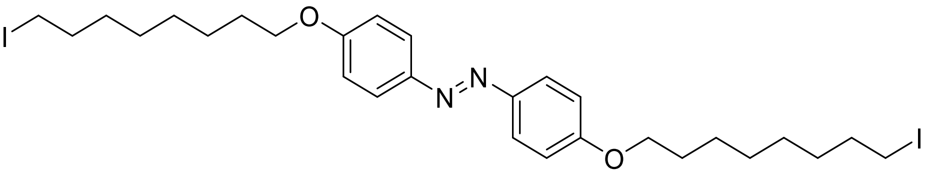

(*E*)-1,2-bis(4-((8-iodooctyl)oxy)phenyl)diazene (14d): Off-white solid; yield 59%. ^{1}H NMR (400 MHz, Chloroform-*d*) δ: 8.19 (d, *J* = 9.0 Hz, 4H), 6.94 (d, *J* = 9.1 Hz, 4H), 4.04 (t, *J* = 6.4 Hz, 4H), 3.19 (t, *J* = 7.0 Hz, 4H), 1.87 – 1.78 (m, 8H), 1.52 – 1.33 (m, 16H). ^{13}C NMR (100 MHz, Chloroform-*d*) δ: 164.3 (2C), 141.4 (2C), 126.0 (4C), 114.5 (4C), 68.7 (2C), 33.5 (2C), 30.5 (2C), 29.2 (2C), 29.0 (2C), 28.5 (2C), 25.9 (2C), 7.3 (2C).

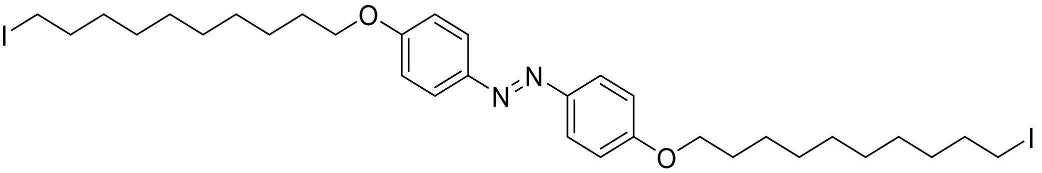

(*E*)-1,2-bis(4-((10-iododecyl)oxy)phenyl)diazene (14e): Off-white solid; yield 46%. ^{1}H NMR (400 MHz, Chloroform-d) δ: 8.19 (d, J = 8.9 Hz, 4H), 6.94 (d, J = 8.9 Hz, 4H), 4.04 (t, J = 6.5 Hz, 4H), 3.19 (t, J = 7.0 Hz, 4H), 1.86 – 1.77 (m, 8H), 1.49 – 1.25 (m, 24H). ^{13}C NMR (100 MHz, Chloroform-d) δ: 164.3 (2C), 141.4 (2C), 126.0 (4C), 114.5 (4C), 69.0 (2C), 33.6 (2C), 30.6 (2C), 29.5 (2C), 29.4 (2C), 29.3 (2C), 29.1 (2C), 28.6 (2C), 26.0 (2C), 7.4 (2C).

General procedure for the sSynthesis of the final compounds 10a-i, 12a-c, 15a-e

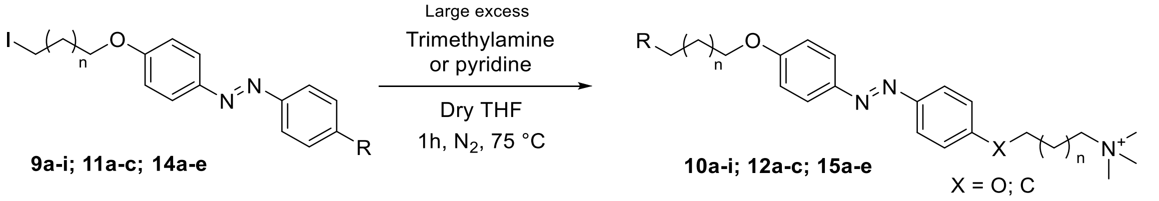

In a microwave flask, the respective azobenzene compound (1 eq.) was dissolved in a solution of trimethylamine in ethanol or pyridine. The mixture was then transferred to a microwave reactor and stirred for 1 hour at 75°C. Upon reaching completion, the solvent was evaporated, leaving behind the pure precipitated product, which was collected and subsequently dried.

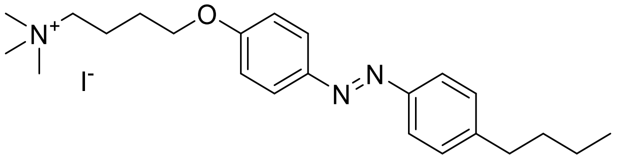

(*E*)-4-(4-((4-butylphenyl)diazenyl)phenoxy)-*N*,*N*,*N*-trimethylbutan-1-aminium iodide (10a): Yellow solid; yield 99%. ^{1}H NMR (400 MHz, DMSO-*d6*) δ: 7.88 (d, *J* = 8.8 Hz, 2H), 7.77 (d, *J* =

8.0 Hz, 2H), 7.39 (d, *J* = 8.1 Hz, 2H), 7.14 (d, *J* = 8.8 Hz, 2H), 4.14 (t, *J* = 5.9 Hz, 2H), 3.42 – 3.35 (m, 2H), 3.07 (s, 9H), 2.67 (t, *J* = 7.6 Hz, 2H), 1.92 – 1.73 (m, 4H), 1.64 – 1.53 (m, 2H), 1.37 – 1.27 (m, 2H), 0.91 (t, *J* = 7.3 Hz, 3H). ^{13}C NMR (100 MHz, DMSO-*d6*) δ: 160.9, 150.2, 146.2, 145.7, 129.2 (2C), 124.3 (2C), 122.2 (2C), 115.0 (2C), 67.1, 64.9, 52.2 (3C), 34.6, 32.9, 25.5, 21.7, 19.2, 13.7.

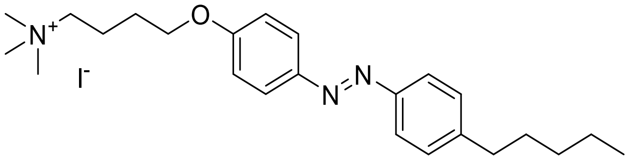

(*E*)-*N*,*N*,*N*-trimethyl-4-(4-((4-pentylphenyl)diazenyl)phenoxy)butan-1-aminium iodide (10b): Yellow solid; yield 99%. ^{1}H NMR (400 MHz, DMSO-*d6*) δ: 7.88 (d, *J* = 8.6 Hz, 2H), 7.77 (d, *J* = 8.0 Hz, 2H), 7.39 (d, *J* = 8.1 Hz, 2H), 7.14 (d, *J* = 8.6 Hz, 2H), 4.14 (t, *J* = 5.8 Hz, 2H), 3.42 – 3.36 (m, 2H), 3.07 (s, 9H), 2.66 (t, *J* = 7.6 Hz, 2H), 1.93 – 1.84 (m, 2H), 1.82 – 1.75 (m, 2H), 1.62 (dd, *J* = 14.5, 7.4 Hz, 2H), 1.33 – 1.27 (m, *J* = 2.8 Hz, 2H), 0.87 (t, *J* = 6.7 Hz, 3H). ^{13}C NMR (100 MHz, DMSO-*d6*) δ: 160.9, 150.2, 146.2, 145.7, 129.2 (2C), 124.3 (2C), 122.2 (2C), 115.0 (2C), 67.1, 64.9, 52.2 (3C), 34.9, 30.8, 30.4, 25.5, 21.9, 19.2, 13.9. HRMS (ESI) m/z: $[M + H]^+$ Calcd for $C_{24}H_{36}N_3O^+$ 382.2853; Found 382.2850.

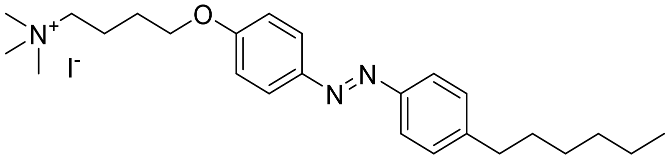

(*E*)-4-(4-((4-hexylphenyl)diazenyl)phenoxy)-*N*,*N*,*N*-trimethylbutan-1-aminium iodide (10c): Yellow solid; yield 99%. ^{1}H NMR (400 MHz, DMSO-*d6*) δ: 7.88 (d, *J* = 8.7 Hz, 2H), 7.76 (d, *J* = 8.0 Hz, 2H), 7.38 (d, *J* = 8.1 Hz, 2H), 7.14 (d, *J* = 8.7 Hz, 2H), 4.14 (t, *J* = 5.8 Hz, 2H), 3.42 – 3.36 (m, 2H), 3.08 (s, 9H), 2.66 (t, *J* = 7.6 Hz, 2H), 1.93 – 1.74 (m, *J* = 7.6 Hz, 4H), 1.64 – 1.56 (m, 2H), 1.28 (s, 6H), 0.85 (t, *J* = 6.3 Hz, 3H). ^{13}C NMR (100 MHz, DMSO-*d6*) δ: 160.9, 150.2, 146.2, 145.7, 129.2 (2C), 124.3 (2C), 122.2 (2C), 115.0 (2C), 67.1, 64.9, 52.2 (3C), 34.9, 31.0, 30.6, 29.2, 25.5, 25.5, 22.0, 19.2, 13.9. HRMS (ESI) m/z: $[M + H]^+$ Calcd for $C_{25}H_{38}N_3O^+$ 396.3009; Found 396.3001.

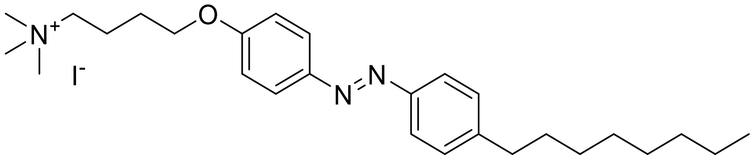

(*E*)-*N*,*N*,*N*-trimethyl-4-(4-((4-octylphenyl)diazenyl)phenoxy)butan-1-aminium iodide (10d): Yellow solid; yield 99%. ^{1}H NMR (400 MHz, DMSO-*d6*) δ: 7.88 (d, *J* = 8.6 Hz, 2H), 7.76 (d, *J* = 8.0 Hz, 2H), 7.38 (d, *J* = 8.0 Hz, 2H), 7.14 (d, *J* = 8.7 Hz, 2H), 4.14 (t, *J* = 5.8 Hz, 2H), 3.43 – 3.35 (m, 2H), 3.08 (s, 9H), 2.65 (t, *J* = 7.5 Hz, 2H), 1.94 – 1.74 (m, 4H), 1.66 – 1.55 (m, 2H), 1.31 – 1.20 (m, *J* = 20.5 Hz, 10H), 0.85 (t, *J* = 6.4 Hz, 3H). ^{13}C NMR (100 MHz, DMSO-*d6*) δ: 160.9, 150.2, 146.2, 145.7, 129.2 (2C), 124.3 (2C), 122.2 (2C), 115.0 (2C), 67.1, 64.9, 52.2 (3C), 34.9, 31.2, 30.7, 28.7, 28.6 (2C), 25.5, 22.0, 19.2, 13.9. HRMS (ESI) m/z: $[M + H]^+$ Calcd for $C_{27}H_{42}N_3O^+$ 424.3322; Found 424.3317.

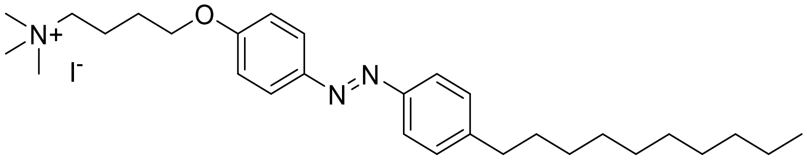

(*E*)-4-(4-((4-decylphenyl)diazenyl)phenoxy)-*N*,*N*,*N*-trimethylbutan-1-aminium iodide (10e): Yellow solid; yield 99%. ^{1}H NMR (400 MHz, DMSO-*d6*) δ: 7.88 (d, *J* = 8.6 Hz, 2H), 7.76 (d, *J* = 8.0 Hz, 2H), 7.38 (d, *J* = 8.0 Hz, 2H), 7.14 (d, *J* = 8.6 Hz, 2H), 4.14 (t, *J* = 5.8 Hz, 2H), 3.41 – 3.36 (m, 2H), 3.07 (s, 9H), 2.65 (t, *J* = 7.5 Hz, 2H), 1.93 – 1.75 (m, 4H), 1.65 – 1.56 (m, 2H), 1.31 – 1.19 (m, 14H), 0.84 (t, *J* = 6.4 Hz, 3H). ^{13}C NMR (100 MHz, DMSO-*d6*) δ: 160.9, 150.2, 146.2, 145.7, 129.2 (2C), 124.3 (2C), 122.2 (2C), 115.0 (2C), 67.1, 64.9, 52.2 (3C), 34.9, 31.2, 30.7, 28.9 (2C), 28.8, 28.6, 28.5, 25.5, 22.0, 19.2, 13.9. HRMS (ESI) m/z: $[M + H]^+$ Calcd for $C_{29}H_{46}N_3O^+$ 452.3635; Found 452.3611.

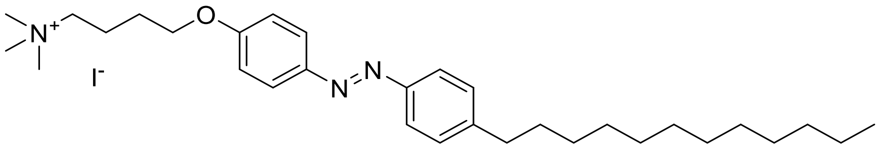

(*E*)-4-(4-((4-dodecylphenyl)diazenyl)phenoxy)-*N*,*N*,*N*-trimethylbutan-1-aminium iodide (10f): Yellow solid; yield 99%. ^{1}H NMR (400 MHz, DMSO-*d6*) δ: 7.87 (d, *J* = 8.6 Hz, 2H), 7.76 (d, *J* = 8.0 Hz, 2H), 7.38 (d, *J* = 8.1 Hz, 2H), 7.14 (d, *J* = 8.7 Hz, 2H), 4.14 (t, *J* = 5.8 Hz, 2H), 3.49 – 3.35 (m, 2H), 3.07 (s, 9H), 2.65 (t, *J* = 7.5 Hz, 2H), 1.94 – 1.75 (m, 4H), 1.60 (s, 2H), 1.31 – 1.16 (m, 18H), 0.84 (t, *J* = 6.5 Hz, 2H). ^{13}C NMR (100 MHz, DMSO-*d6*) δ: 160.9, 150.2, 146.2, 145.7, 129.2 (2C), 124.3 (2C), 122.2 (2C), 115.0 (2C), 67.2, 64.3, 52.2 (3C), 34.9, 31.2, 30.6, 29.9 (4C), 28.7, 28.6, 28.5, 25.5, 22.0, 19.3, 13.9. HRMS (ESI) m/z: $[M + H]^+$ Calcd for $C_{31}H_{50}N_3O^+$ 480.3948; Found 480.3937.

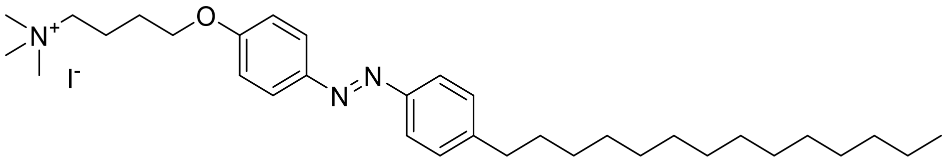

(*E*)-*N*,*N*,*N*-trimethyl-4-(4-((4-tetradecylphenyl)diazenyl)phenoxy)butan-1-aminium iodide (10g): Yellow solid; yield 99%. ^{1}H NMR (400 MHz, DMSO-*d6*) δ: 7.88 (d, *J* = 8.6 Hz, 2H), 7.76 (d, *J* = 8.0 Hz, 2H), 7.38 (d, *J* = 8.1 Hz, 2H), 7.14 (d, *J* = 8.7 Hz, 2H), 4.14 (t, *J* = 5.8 Hz, 2H), 3.41 – 3.36 (m, 2H), 3.07 (s, 9H), 2.65 (t, *J* = 7.4 Hz, 2H), 1.94 – 1.73 (m, 4H), 1.66 – 1.54 (m, 2H), 1.32 – 1.18 (m, 22H), 0.84 (t, *J* = 6.5 Hz, 3H). ^{13}C NMR (100 MHz, DMSO-*d6*) δ: 160.9, 150.2, 146.2, 145.7, 129.2 (2C), 124.3 (2C), 122.2 (2C), 115.0 (2C), 67.1, 64.9, 52.2 (3C), 34.9, 31.2, 30.6, 28.9 (6C), 28.7, 28.6, 28.5, 25.5, 22.0, 19.2, 13.9. HRMS (ESI) m/z: $[M + H]^+$ Calcd for $C_{33}H_{54}N_3O^+$ 508.4261; Found 508.4260.

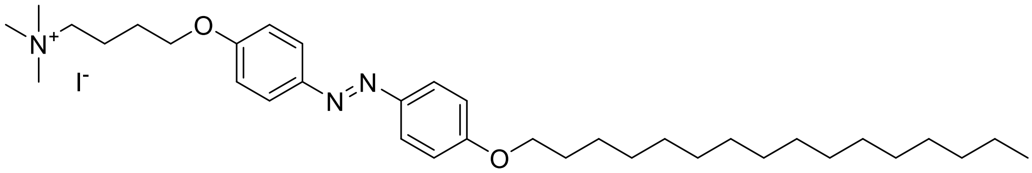

(*E*)-4-(4-((4-(hexadecyloxy)phenyl)diazenyl)phenoxy)-*N*,*N*,*N*-trimethylbutan-1-aminium iodide (10h): Yellow solid; yield 99%. ^{1}H NMR (400 MHz, DMSO-*d6*) δ: 7.84 (dd, *J* = 12.4, 8.8 Hz, 4H), 7.11 (dd, *J* = 14.0, 8.8 Hz, 4H), 4.17 (t, *J* = 5.6 Hz, 2H), 4.06 (t, *J* = 6.3 Hz, 2H), 3.64 – 3.42 (m, 2H), 3.11 (s, 9H), 2.28 (d, *J* = 41.1 Hz, 2H), 1.82 – 1.69 (m, 2H), 1.42 (s, 2H), 1.23 (m, 24H), 0.84 (t, *J* = 6.4 Hz, 3H). HRMS (ESI) m/z: $[M + H]^+$ Calcd for $C_{34}H_{56}N_3O^+$ 538.4367; Found 538.4357.

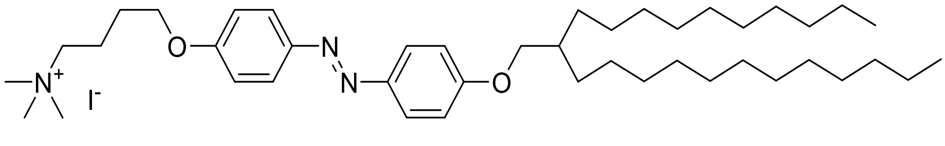

(*E*)-4-(4-((4-((2-decyltetradecyl)oxy)phenyl)diazenyl)phenoxy)-*N*,*N*,*N*-trimethylbutan-1-aminium iodide (10i): Yellow solid; yield 99%. ^{1}H NMR (400 MHz, DMSO-*d6*) δ: 7.83 (t, *J* = 9.7 Hz, 4H), 7.11 (dd, *J* = 12.4, 9.0 Hz, 4H), 4.13 (t, *J* = 5.7 Hz, 2H), 3.94 (d, *J* = 5.2 Hz, 2H), 3.41 – 3.35 (m, 2H), 3.07 (s, 9H), 1.93 – 1.72 (m, 4H), 1.35 – 1.15 (m, 40H), 0.83 (t, *J* = 6.4 Hz, 3H).

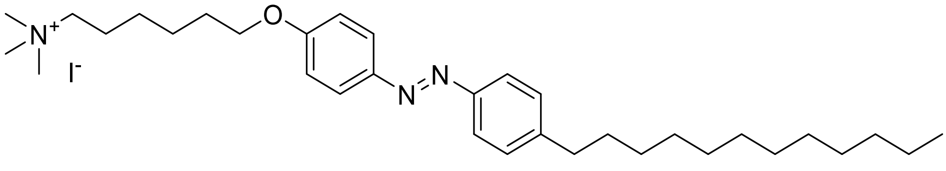

(*E*)-6-(4-((4-dodecylphenyl)diazenyl)phenoxy)-*N*,*N*,*N*-trimethylhexan-1-aminium iodide (12a): Yellow solid; yield 99%. ^{1}H NMR (400 MHz, DMSO-*d6*) δ: 7.86 (d, *J* = 8.7 Hz, 2H), 7.76 (d, *J* = 8.1 Hz, 2H), 7.38 (d, *J* = 8.1 Hz, 2H), 7.11 (d, *J* = 8.8 Hz, 2H), 4.09 (t, *J* = 6.2 Hz, 2H), 3.31 – 3.25

(m, 2H), 3.04 (s, 9H), 2.65 (t, J = 7.3 Hz, 2H), 1.83 – 1.68 (m, 4H), 1.60 (m, 2H), 1.49 (dd, J = 14.5, 7.4 Hz, 2H), 1.32 – 1.17 (m, 20H), 0.84 (t, J = 6.4 Hz, 3H). ^{13}C NMR (100 MHz, DMSO-*d6*) δ: 161.1, 150.2, 146.0, 145.7, 129.2 (2C), 124.3 (2C), 122.2 (2C), 114.9 (2C), 67.8, 65.2, 52.1 (3C), 35.1, 31.4, 30.7, 28.9 (3C), 28.7, 28.6, 28.5, 28.2, 25.4, 25.0, 22.0, 21.9, 13.9.

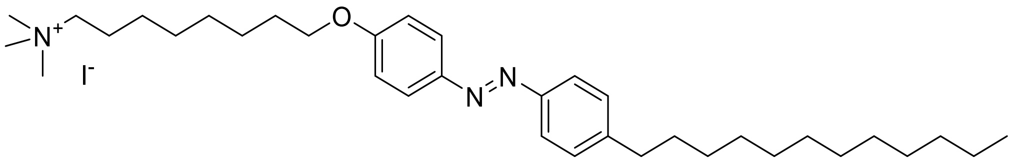

(*E*)-8-(4-((4-dodecylphenyl)diazenyl)phenoxy)-*N*,*N*,*N*-trimethyloctan-1-aminium iodide (12b): Yellow solid; yield 99%. ^{1}H NMR (400 MHz, DMSO-*d6*) δ: 7.86 (d, J = 8.6 Hz, 2H), 7.76 (d, J = 8.0 Hz, 2H), 7.38 (d, J = 8.0 Hz, 2H), 7.11 (d, J = 8.7 Hz, 2H), 4.08 (t, J = 6.3 Hz, 2H), 3.30 – 3.23 (m, 2H), 3.03 (s, 9H), 2.65 (t, J = 7.4 Hz, 2H), 1.80 – 1.56 (m, 6H), 1.44 (s, 2H), 1.37 – 1.18 (m, 24H), 0.84 (t, J = 6.4 Hz, 3H). ^{13}C NMR (100 MHz, DMSO-*d6*) δ: 161.1, 150.2, 146.0, 145.7, 129.2 (2C), 124.3 (2C), 122.2 (2C), 114.9 (2C), 67.9, 65.2, 52.1 (3C), 34.9, 31.2, 30.7, 28.9 (4C), 28.7, 28.6, 28.5 (2C), 28.4, 28.4, 25.6, 25.3, 22.0 (2C), 13.9.

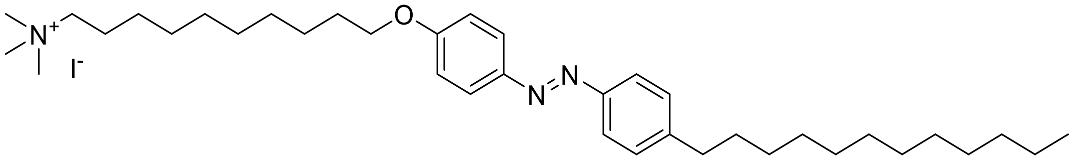

(*E*)-10-(4-((4-dodecylphenyl)diazenyl)phenoxy)-*N*,*N*,*N*-trimethyldecan-1-aminium iodide (12c): Yellow solid; yield 99%. ^{1}H NMR (400 MHz, DMSO-*d6*) δ: 7.85 (d, J = 8.7 Hz, 2H), 7.76 (d, J = 8.0 Hz, 2H), 7.37 (d, J = 8.1 Hz, 2H), 7.11 (d, J = 8.7 Hz, 2H), 4.07 (t, J = 6.3 Hz, 2H), 3.29 – 3.21 (m, 2H), 3.03 (s, 9H), 2.65 (t, J = 7.4 Hz, 2H), 1.78 – 1.56 (m, 6H), 1.43 (s, 2H), 1.35 – 1.18 (m, 28H), 0.84 (t, J = 6.5 Hz, 3H). ^{13}C NMR (100 MHz, DMSO-*d6*) δ: 161.2, 150.2, 146.0, 145.6, 129.1 (2C), 124.3 (2C), 122.2 (2C), 114.9 (2C), 67.9, 65.2, 52.1 (3C), 34.9, 31.2, 30.7, 28.9 (6C), 28.8, 28.7, 28.6 (2C), 28.5, 28.4, 25.7, 25.4, 22.0 (2C), 13.9.

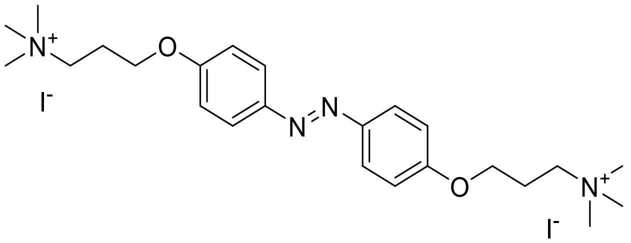

(*E*)-3,3'-((diazene-1,2-diylbis(4,1-phenylene))bis(oxy))bis(*N*,*N*,*N*-trimethylpropan-1-aminium) di-iodide (15a): Yellow solid; yield 99%. ^{1}H NMR (400 MHz, MeOD) δ: 8.24 (d, J = 9.0 Hz, 4H),

7.15 (d, *J* = 9.1 Hz, 4H), 4.28 (t, *J* = 5.6 Hz, 4H), 3.70 – 3.61 (m, 4H), 3.24 (s, 18H), 2.38 (dd, *J* = 9.8, 5.9 Hz, 4H).

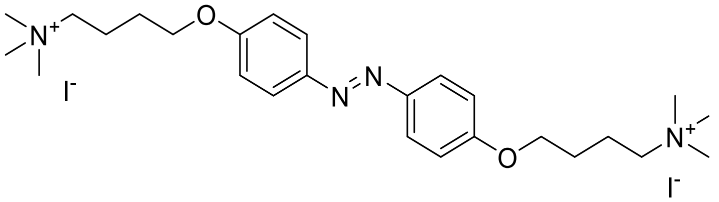

(*E*)-4,4'-((diazene-1,2-diylbis(4,1-phenylene))bis(oxy))bis(*N*,*N*,*N*-trimethylbutan-1-aminium) di-iodide (15b): Yellow solid; yield 99%. ^{1}H NMR (400 MHz, DMSO-*d6*) δ: 8.22 (d, *J* = 9.1 Hz, 4H), 7.17 (d, *J* = 9.1 Hz, 4H), 4.18 (t, *J* = 5.8 Hz, 4H), 3.41 – 3.35 (m, 4H), 3.08 – 3.04 (m, *9*H), 1.92 – 1.74 (m, 8H).

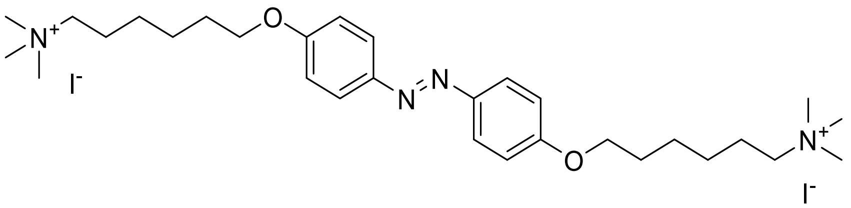

(*E*)-6,6'-((diazene-1,2-diylbis(4,1-phenylene))bis(oxy))bis(*N*,*N*,*N*-trimethylhexan-1-aminium) di-iodide (15c): Yellow solid; yield 99%. ^{1}H NMR (400 MHz, DMSO-*d6*) δ: 8.22 (d, *J* = 9.1 Hz, 4H), 7.09 (d, *J* = 9.1 Hz, 4H), 4.15 (t, *J* = 6.3 Hz, 4H), 3.45 – 3.35 (m, 4H), 3.16 (s, 18H), 1.87 (tt, *J* = 15.3, 7.5 Hz, 8H), 1.68 – 1.59 (m, 4H), 1.49 (dt, *J* = 15.0, 7.5 Hz, 4H).

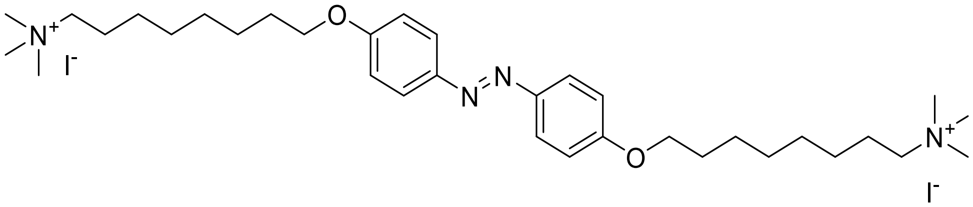

(*E*)-8,8'-((diazene-1,2-diylbis(4,1-phenylene))bis(oxy))bis(*N*,*N*,*N*-trimethyloctan-1-aminium) di-iodide (15d): Yellow solid; yield 99%. ^{1}H NMR (400 MHz, MeOD) δ: 8.21 (t, *J* = 9.1 Hz, 4H), 7.08 (d, *J* = 9.0 Hz, 4H), 4.12 (t, *J* = 6.4 Hz, 4H), 3.35 (dd, *J* = 15.6, 6.9 Hz, 4H), 3.15 (s, 4H), 1.88 – 1.76 (m, 8H), 1.57 – 1.37 (m, 16H).

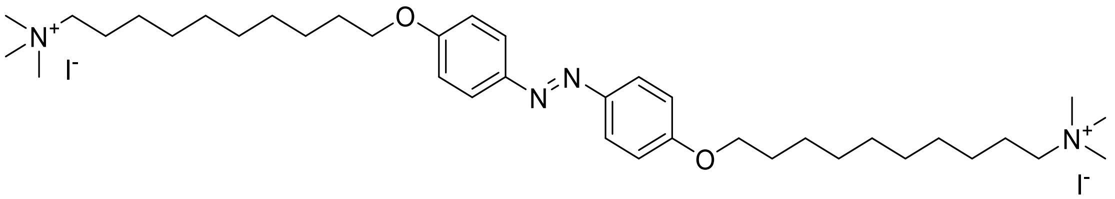

(*E*)-10,10'-((diazene-1,2-diylbis(4,1-phenylene))bis(oxy))bis(*N*,*N*,*N*-trimethyldecan-1-aminium) di-iodide (15e): Off-white solid; yield 99%. ^{1}H NMR (400 MHz, MeOD) δ8.21 (d, *J* = 8.7 Hz, 4H), 7.07 (d, *J* = 8.8 Hz, 4H), 4.12 (t, *J* = 6.4 Hz, 4H), 3.40 – 3.31 (m, 4H), 3.14 (s, 18H), 1.86 – 1.79 (m, 8H), 1.55 – 1.46 (m, 4H), 1.46 – 1.35 (m, 20H).

2. Photoisomerization studies

The rate of isomerization was assessed through a comprehensive analysis employing UV spectroscopy and ^{1}H-NMR photoswitching experiments. These investigations were conducted with the aid of UV light, which effectively facilitated the transition from *trans*-azobenzene to *cis*-azobenzene. Various durations of UV irradiation, followed by relaxation periods, were meticulously examined. Moreover, distinct solvent systems, encompassing aqueous, acetonitrile, methanol, and TSB media, were assessed. Presented herein are the UV and NMR spectra for selected compounds, offering insights into the isomerization process.

Light Irradiation Experiment – ^{1}H NMR Analysis

The photoisomerization behaviour of representative azobenzene derivatives **10b** and **10f** was evaluated using ^{1}H NMR spectroscopy. Each compound (5 mg) was dissolved in 600 µL of DMSO-d_6 and transferred to a standard NMR tube. The tubes were then positioned beneath a 365 nm UV LED light source and wrapped in aluminium foil to eliminate exposure to ambient light and prevent cross-illumination from external sources.

An initial ^{1}H NMR spectrum was recorded at time zero (prior to light exposure). The samples were subsequently irradiated with blue LED light for 3 hours at 37 °C under continued foil shielding. After irradiation, the second ^{1}H NMR spectrum was recorded (3 h time point). The tubes were then incubated in the dark at 37 °C, and further spectra were acquired at 6 h and 24 h. In total, four time points were assessed: 0 h, 3 h (post-irradiation), 6 h, and 24 h. Notably, light exposure occurred only during the initial 3-hour period, while all subsequent measurements were conducted under dark conditions at 37 °C—consistent with the conditions used in follow-up microbiological studies.

Azobenzene derivatives undergo reversible *trans* → *cis* photoisomerization upon exposure to UV light, typically at 365 nm, while thermal energy or visible light (430–550 nm) promotes the reverse *cis* → *trans* isomerisation. The structural changes associated with isomerisation were monitored by ^{1}H NMR, where *trans* and *cis* isomers typically display adjacent resonances for eq. protons, with the trans form generally appearing more downfield and exhibiting larger coupling constants. The *trans*/*cis* ratio was quantified by integration of the corresponding aromatic proton signals.

For compound **10b**, clear upfield shifts in the aromatic region were observed after UV exposure, consistent with *trans*-to-*cis* isomerisation. Specifically, doublets at δ 7.88 and 7.76 ppm, assigned to *ortho* protons of the azobenzene moiety, shifted upfield following irradiation. A similar shift was observed for the signal at δ 7.39 ppm. Over the subsequent dark incubation period, partial thermal relaxation to the *trans* form occurred, although full reversion was not observed within 24 hours.

At time zero, compound **10b** existed majority in the *trans* configuration (98%). After 3 h of irradiation, the *trans*/*cis* ratio shifted to 8% / 91%. Following 6 h in the dark, the ratio changed to 37% / 62%, and by 24 h, it further reverted to 62% / 37%, indicating progressive thermal re-isomerisation. These data are presented in Figure S1. Compound **10f** displayed a similar photoisomerization and relaxation profile, with minor variations in isomer ratios, as illustrated in Figure S2. In both cases, the *cis* isomer predominated in the early post-irradiation phase.

It is worth noting that, due to the experimental setup, the NMR tubes were briefly exposed to ambient

light—particularly during handling and placement in the spectrometer. These short periods of light exposure may have contributed to an accelerated *cis*-to-*trans* photorelaxation, slightly influencing the observed isomer ratios.

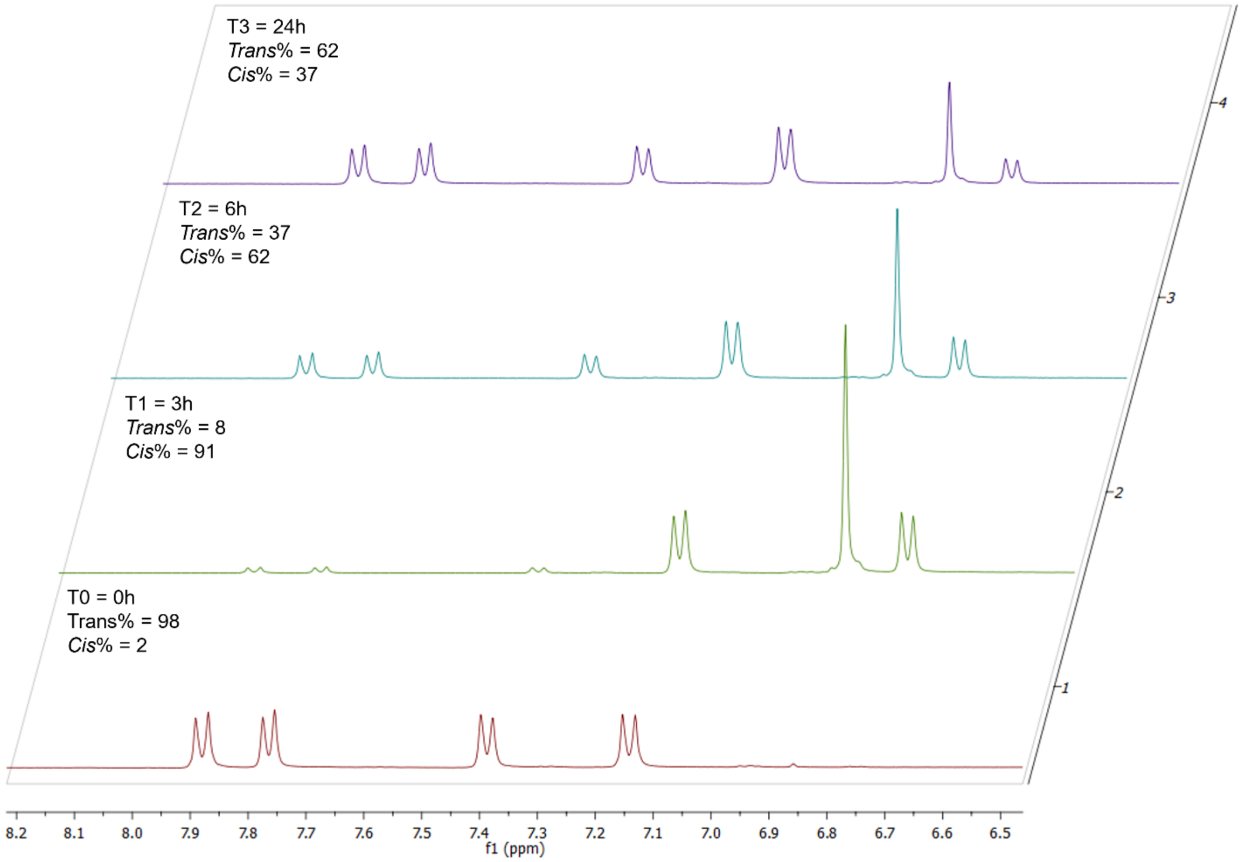

Figure S1. Change in *trans* and *cis* isomeric ratio of compound **10b** over time.

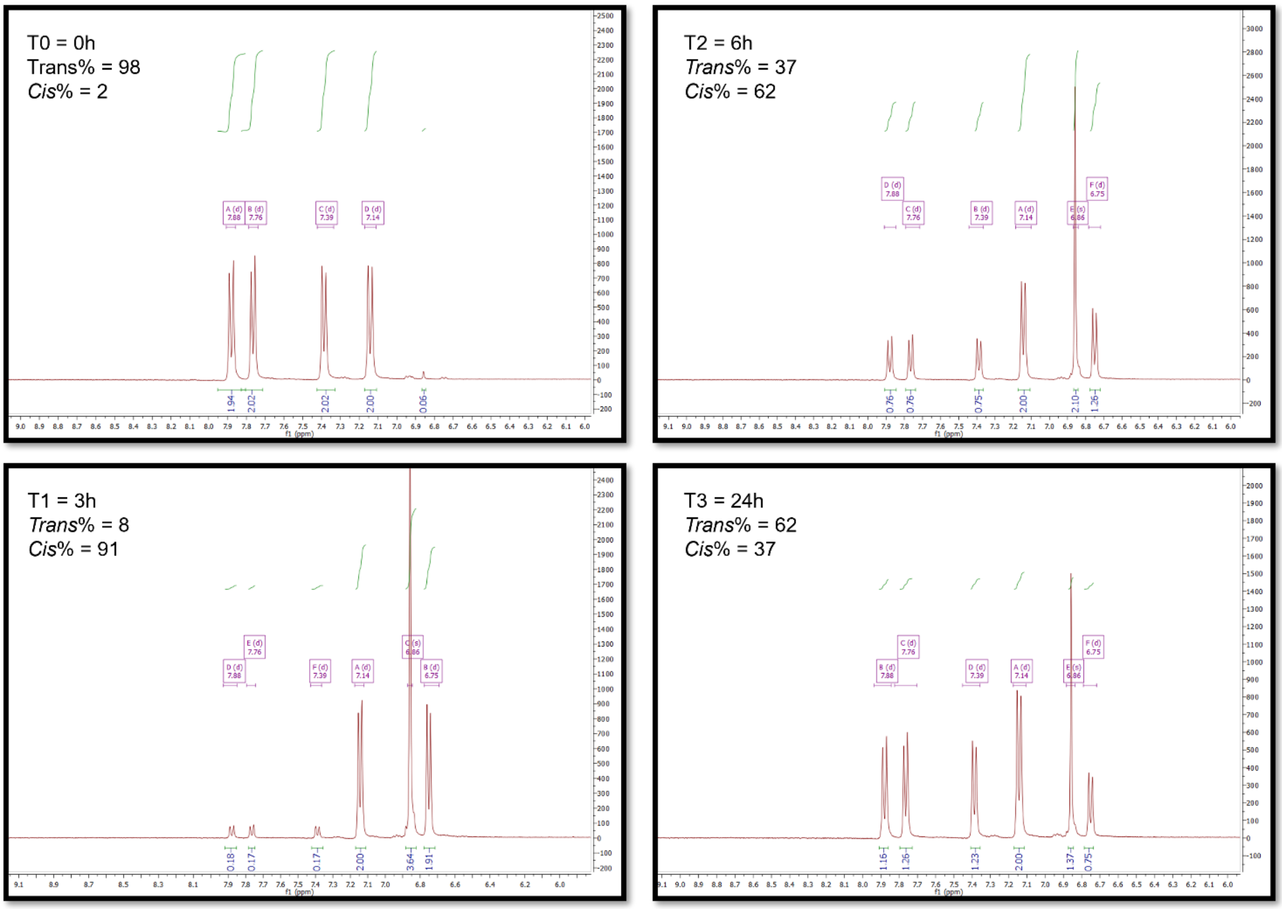

Figure S2. ^{1}H NMR expansion of compound **10b** in the different time points.

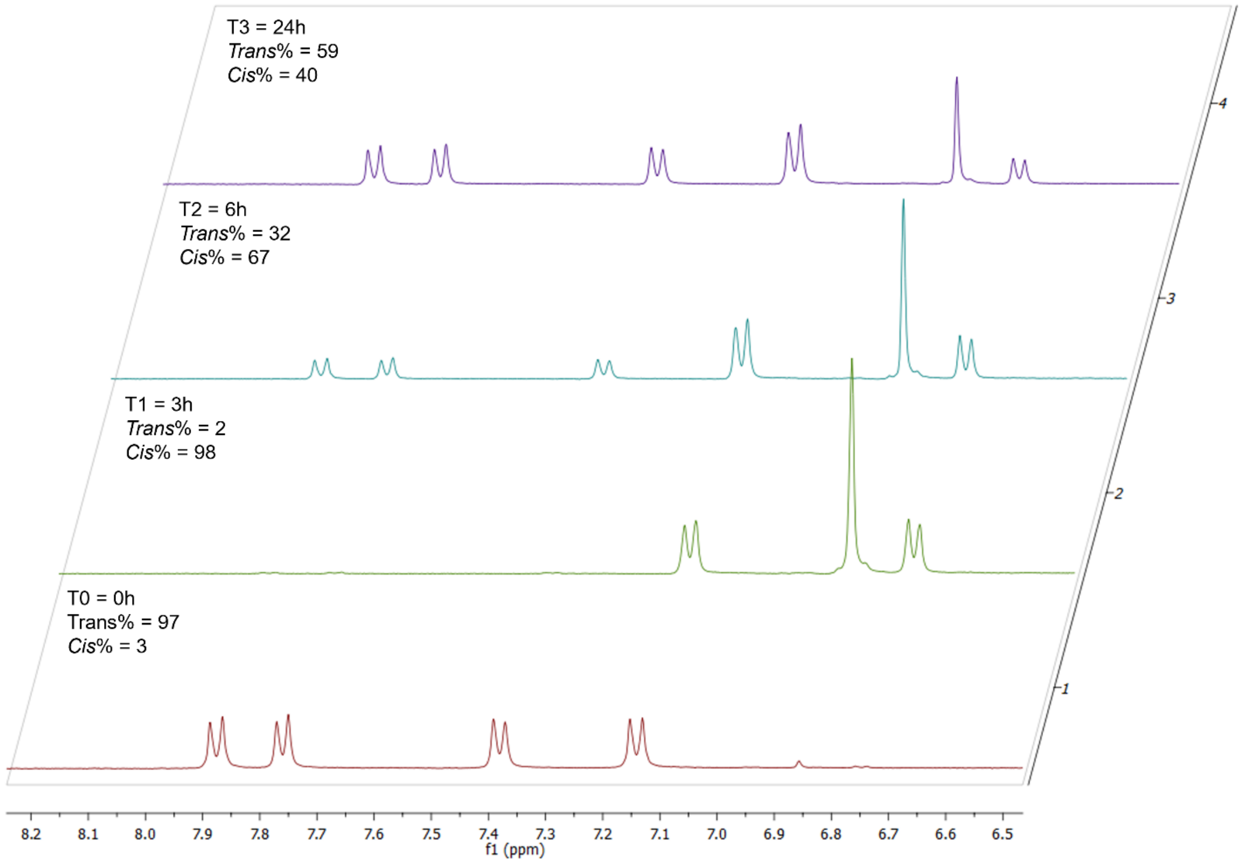

Figure S3. Change in *trans* and *cis* isomeric ratio of compound **10f** over time.

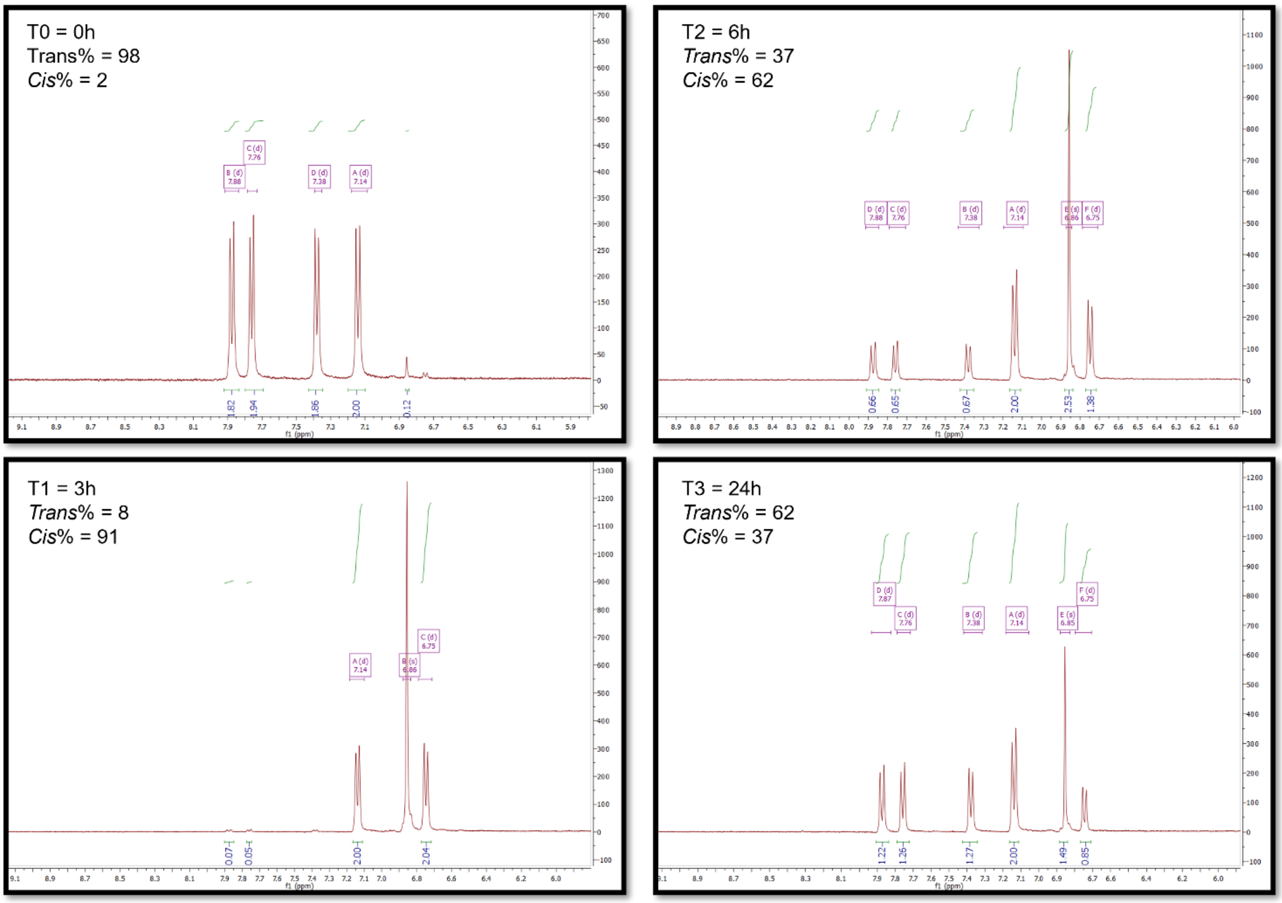

Figure S3b. ^{1}H NMR expansion of compound **10f** in the different time points.

Light Irradiation Experiment – UV Spectroscopy Analysis

The photoisomerization behaviour of representative azobenzene derivatives **10b** and **10f** was further evaluated using UV–vis spectroscopy (Figures S4-S5). Each compound (1 mg) was dissolved in 10 mL of acetonitrile, and the same experimental conditions used for the NMR experiments were applied here. UV–vis spectra were first recorded at time zero, followed by irradiation with 365 nm UV light for 3 hours under dark-adapted conditions at 37 °C. Spectra were subsequently acquired at 3 h (post-irradiation), and additional time points were collected at 6 h, 8 h, and 24 h while the samples remained in the dark. These conditions were chosen to replicate the environment of the forthcoming antibacterial assays.

The UV–vis spectra showed characteristic bands associated with azobenzene isomerisation: a strong $\pi\rightarrow\pi^*$ transition around 350 nm, indicative of the *trans* isomer, and a weaker $n\rightarrow\pi^*$ transition near 440 nm, corresponding to the *cis* isomer. These spectral features confirmed the expected *trans*-to-*cis* photoisomerization and partial thermal relaxation over time.

In addition, photoisomerization studies were performed in tryptic soy broth (TSB), the bacterial growth medium used for subsequent microbiological assays. Representative azobenzene derivatives (**10b** and **10f**) were dissolved in TSB, and UV–vis spectra were acquired under identical experimental conditions (Figure S6-S7). Notably, the spectra in TSB closely resembled those obtained in acetonitrile, displaying the same key transition bands, thereby confirming that the azobenzenes retain

their photochemical responsiveness under biologically relevant conditions.

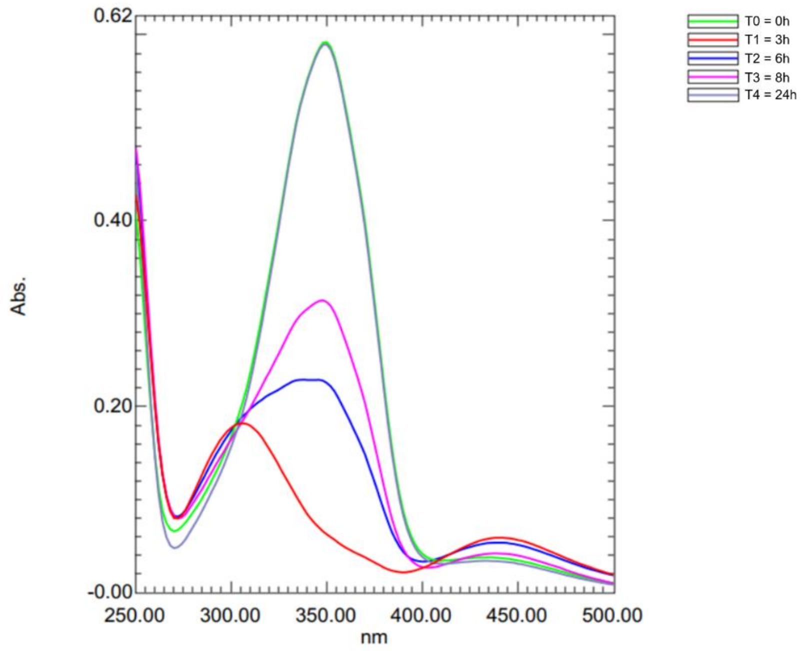

Figure S4. UV spectrum of compound **10b** in acetonitrile.

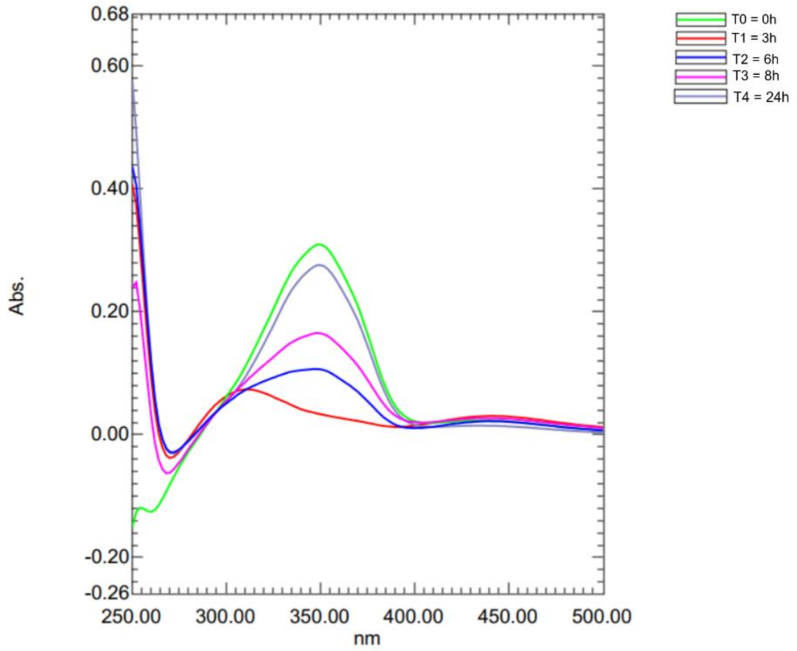

Figure S5. UV spectrum of compound **10f** in acetonitrile.

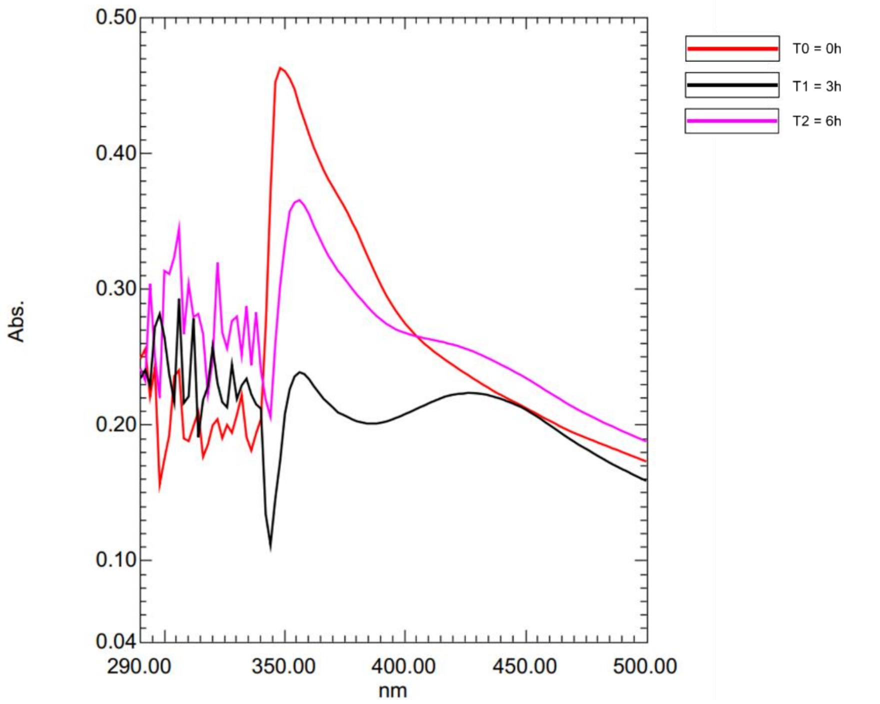

Figure S6. UV spectrum of compound **10b** in TSB medium.

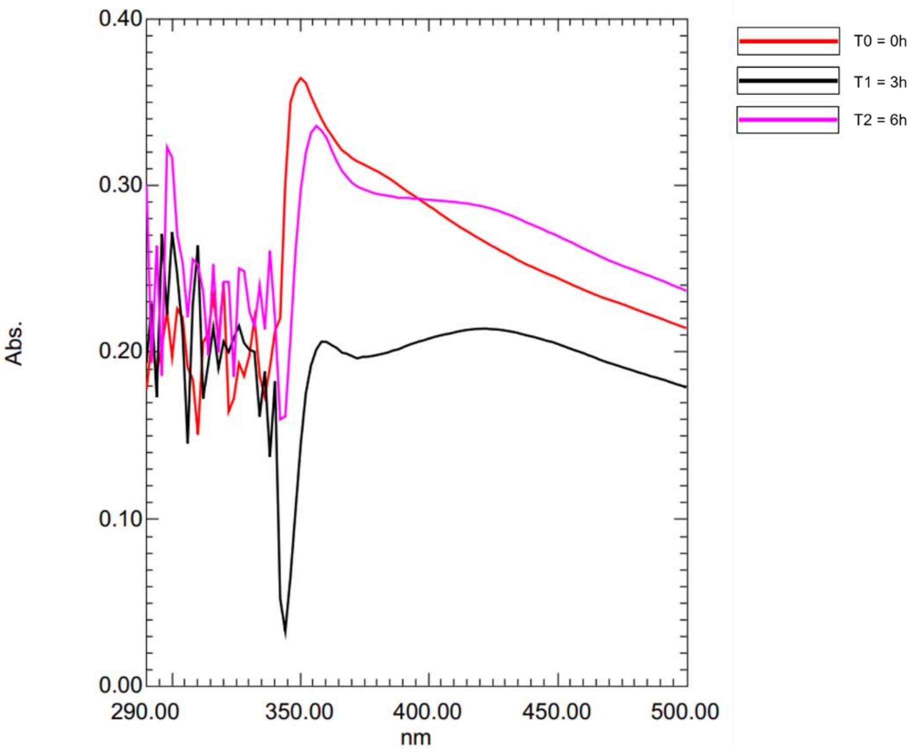

Figure S7. UV spectrum of compound **10f** in TSB medium.

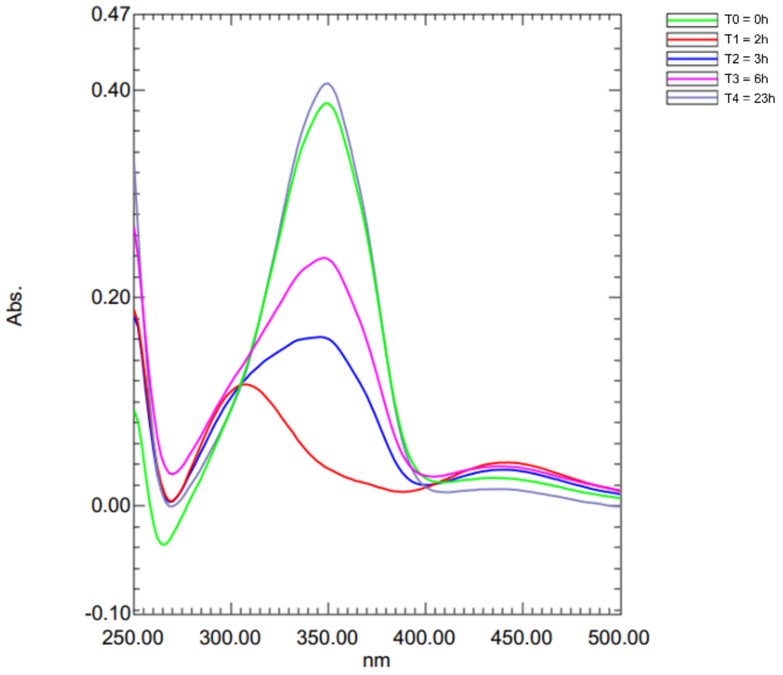

Figure S8. UV spectrum of compound **10c** in acetonitrile.

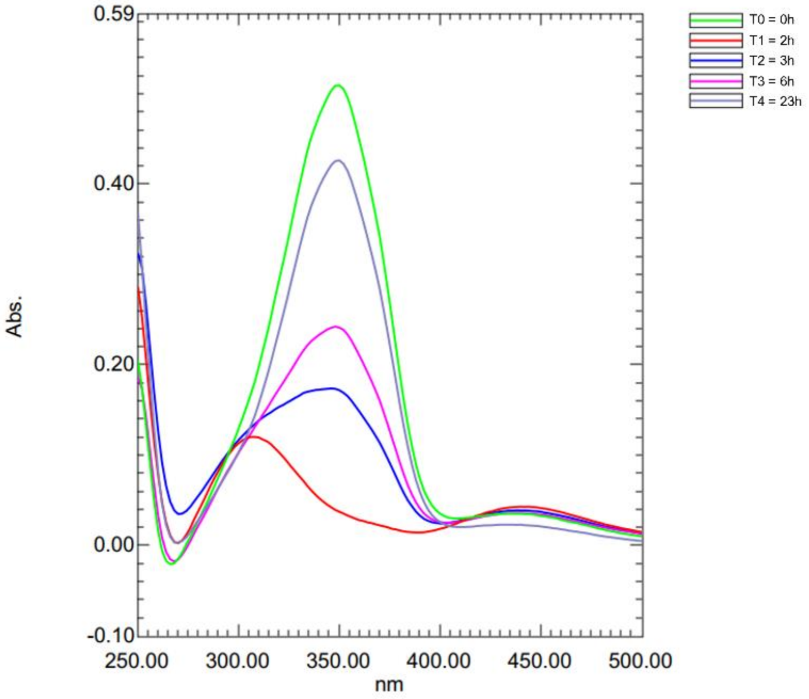

Figure S9. UV spectrum of compound **10d** in acetonitrile.

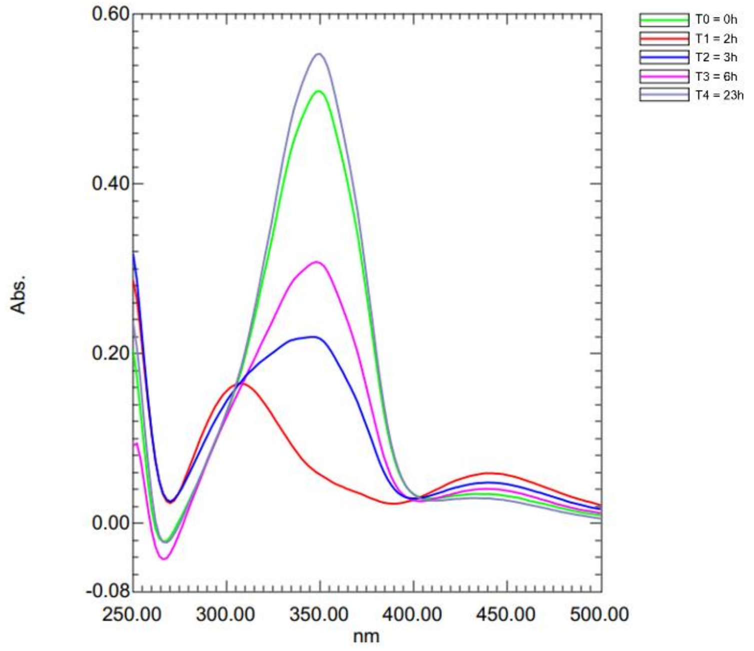

Figure S10. UV spectrum of compound **10e** in acetonitrile.

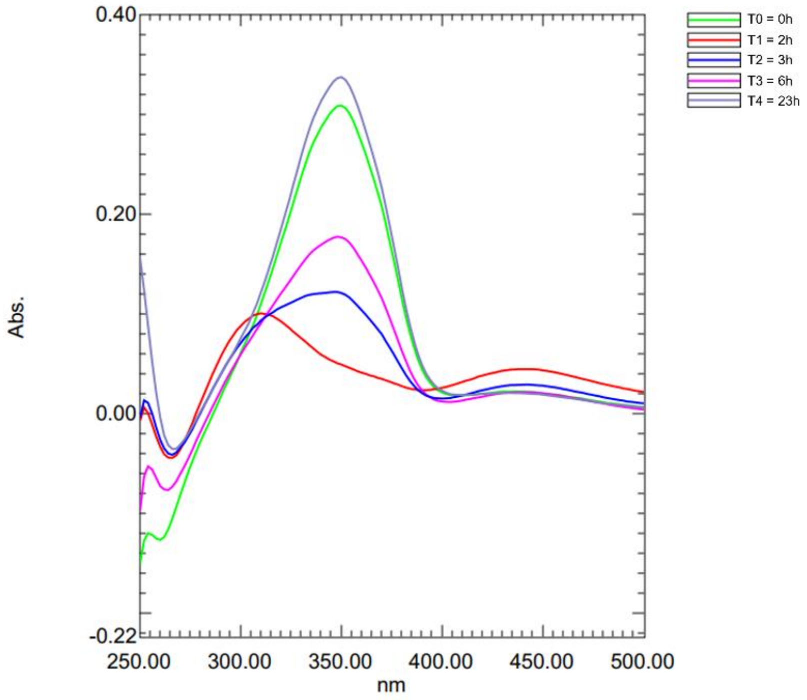

Figure S11. UV spectrum of compound **10g** in acetonitrile.

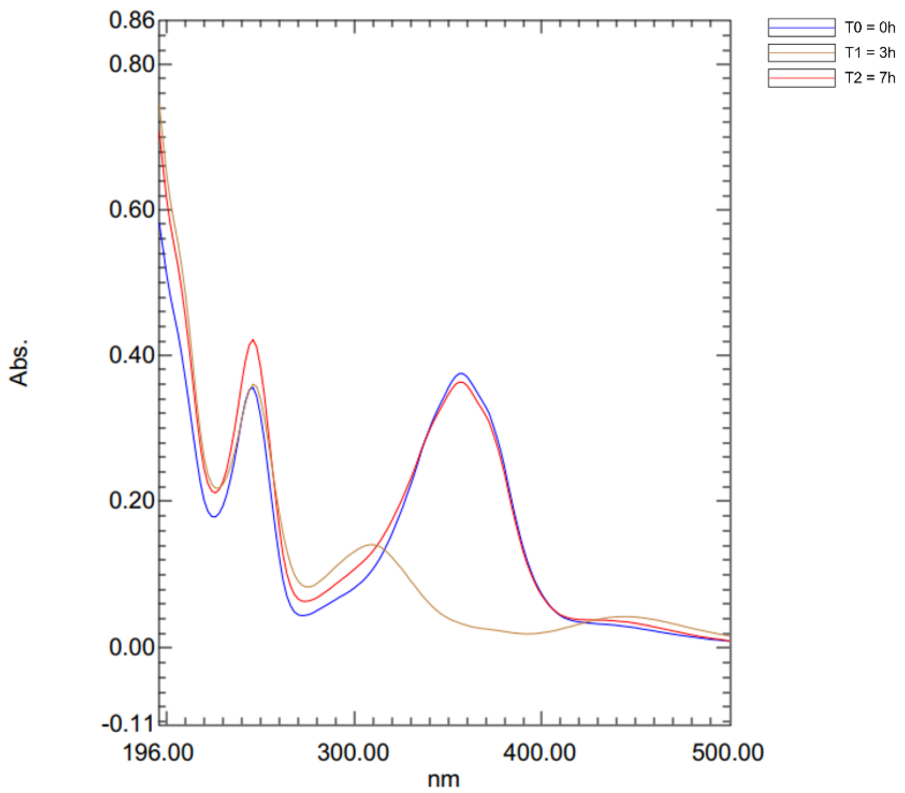

Figure S12. UV spectrum of compound **10h** in acetonitrile.

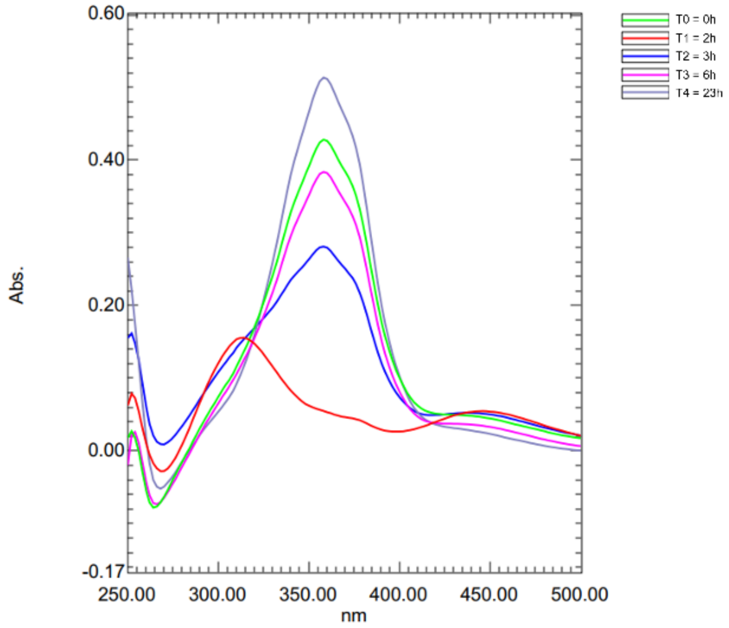

Figure S13. UV spectrum of compound **10i** in acetonitrile.

3. Microbiology

The experimental setup for the microbiological studies closely mirrored that of the photoisomerization experiments. Multiple conditions were tested to evaluate the potential influence of UV light (365 nm) on bacterial growth in the absence of photoswitchable compounds, serving as control experiments. These conditions included: (1) continuous UV irradiation during the entire 24-hour incubation period; (2) UV irradiation limited to the first 2 hours of incubation; (3) alternating on/off UV exposure in 2-hour intervals throughout the 24-hour period; and (4) pre-irradiation of compound stock solutions in DMSO prior to their addition to the plates.

All experiments were conducted at 37 °C under dark-adapted conditions, with the plates covered in aluminium foil to prevent exposure to ambient light. Importantly, none of the UV irradiation conditions tested had a detectable impact on bacterial growth, thereby validating the use of UV light as a tool to manipulate the photoisomeric state of the photoswitchable compounds without confounding microbial viability.

Based on these findings, the following standardized protocol was implemented for compound treatment studies: bacterial cultures were plated together with the photoswitchable compounds (initially in their *trans*-enriched photostationary state), followed immediately by UV irradiation at 365 nm for 2 hours at 37 °C in the dark. The UV light was then switched off, and incubation continued for an additional 22 hours in the dark, ensuring no further photoactivation or exposure to natural light. This setup aimed to replicate the *trans*/*cis* distribution profile observed in the NMR and UV photoisomerization studies, with compounds being converted predominantly to the *cis* form during the initial UV exposure and gradually thermally relaxing back to the *trans* form over time.

This protocol was designed to probe whether the *cis*–*trans* interconversion of the photoswitchable compounds could influence bacterial viability by disrupting membrane integrity, particularly during the critical early stages of growth. Within the first 8 hours post-inoculation—encompassing the lag and exponential growth phases—the compounds predominantly exist in the *cis* configuration due to prior UV activation. As incubation proceeds in the dark, the *cis* isomers undergo gradual thermal relaxation back to the more stable *trans* form. This dynamic transition is hypothesised to generate molecular-level mechanical perturbations that may alter membrane organisation or integrity, thereby impacting bacterial fitness. Additionally, these structural fluctuations could indirectly influence other cellular processes that are particularly vulnerable to membrane dynamics.

Notably, experiments employing intermittent UV exposure (i.e., light switched on and off every 2 hours) did not alter the antibacterial activity of the tested azobenzenes. Conversely, continuous irradiation throughout the 24-hour incubation occasionally affected compound activity, suggesting potential photodegradation or other light-induced effects. Therefore, the 4-hour UV activation window was selected as the optimal condition for maintaining biological relevance while preserving compound integrity.

Minimum Inhibitory Concentration (MIC) assay

MICs were determined using a standard microbroth dilution method in 96-well plates against a panel of multidrug-resistant Gram-positive and Gram-negative bacterial strains. The panel included *Staphylococcus aureus* ATCC 9144 (MSSA), *S. aureus* NCTC 13616 (MRSA), *S. aureus* NCTC

8325 (USA300), *S. aureus* SA1199B, *Klebsiella pneumoniae* NCTC 13368 (ESBL), *K. pneumoniae* M6, *Acinetobacter baumannii* AYE (ATCC BAA-1710), *A. baumannii* ATCC 17978, *Pseudomonas aeruginosa* PAO1, *P. aeruginosa* NCTC 13437, and *Escherichia coli* NCTC 12923. All assays were conducted in Tryptic Soy Broth (TSB).
Test compounds were dissolved in DMSO to prepare stock solutions and subsequently diluted in TSB to generate a 2-fold serial dilution across wells (100 µL per well). Overnight bacterial cultures grown in TSB were diluted to an OD_{600} of 0.01 and added to each well (100 µL), yielding a final assay volume of 200 µL per well. The resulting concentration range for compounds spanned from 0.0625 to 128 µg/mL.
Immediately following compound treatment, all plates were irradiated with 365 nm UV light (365 nm) for 2 hours at 37 °C in a dark-adapted chamber. Plates were covered with aluminium foil throughout the experiment to prevent exposure to ambient light and to ensure uniform light exposure. After 2 hours of irradiation, the UV light was switched off, and plates were further incubated in the dark at 37 °C for an additional 22 hours.
To assess the effect of photoisomerization on antibacterial activity, a parallel control plate was prepared identically but maintained in the dark without UV exposure. This allowed comparison between bacteria treated with compounds in their photoinduced *cis*-rich state versus those exposed to the thermodynamically stable *trans* configuration.
Optical density (OD_{600}) was measured after 24 hours using a Fluorostar plate reader. The MIC was defined as the lowest compound concentration that prevented visible bacterial growth, corresponding to $OD_{600} < 0.1$. Control wells containing DMSO at eq. concentrations exhibited no bacterial growth inhibition.

Table S1. Light control of antibacterial activity in photoswitchable amphiphilic lipids (PALs) against Gram-Negative bacteria

Entry	Cmpd	Gram Negative MIC (µg/mL)													
		K. pneumoniae				***A. baumannii***				***P. aeruginosa***				***E. coli***	
		13368		***M6***		***AYE***		***17978***		***PAO1***		***13437***		***12923***	
		LOFF	LON	LOFF	LON	LOFF	LON	LOFF	LON	LOFF	LON	LOFF	LON	LOFF	LON
1	**10a**	>128	>128	>128	>128	16-32	64	8-16	32	>128	>128	>128	>128	32	>128
2	**10b**	>64	>64	>64	>64	8	16	8	64	>64	>64	>64	>64	>64	>64
3	**10c**	>64	>64	>64	>64	4	16	16	32	>64	>64	>64	>64	>64	>64
4	**10d**	>64	>64	>64	>64	8	>64	>64	16	>64	>64	>64	>64	>64	>64
5	**10e**	>64	>64	>64	>64	>64	>64	>64	>64	>64	>64	>64	>64	>64	>64
6	**10f**	>64	>64	>64	>64	>64	>64	>64	>64	>64	>64	>64	>64	>64	>64
7	**10g**	>64	>64	>64	>64	>64	>64	>64	>64	>64	>64	>64	>64	>64	>64
8	**10h**	>64	>64	>64	>64	>64	>64	>64	>64	>64	>64	>64	>64	>64	>64
9	**10i**	>128	>128	>128	>128	16-32	64	8-16	32	>128	>128	>128	>128	32	>128
10	**12a**	>64	>64	>64	>64	>64	>64	>64	>64	>64	>64	>64	>64	>64	>64
11	**12b**	>64	>64	>64	>64	>64	>64	>64	>64	>64	>64	>64	>64	>64	>64
12	**12c**	>64	>64	>64	>64	>64	>64	>64	>64	>64	>64	>64	>64	>64	>64

4. Molecular dynamic simulation

Molecular dynamics (MD) simulations were performed using GROMACS 2021 software package[1] and the CHARMM36 all-atom force field[2,3]. The initial bilayer was generated via CHARMM-GUI[4], following the protocol outlined by Manzo et al.[5] to model a Gram-positive bacterial membrane composed of POPG lipids. Each bilayer contained 200 lipids (100 per leaflet).

In all simulations, ten molecules were arranged in a structured grid ~6.8 Å above the membrane surface. To study composition effects, we prepared multiple systems in which the proportion of cis and trans isomers among the ten molecules was varied (0, 10, 50, 90, and 100% *cis* isomers), while the total number of molecules remained constant. These coordinates were used as the initial placement for subsequent molecular dynamics simulations. The system was solvated using TIP3P water molecules, and Na^+ and Cl^- ions were introduced to neutralize the system and achieve a final salt concentration of 0.15 M, approximating physiological conditions.

Energy minimization was conducted at 310 K using the steepest descent algorithm until the maximum force was below 1000 kJ mol^{-1} nm^{-1} (~3000–4000 steps). Equilibration was performed under the NVT ensemble for 100 ps, followed by the NPT ensemble for 1000 ps with position restraints on the compounds. The Nose–Hoover thermostat and semi-isotropic Parrinello–Rahman barostat were employed for temperature and pressure coupling, respectively. All bonds involving hydrogen atoms were constrained using the LINCS algorithm. Final runs were carried out in the NPT ensemble using 2 fs intervals, recording trajectories every 2 ps. All simulations were run for a total of 200ns each.

5. References

1. Lindahl, E.; Abraham, M.; Hess, B.; van der Spoel, D. GROMACS 2021 Manual (Version 2021). Zenodo, 2021. https://doi.org/10.5281/zenodo.4457591.\
2. Best, R. B.; et al. Optimization of the Additive CHARMM All-Atom Protein Force Field Targeting Improved Sampling of the Backbone φ, ψ and Side-Chain χ1 and χ2 Dihedral Angles. J. Chem. Theory Comput. 2012, 8 (9), 3257–3273. https://doi.org/10.1021/ct300400x.
3. Huang, J.; MacKerell, A. D. CHARMM36 All-Atom Additive Protein Force Field: Validation Based on Comparison to NMR Data. J. Comput. Chem. 2013, 34 (25), 2135–2145. https://doi.org/10.1002/jcc.23354.
4. Lee, J.; et al. CHARMM-GUI Input Generator for NAMD, GROMACS, AMBER, OpenMM, and CHARMM/OpenMM Simulations Using the CHARMM36 Additive Force Field. J. Chem. Theory Comput. 2016, 12 (1), 405–413. https://doi.org/10.1021/acs.jctc.5b00935.
5. Manzo, G.; Ferguson, P. M.; Hind, C. K.; Clifford, M.; Gustilo, V. B.; Ali, H.; Bansal, S. S.; Bui, T. T.; Drake, A. F.; Atkinson, R. A.; Sutton, J. M.; Lorenz, C. D.; Phoenix, D. A.; Mason, A. J. Antimicrobial Peptides with Anticancer Properties: Mechanisms of Action and Structure-Activity Relationships. Sci. Rep. 2019, 9, 10934. https://doi.org/10.1038/s41598-019-47327-w.

6. NMR spectra

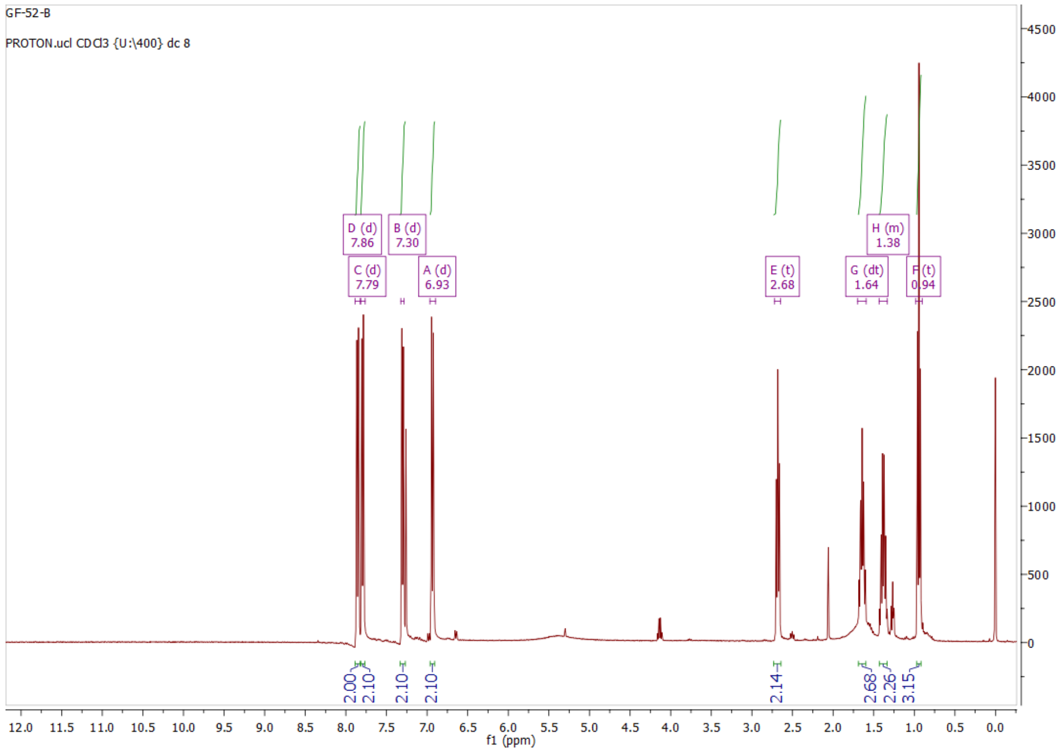

Figure S14. ^{1}H NMR spectrum of compound **8a** (400 MHz, $CDCl_3$)

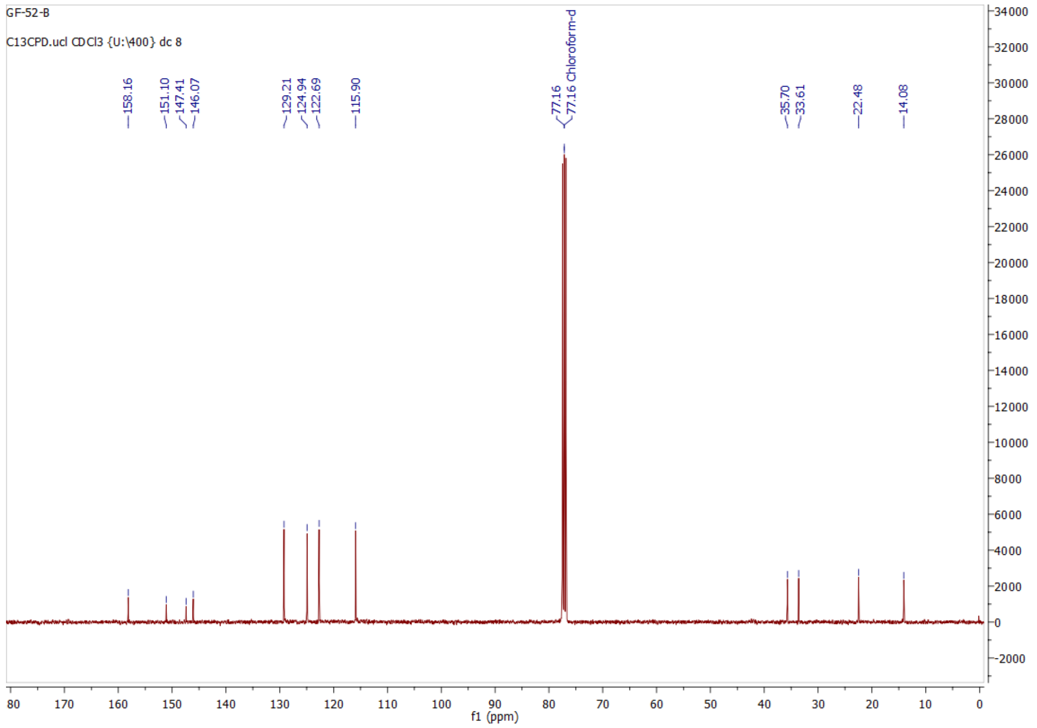

Figure S15. ^{13}C NMR spectrum of compound **8a** (101 MHz, $CDCl_3$)

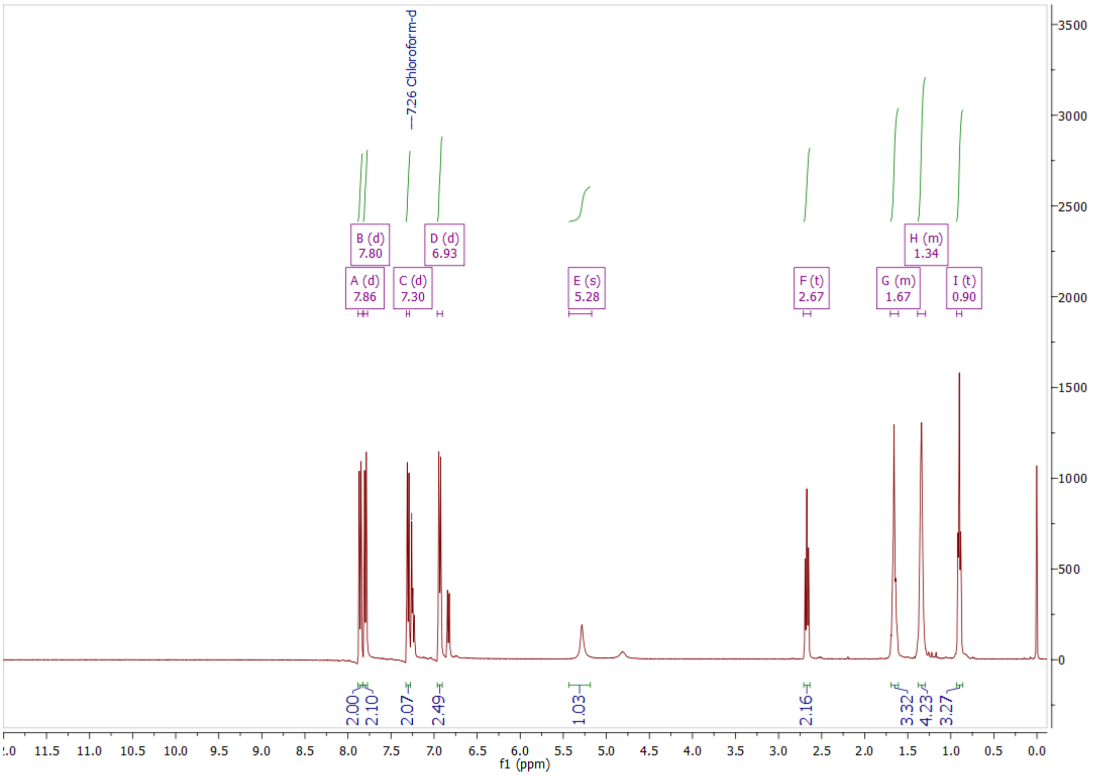

Figure S16. ^{1}H NMR spectrum of compound **8b** (400 MHz, $CDCl_3$)

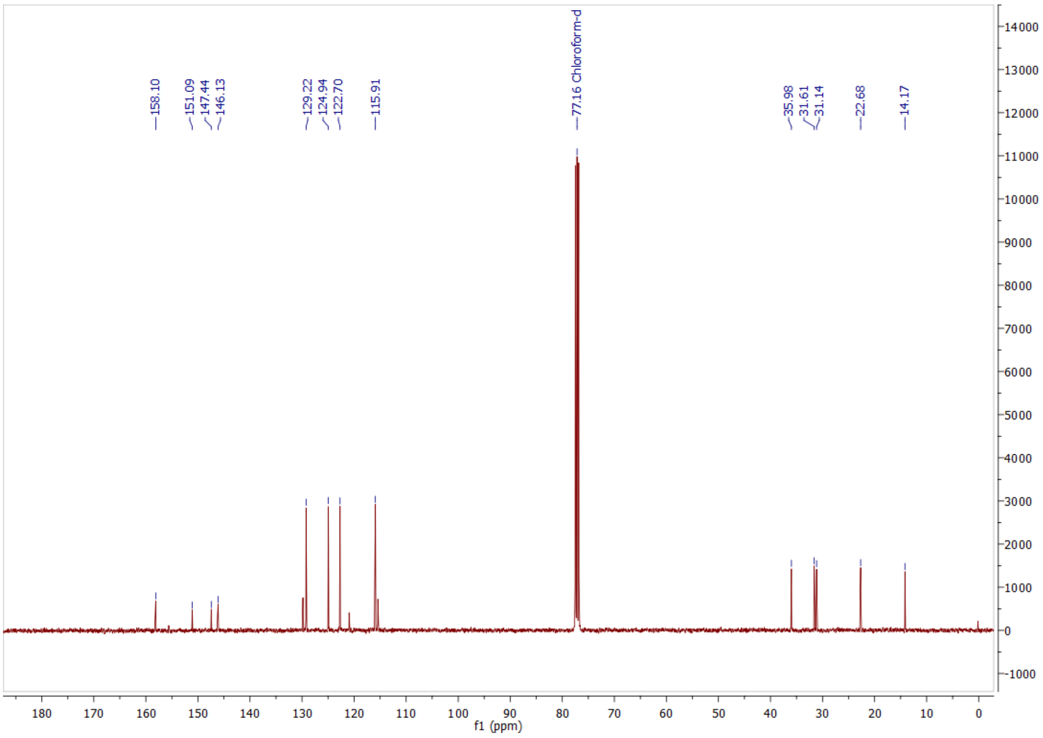

Figure S17. ^{13}C NMR spectrum of compound **8b** (101 MHz, $CDCl_3$)

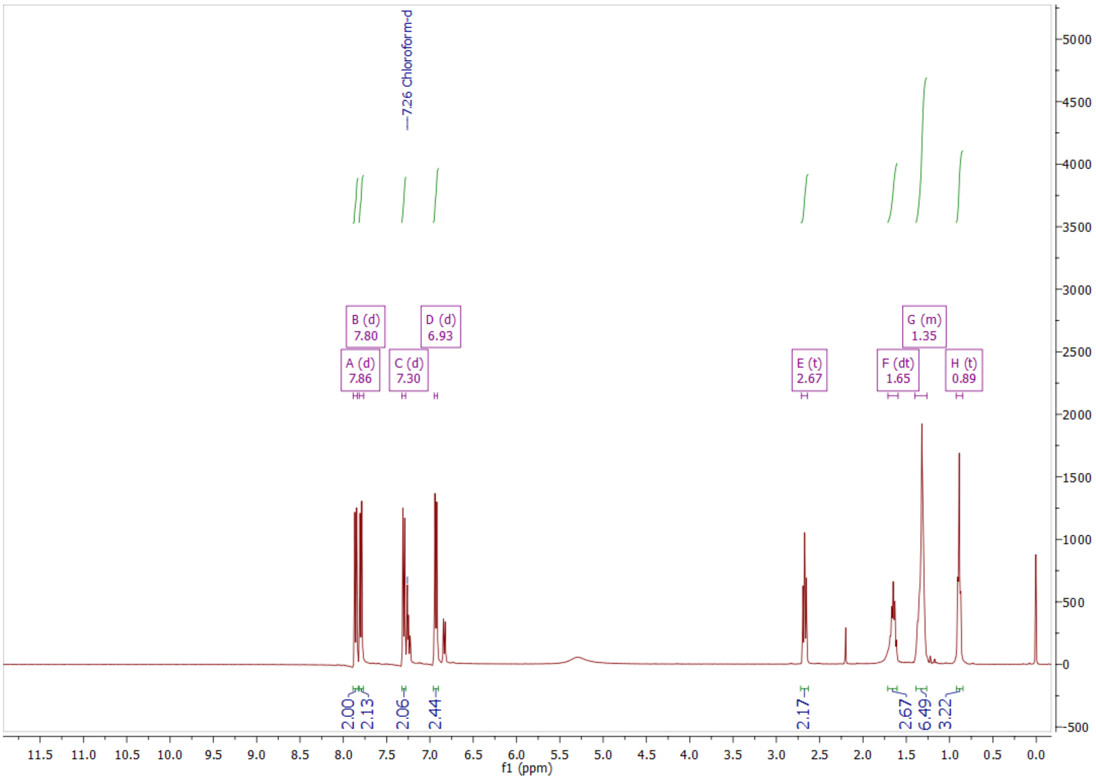

Figure S18. ^{1}H NMR spectrum of compound **8c** (400 MHz, $CDCl_3$)

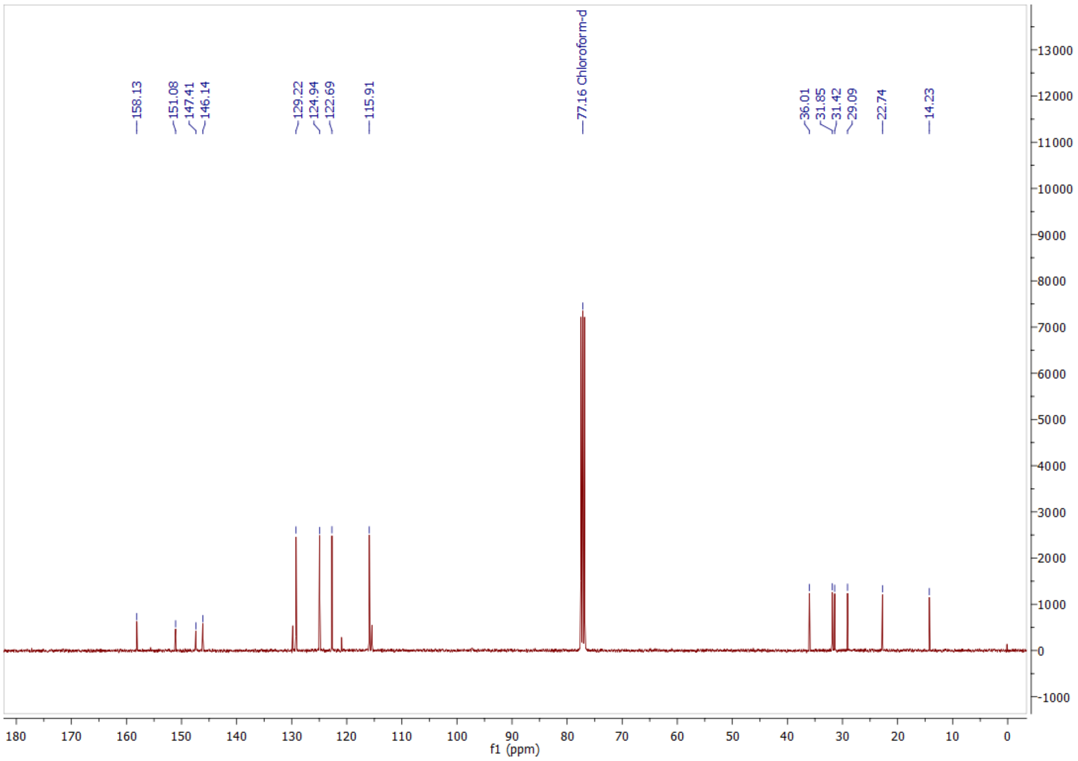

Figure S19. ^{13}C NMR spectrum of compound **8c** (101 MHz, $CDCl_3$)

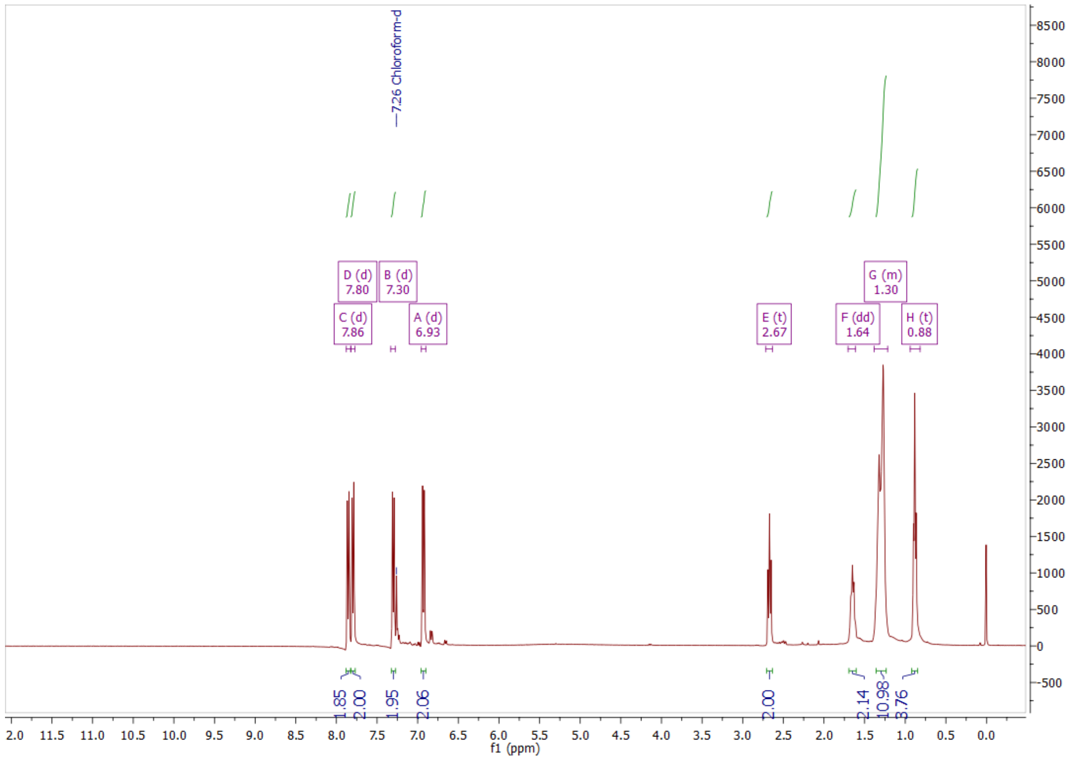

Figure S20. ^{1}H NMR spectrum of compound **8d** (400 MHz, $CDCl_3$)

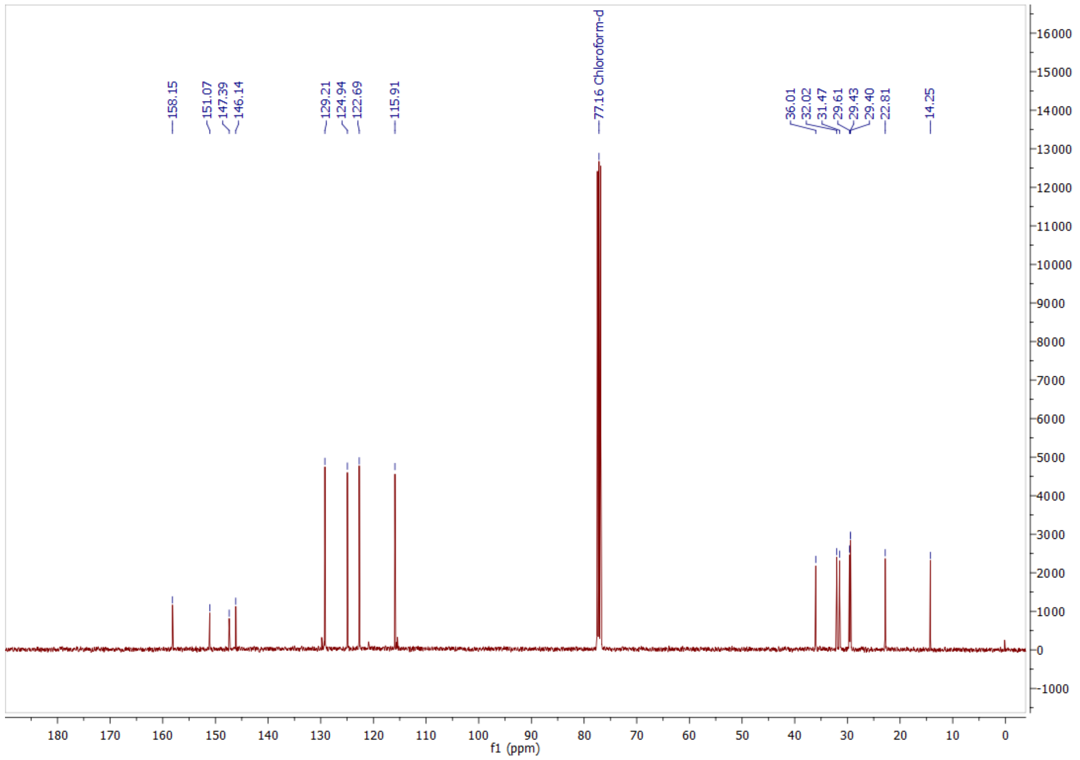

Figure S21. ^{13}C NMR spectrum of compound **8d** (101 MHz, $CDCl_3$)

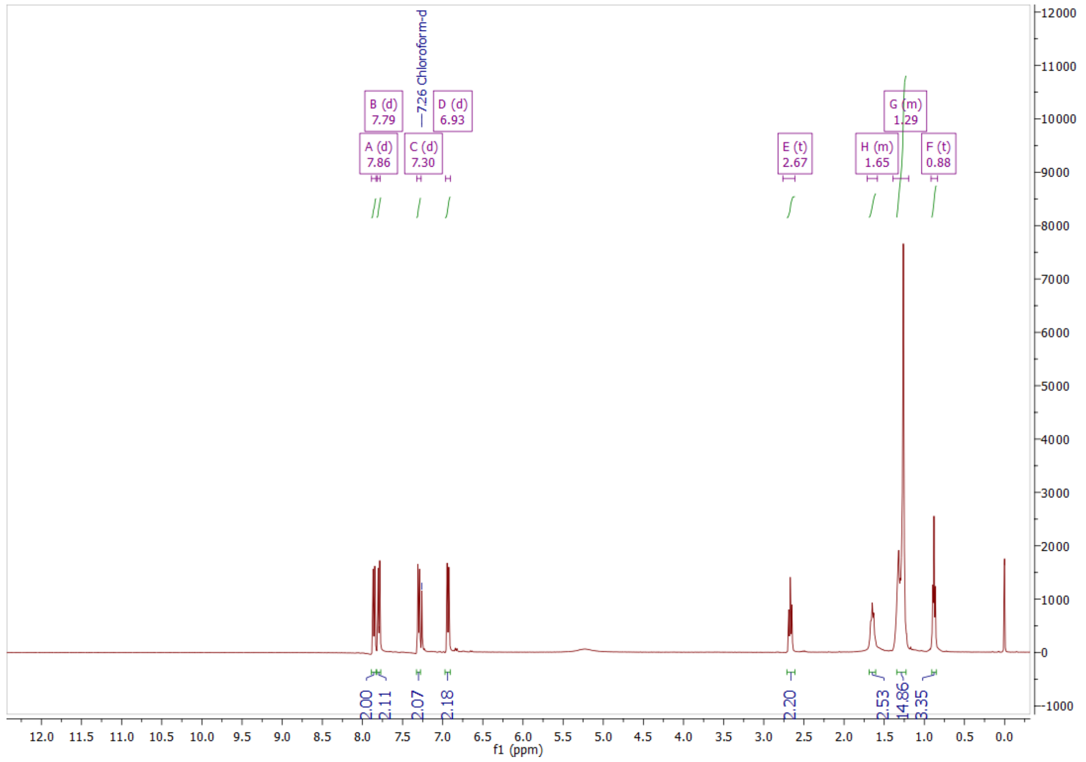

Figure S22. ^{1}H NMR spectrum of compound **8e** (400 MHz, $CDCl_3$)

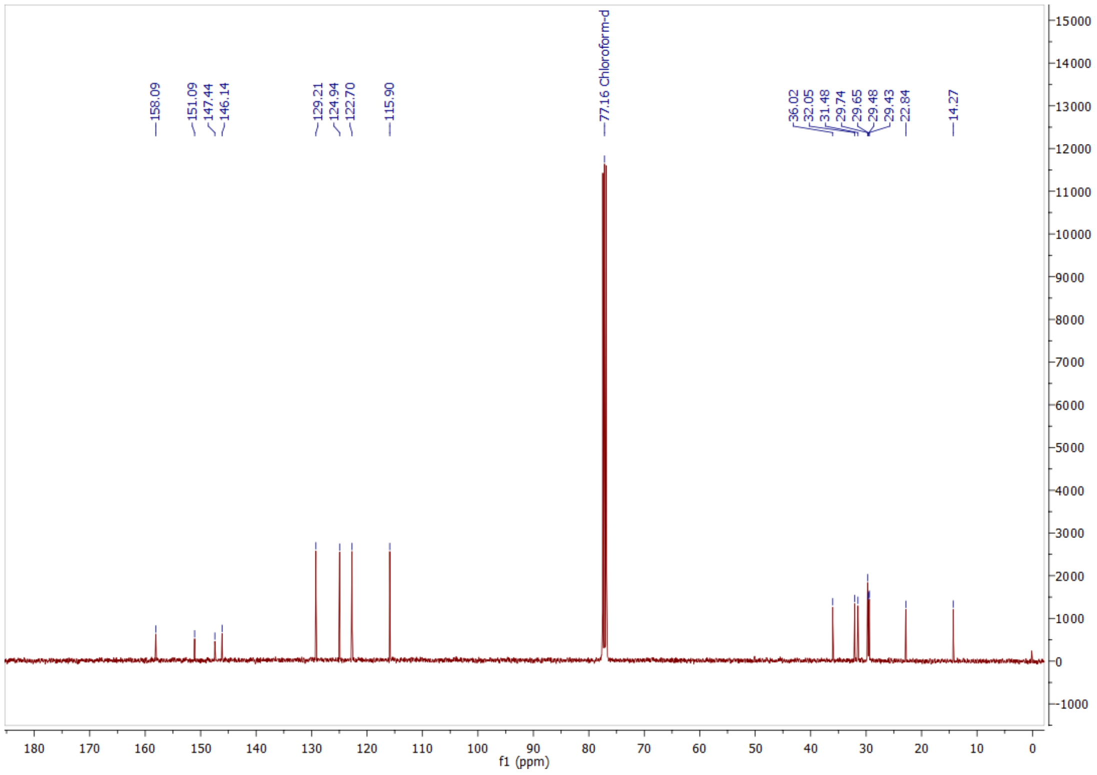

Figure S23. ^{13}C NMR spectrum of compound **8e** (101 MHz, $CDCl_3$)

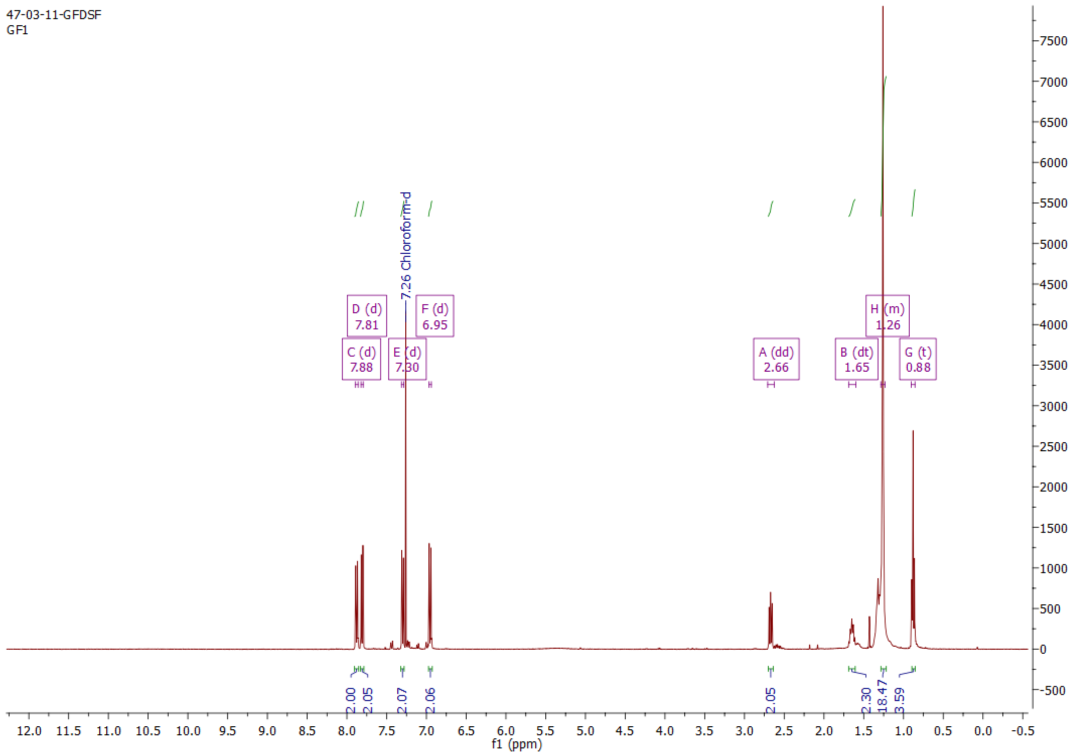

Figure S24. ^{1}H NMR spectrum of compound **8f** (400 MHz, $CDCl_3$)

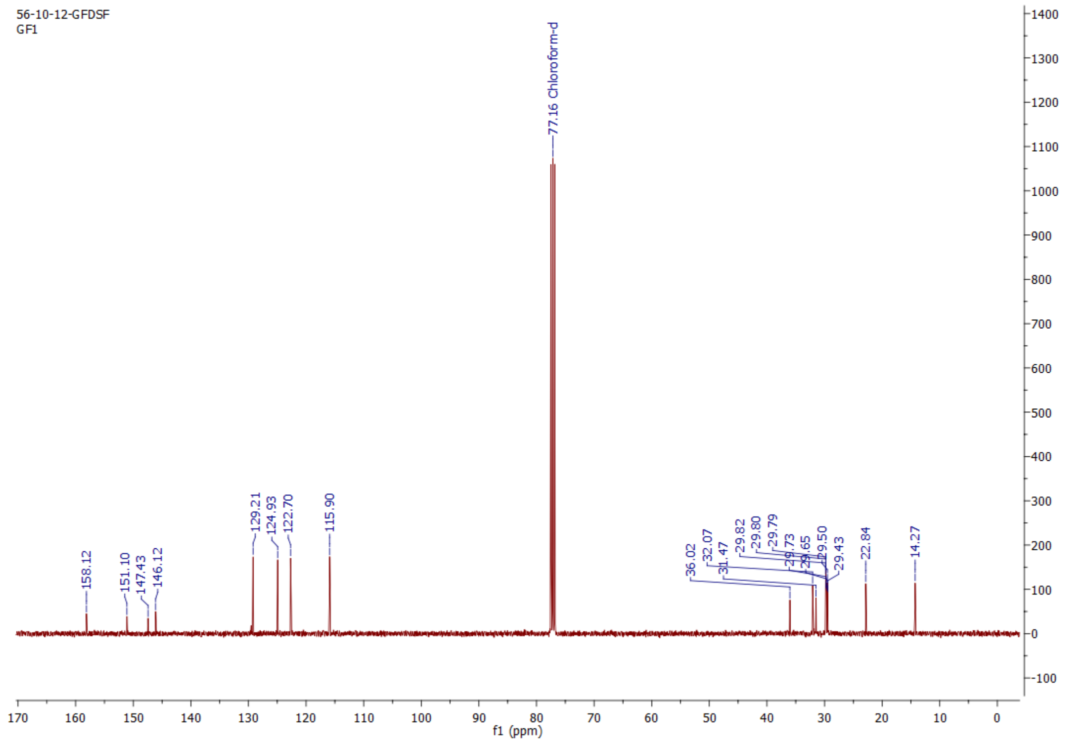

Figure S25. ^{13}C NMR spectrum of compound **8f** (101 MHz, $CDCl_3$)

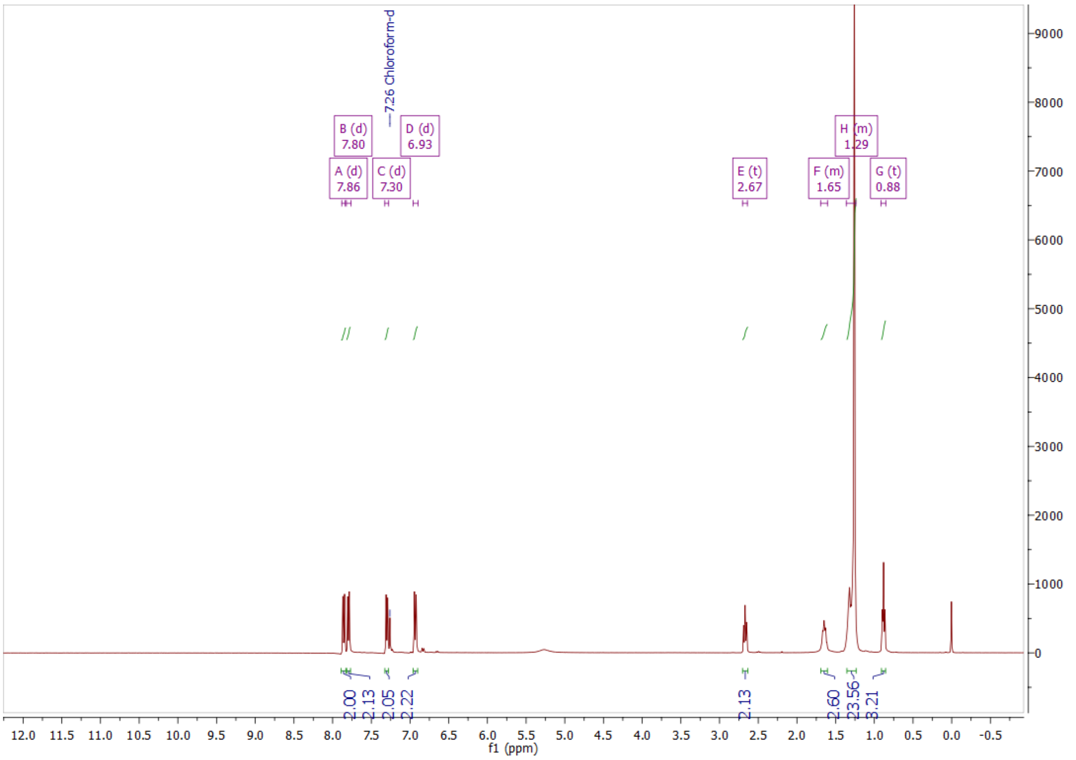

Figure S26. ^{1}H NMR spectrum of compound **8g** (400 MHz, $CDCl_3$)

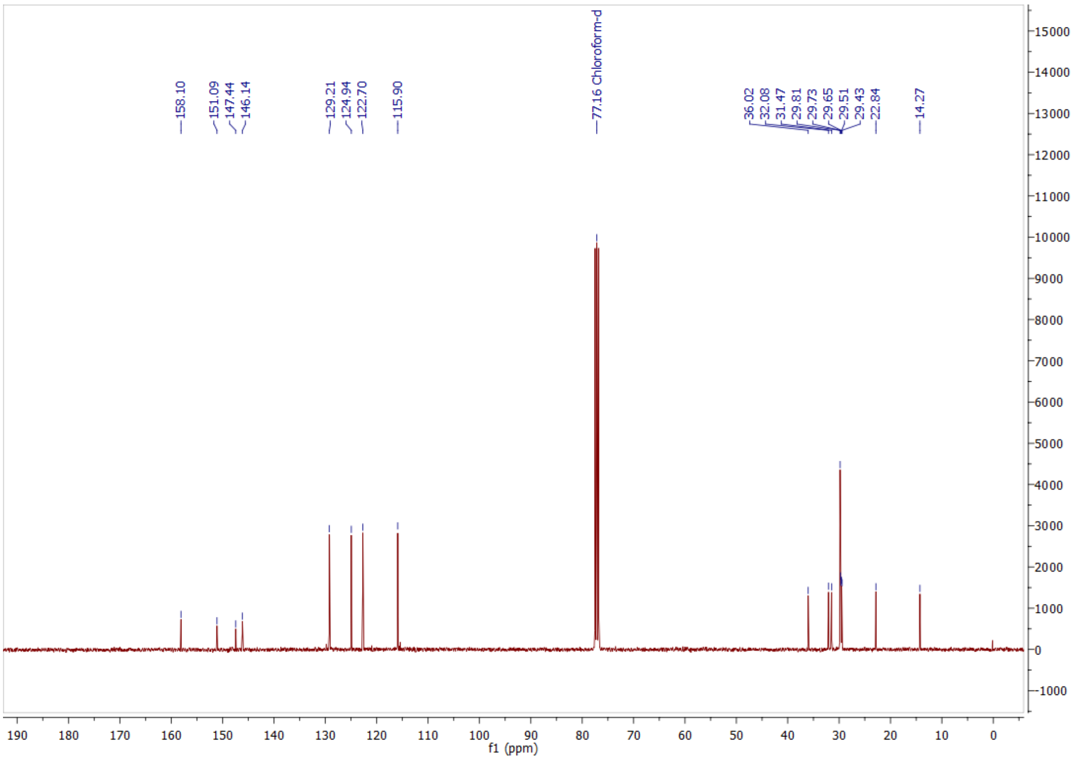

Figure S27. ^{13}C NMR spectrum of compound **8g** (101 MHz, $CDCl_3$)

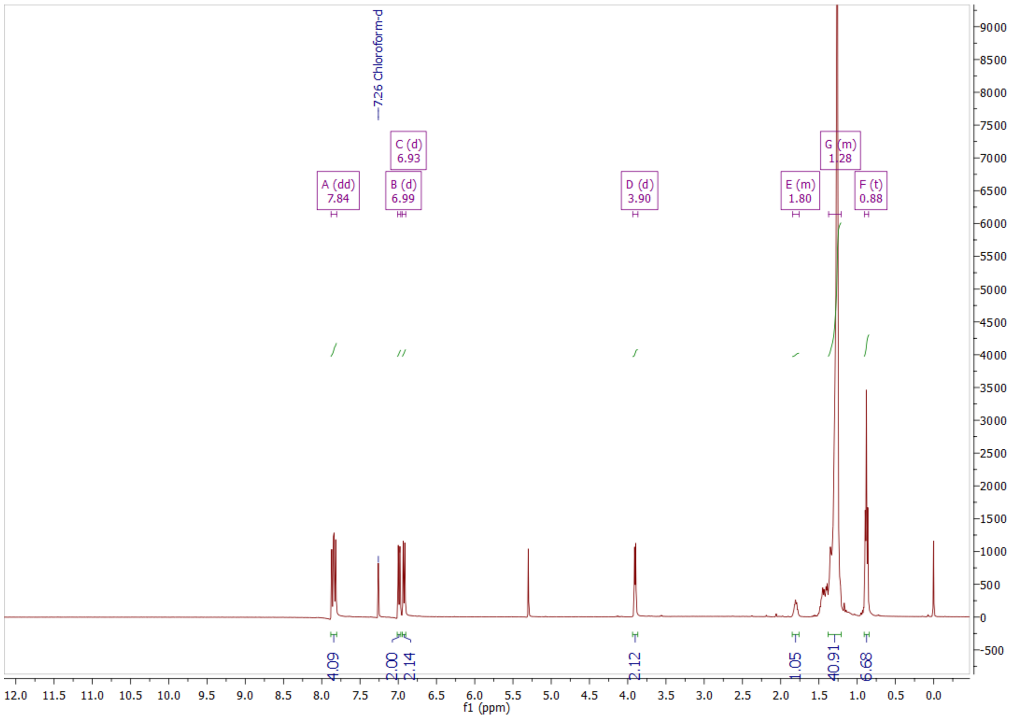

Figure S28. ^{1}H NMR spectrum of compound **8i** (400 MHz, $CDCl_3$)

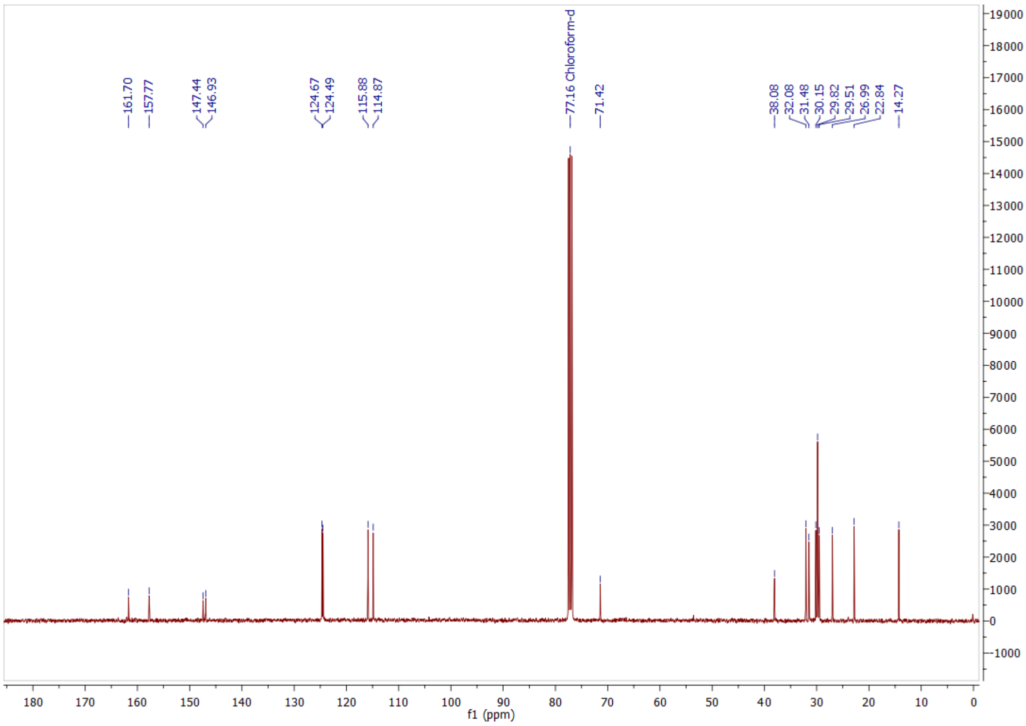

Figure S29. ^{13}C NMR spectrum of compound **8i** (101 MHz, $CDCl_3$)

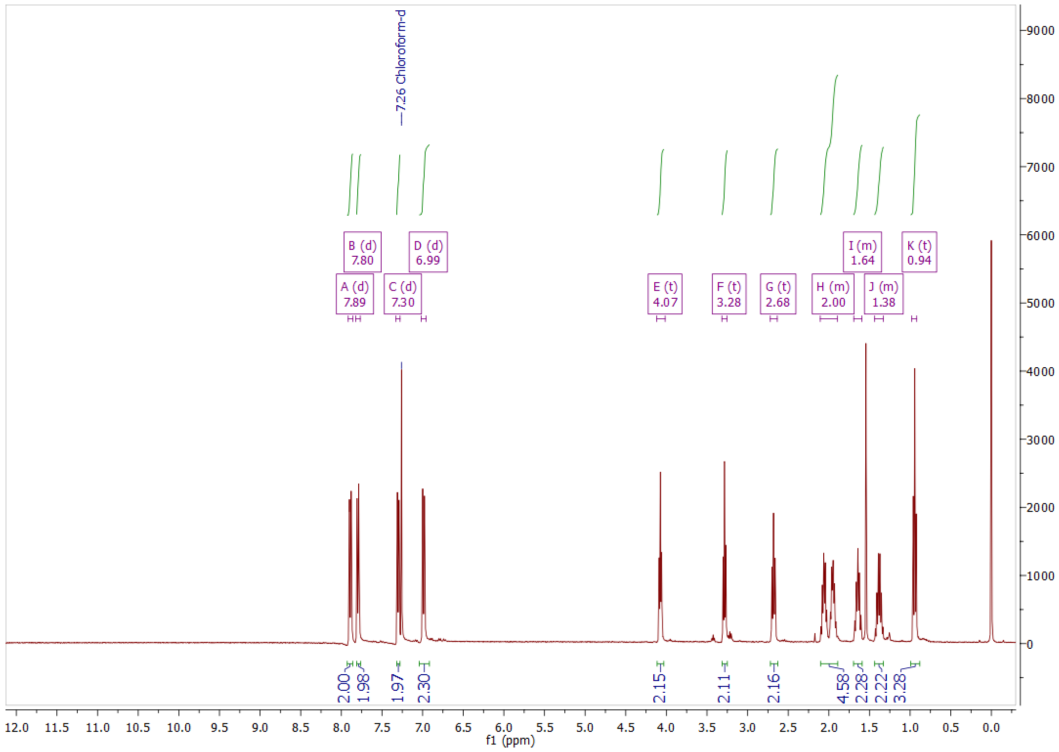

Figure S30. ^{1}H NMR spectrum of compound **9a** (400 MHz, $CDCl_3$)

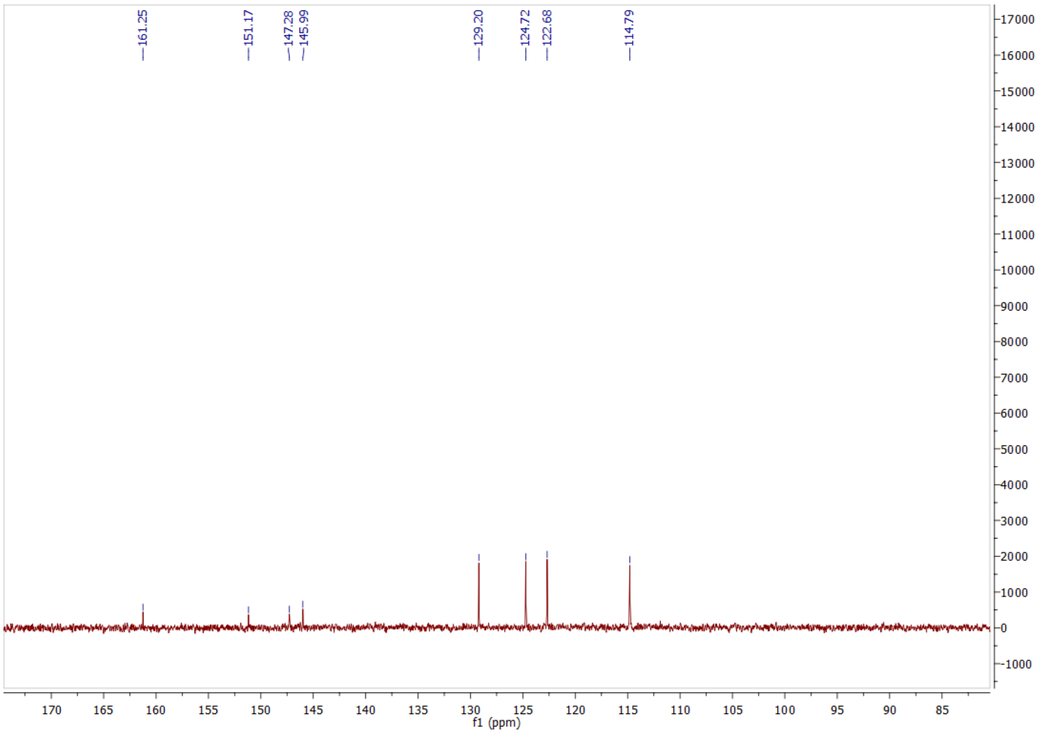

Figure S31. ^{13}C NMR spectrum of compound **9a** (101 MHz, $CDCl_3$)

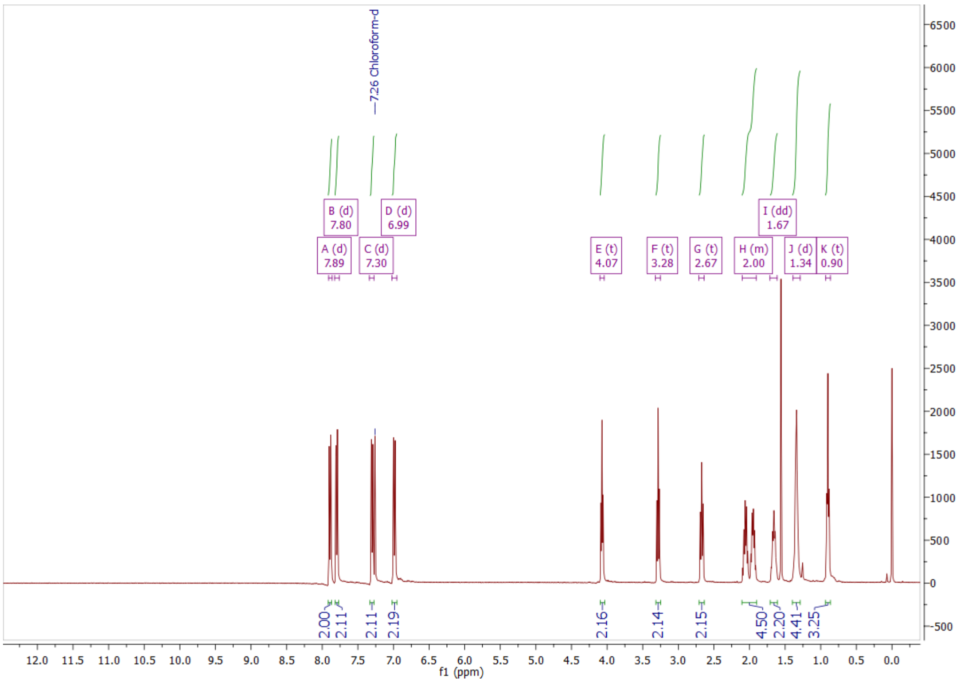

Figure S32. ^{1}H NMR spectrum of compound **9b** (400 MHz, $CDCl_3$)

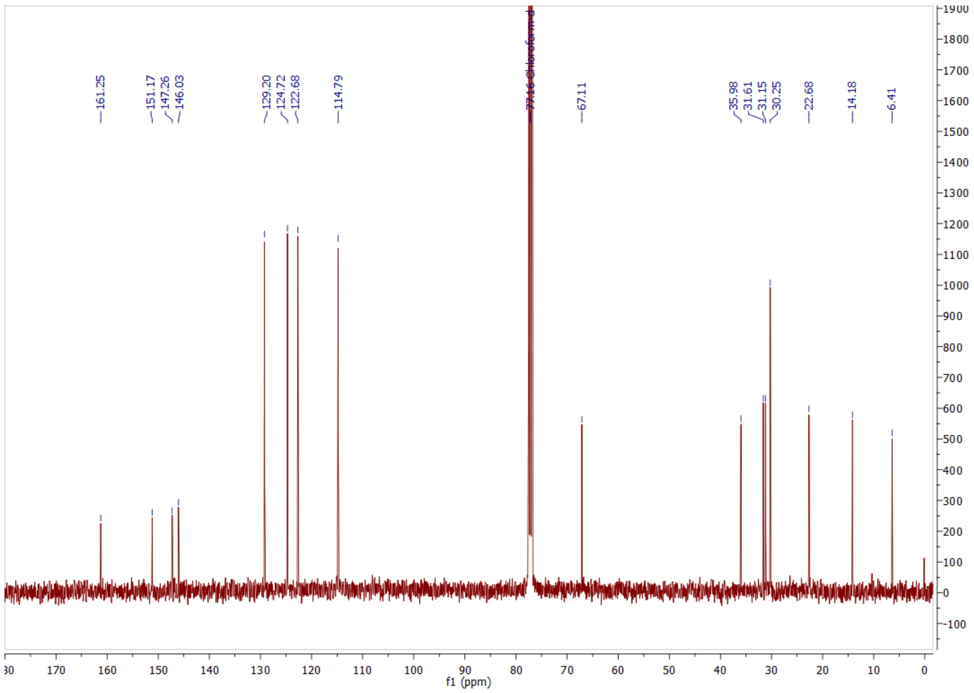

Figure S33. ^{13}C NMR spectrum of compound **9b** (101 MHz, $CDCl_3$)

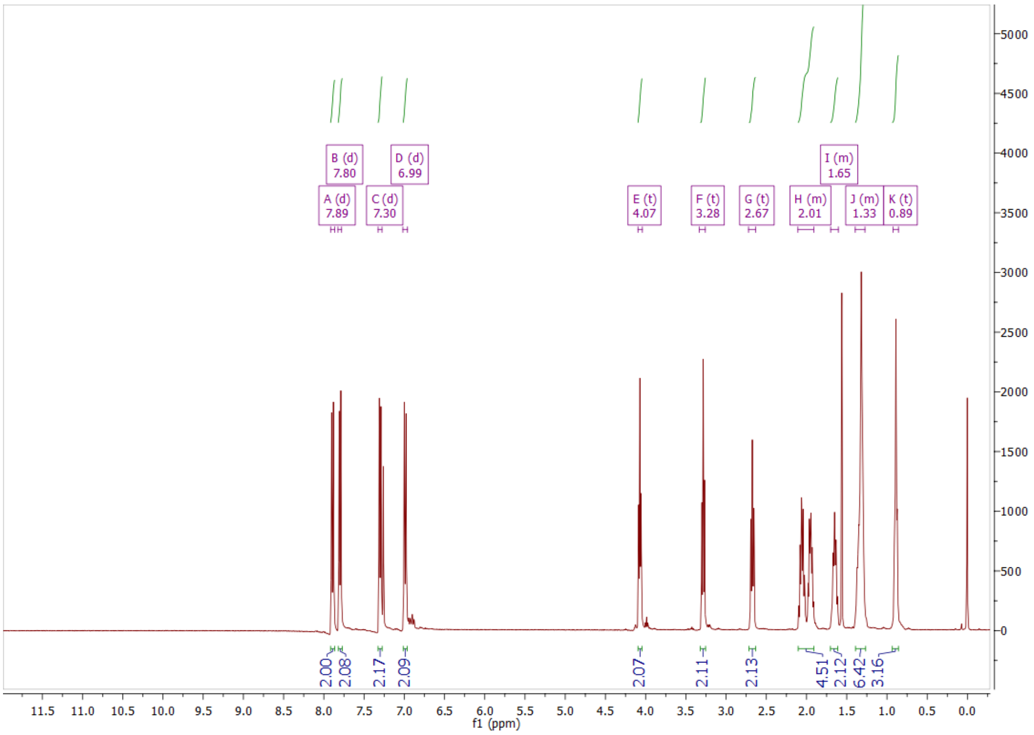

Figure S34. ^{1}H NMR spectrum of compound **9c** (400 MHz, $CDCl_3$)

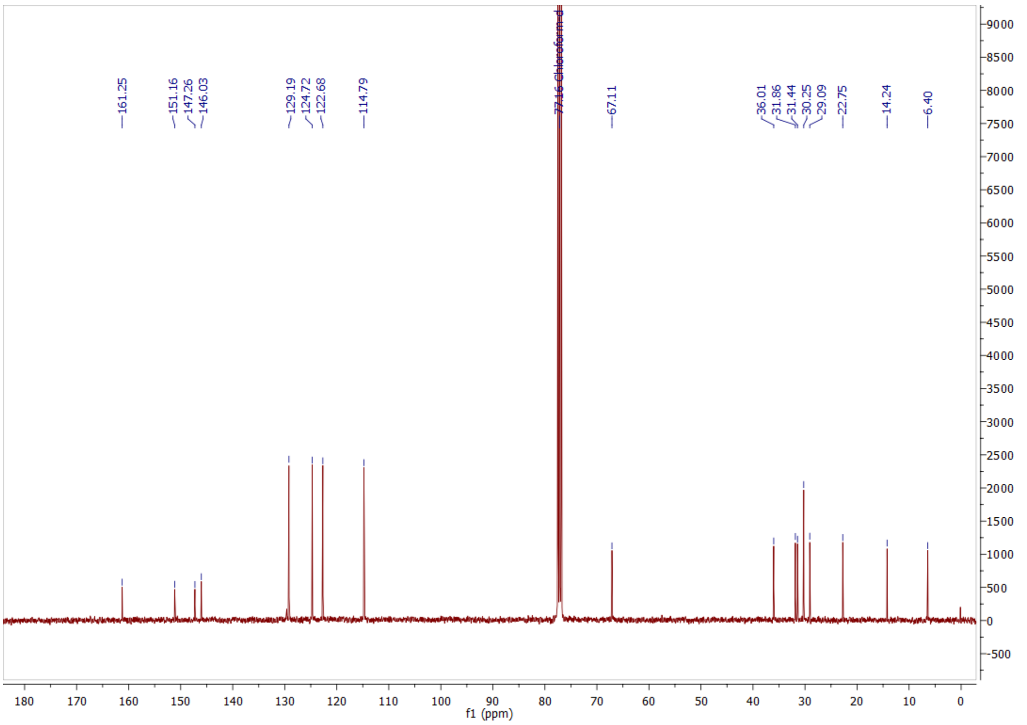

Figure S35. ^{13}C NMR spectrum of compound **9c** (101 MHz, $CDCl_3$)

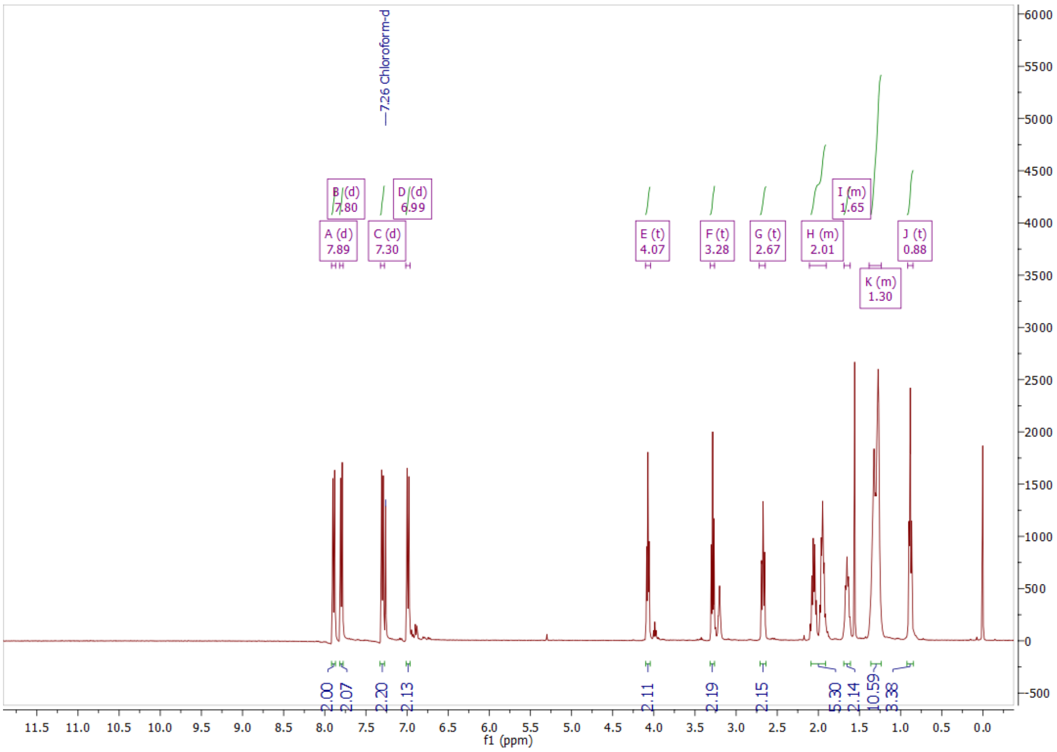

Figure S36. ^{1}H NMR spectrum of compound **9d** (400 MHz, $CDCl_3$)

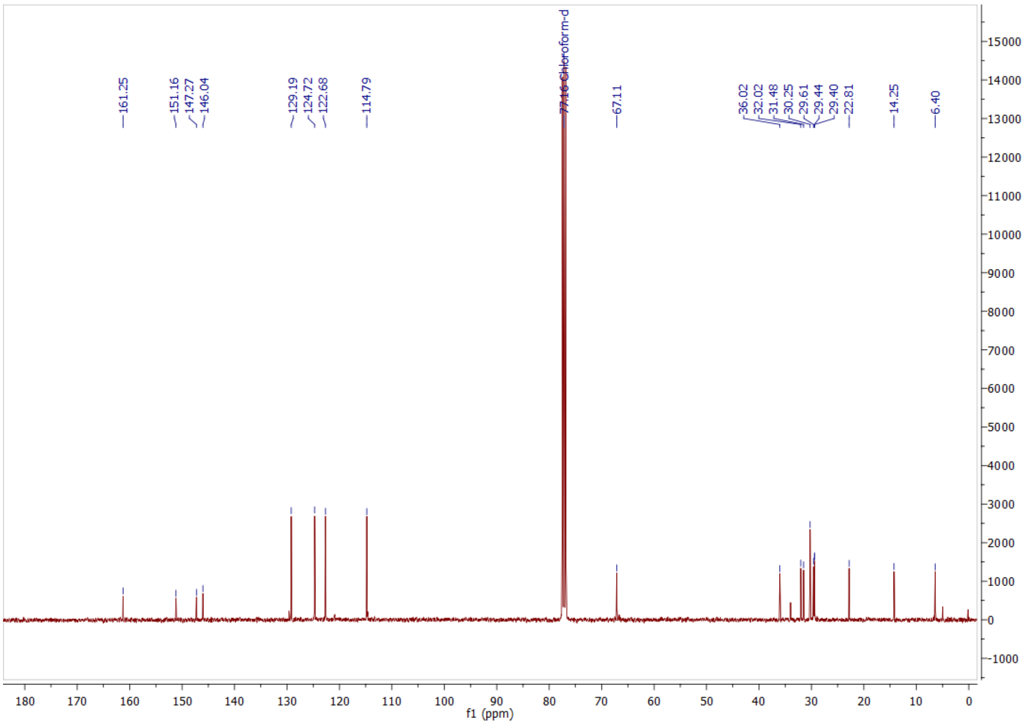

Figure S37. ^{13}C NMR spectrum of compound **9d** (101 MHz, $CDCl_3$)

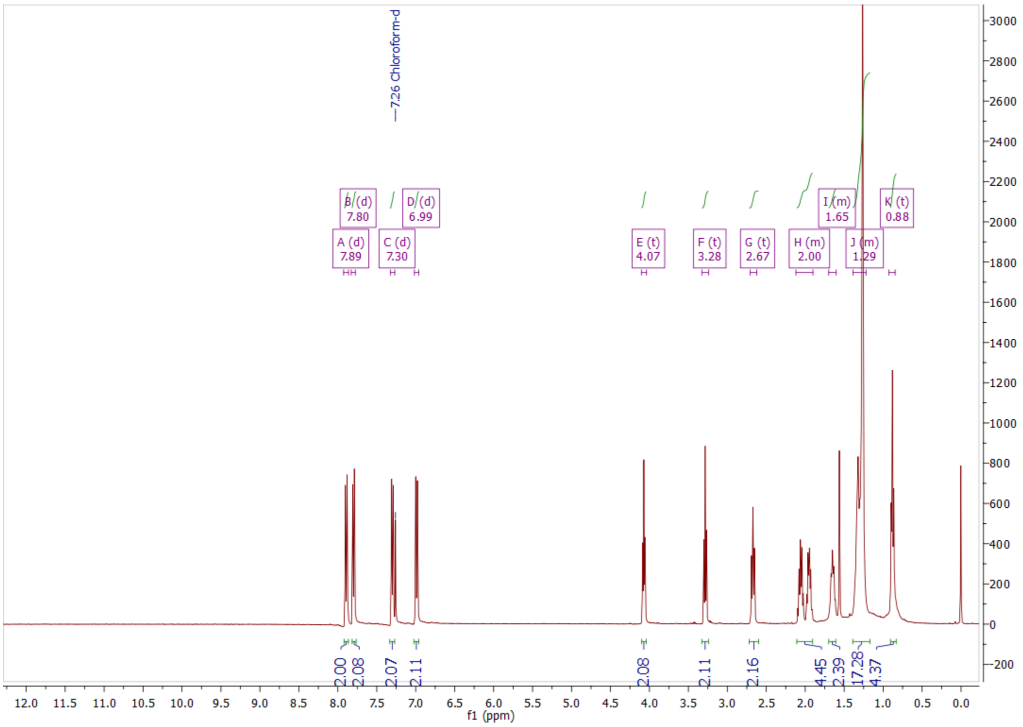

Figure S38. ^{1}H NMR spectrum of compound **9e** (400 MHz, $CDCl_3$)

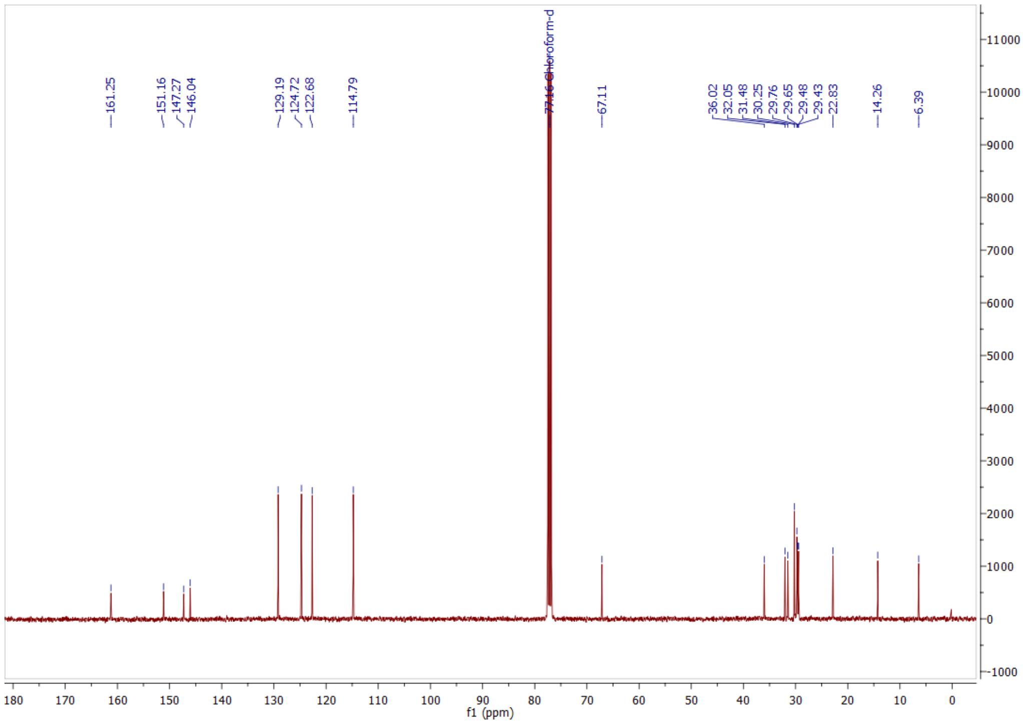

Figure S39. ^{13}C NMR spectrum of compound **9e** (101 MHz, $CDCl_3$)

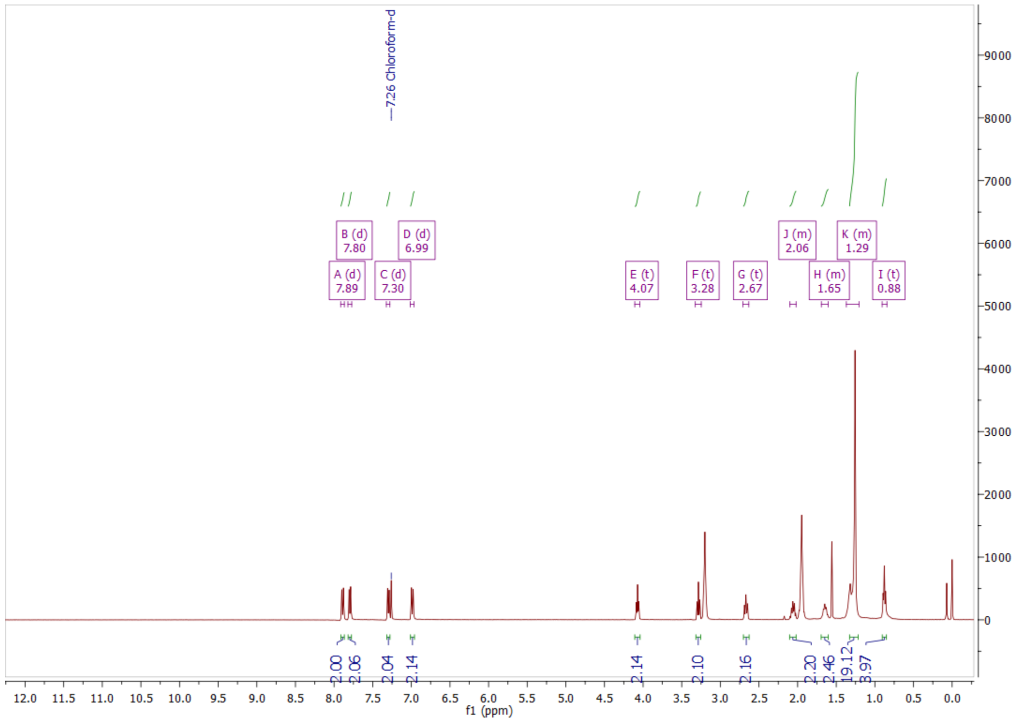

Figure S40. ^{1}H NMR spectrum of compound **9f** (400 MHz, $CDCl_3$)

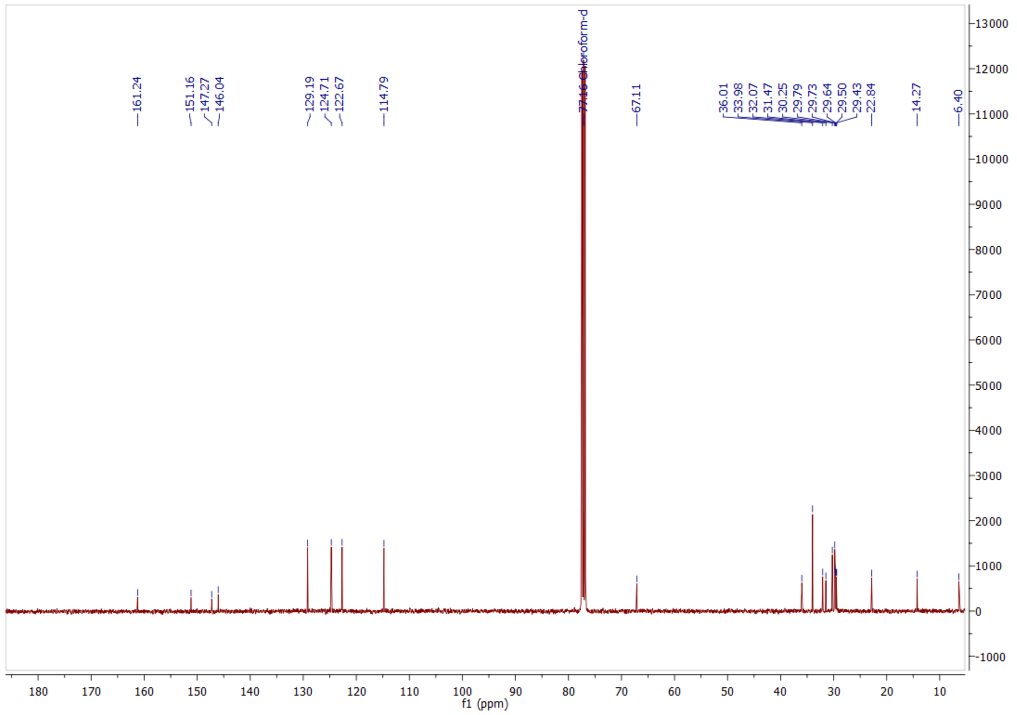

Figure S41. ^{13}C NMR spectrum of compound **9f** (101 MHz, $CDCl_3$)

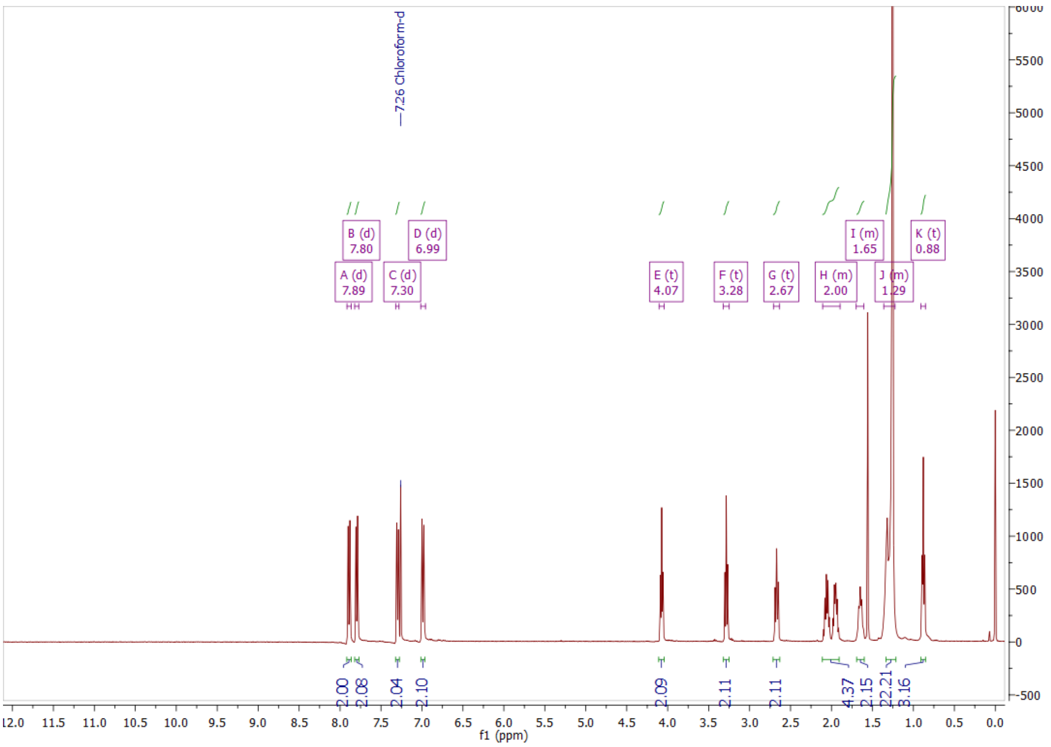

Figure S42. ^{1}H NMR spectrum of compound **9g** (400 MHz, $CDCl_3$)

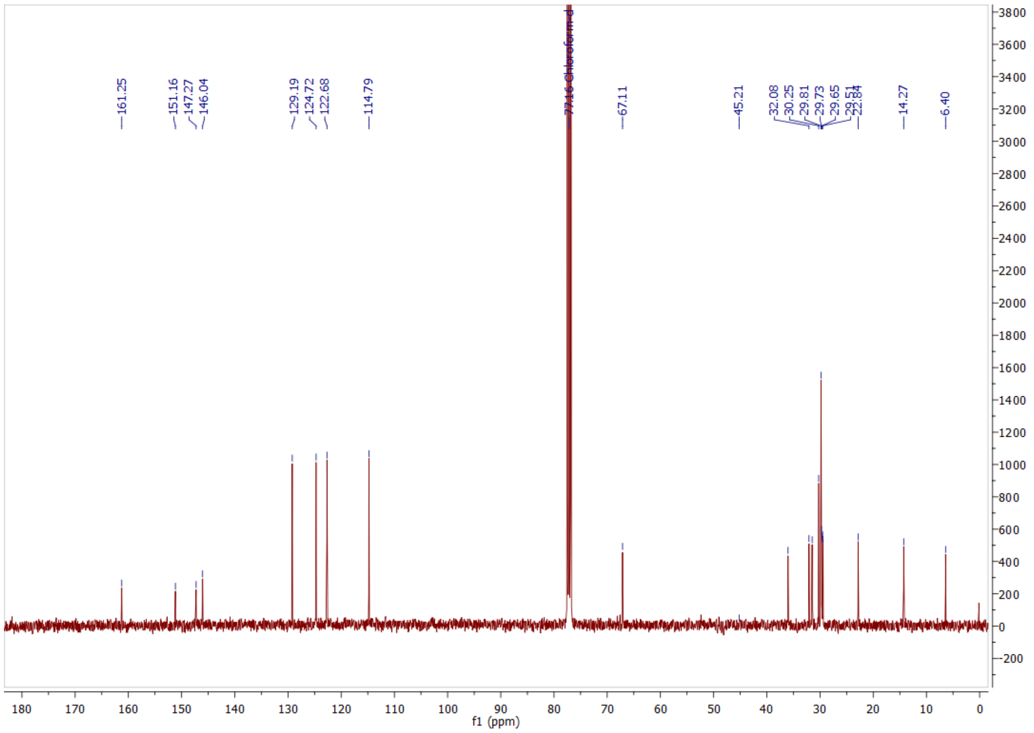

Figure S43. ^{13}C NMR spectrum of compound **9g** (101 MHz, $CDCl_3$)

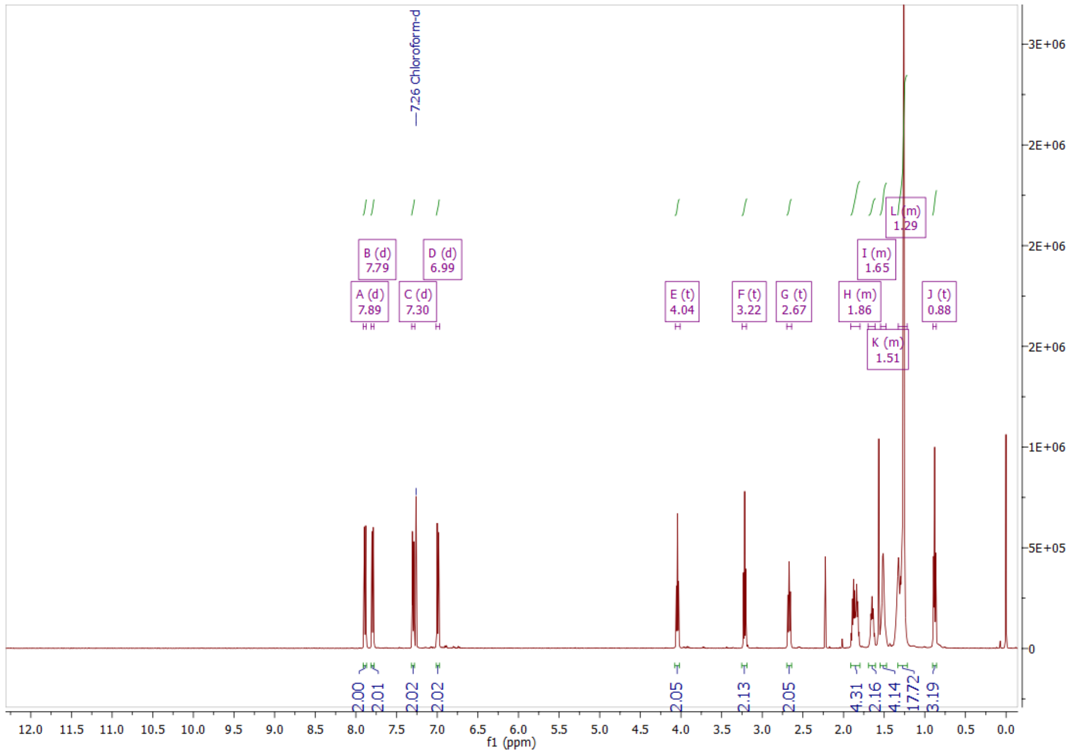

Figure S44. ^{1}H NMR spectrum of compound **11a** (400 MHz, $CDCl_3$)

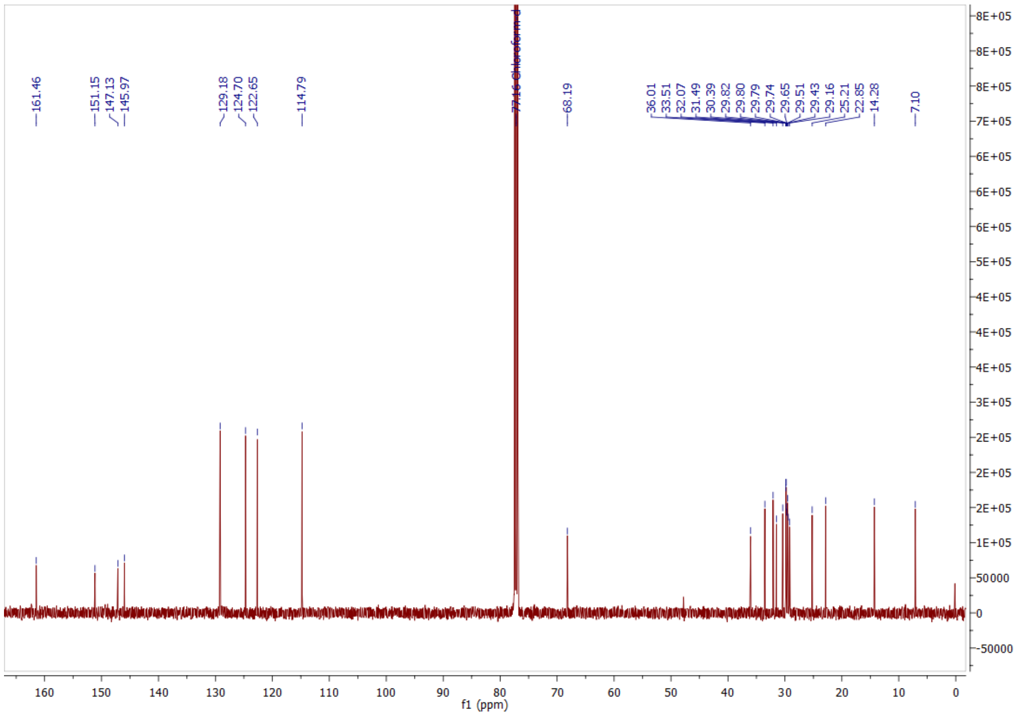

Figure S45. ^{13}C NMR spectrum of compound **11a** (101 MHz, $CDCl_3$)

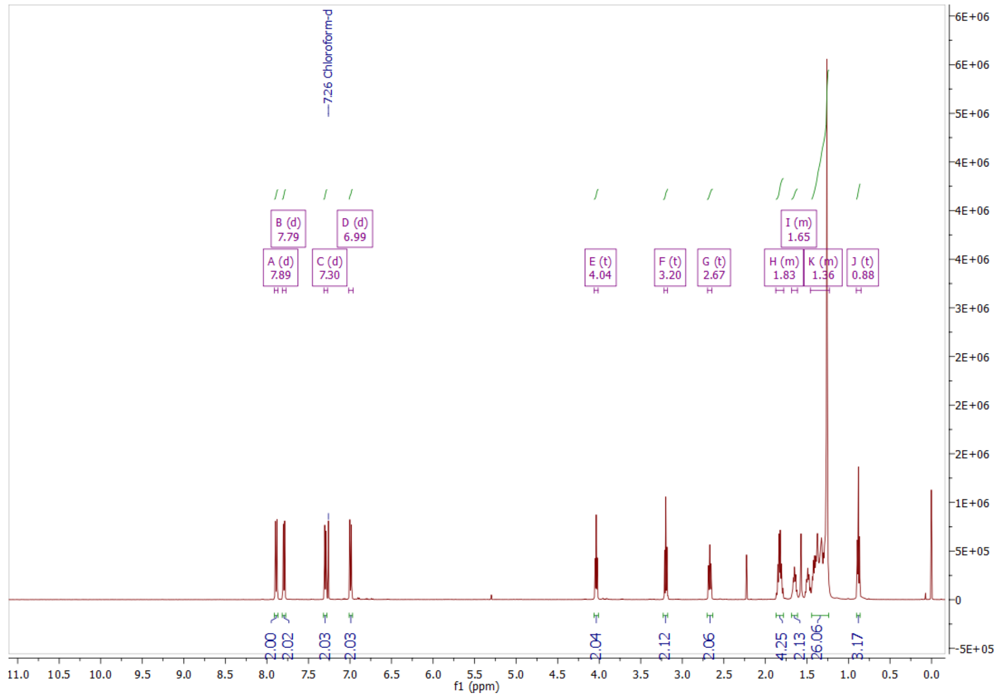

Figure S46. ^{1}H NMR spectrum of compound **11b** (400 MHz, $CDCl_3$)

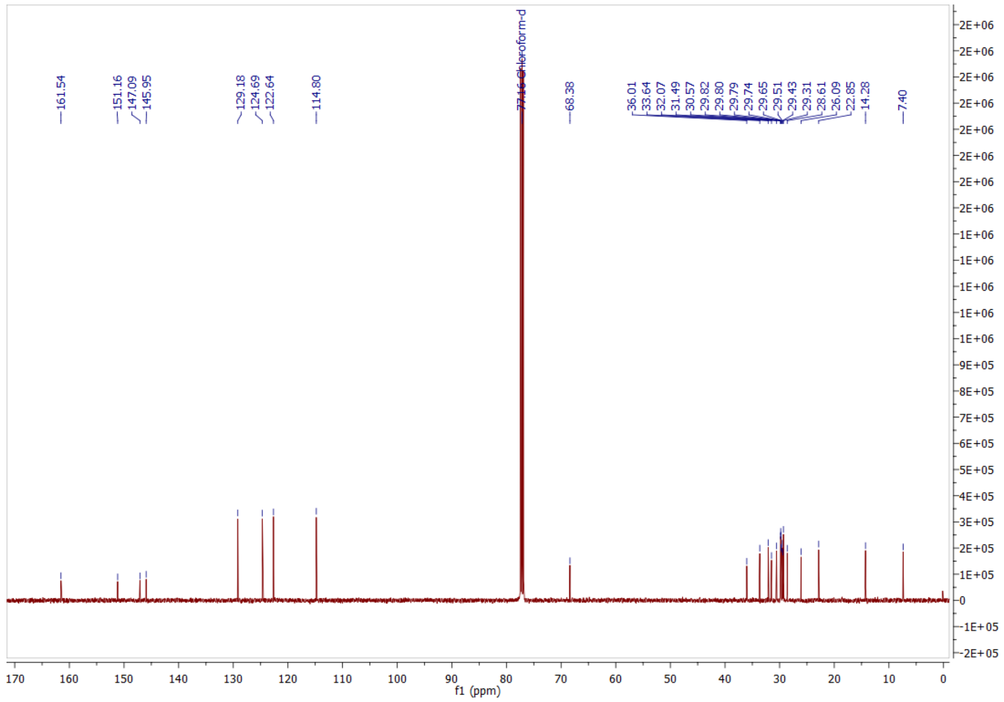

Figure S47. ^{13}C NMR spectrum of compound **11b** (101 MHz, $CDCl_3$)

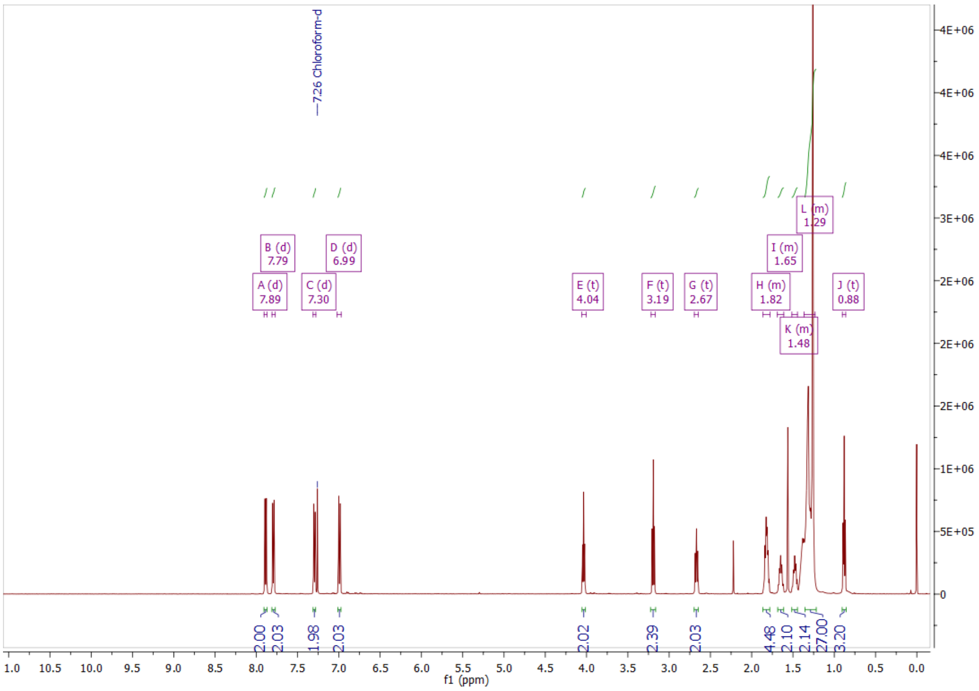

Figure S48. ^{1}H NMR spectrum of compound **11c** (400 MHz, $CDCl_3$)

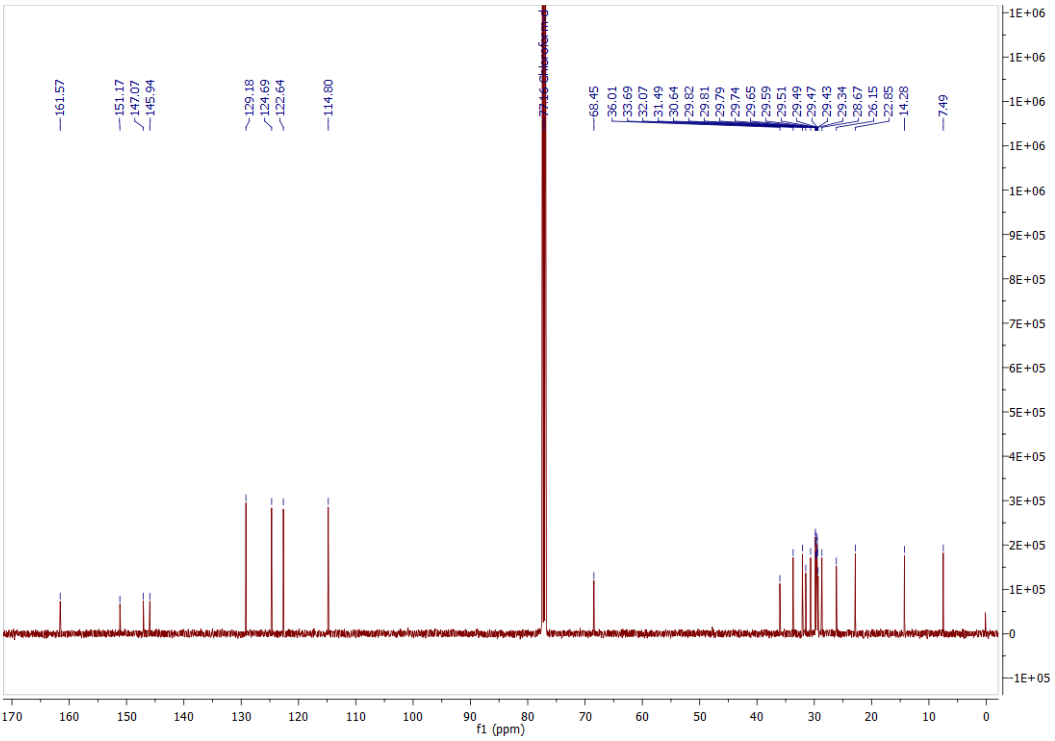

Figure S49. ^{13}C NMR spectrum of compound **11c** (101 MHz, $CDCl_3$)

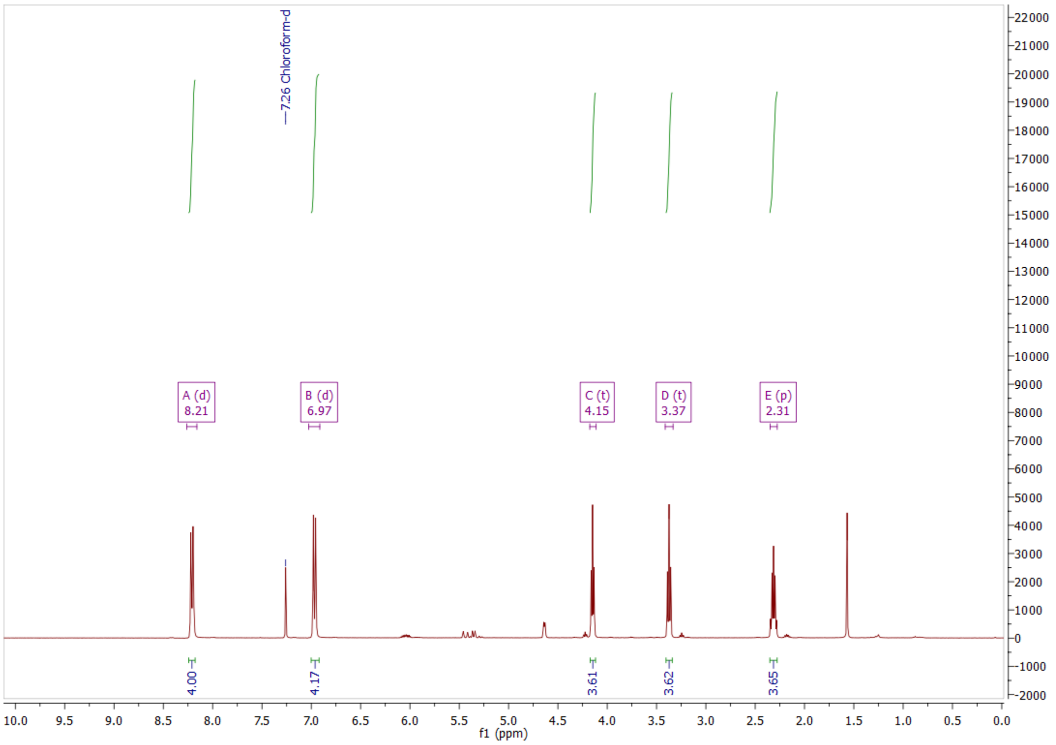

Figure S50. ^{1}H NMR spectrum of compound **14a** (400 MHz, $CDCl_3$)

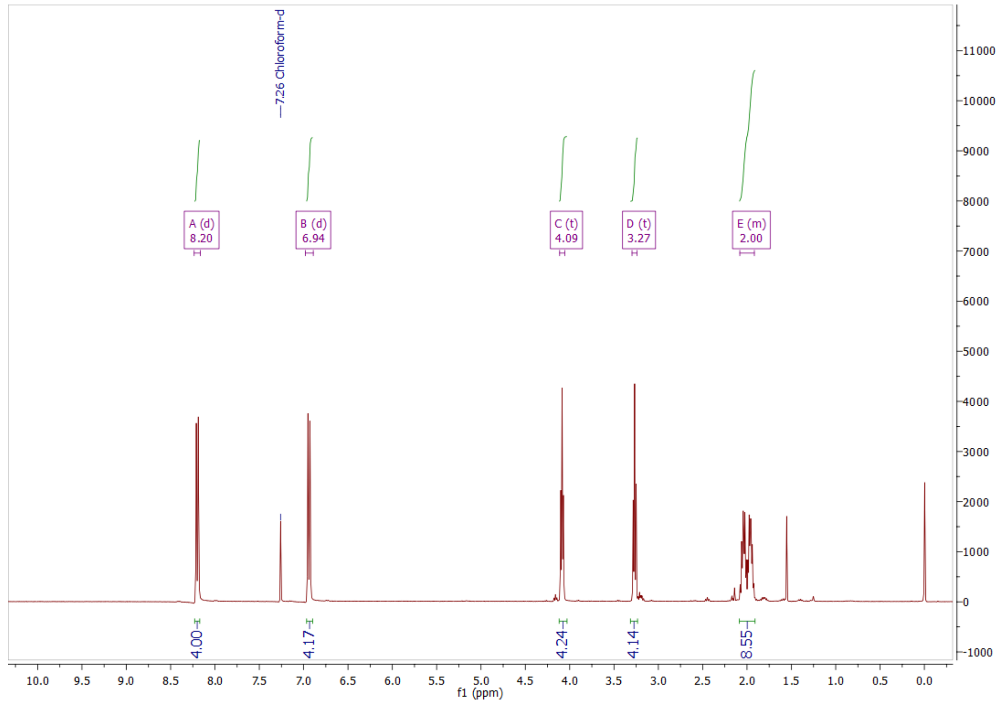

Figure S51. ^{1}H NMR spectrum of compound **14b** (400 MHz, $CDCl_3$)

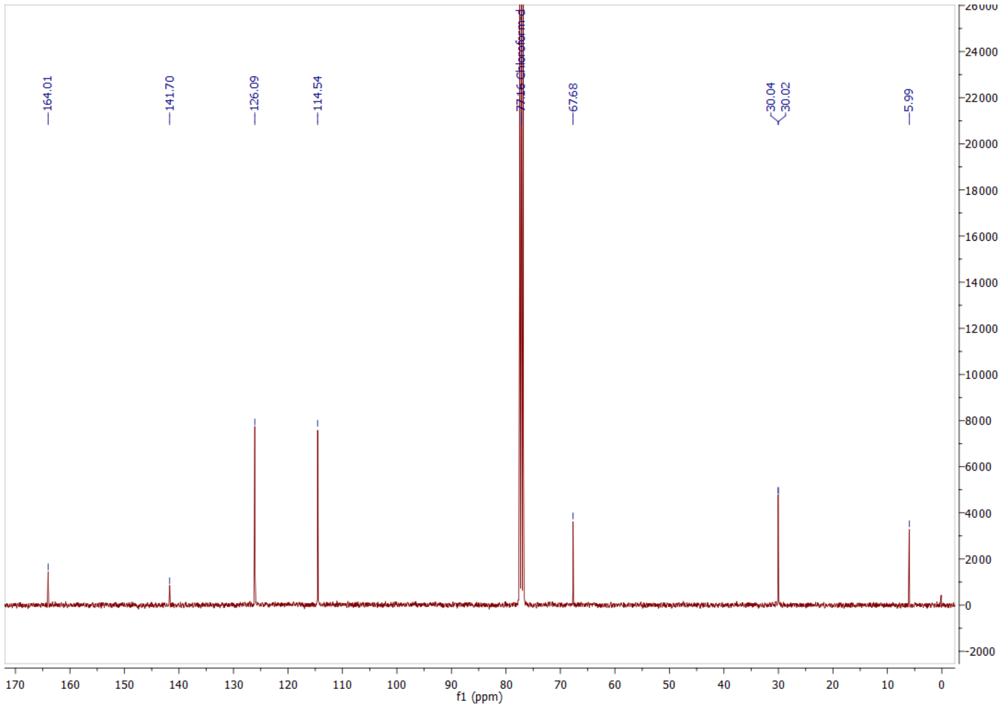

Figure S52. ^{13}C NMR spectrum of compound **14b** (101 MHz, $CDCl_3$)

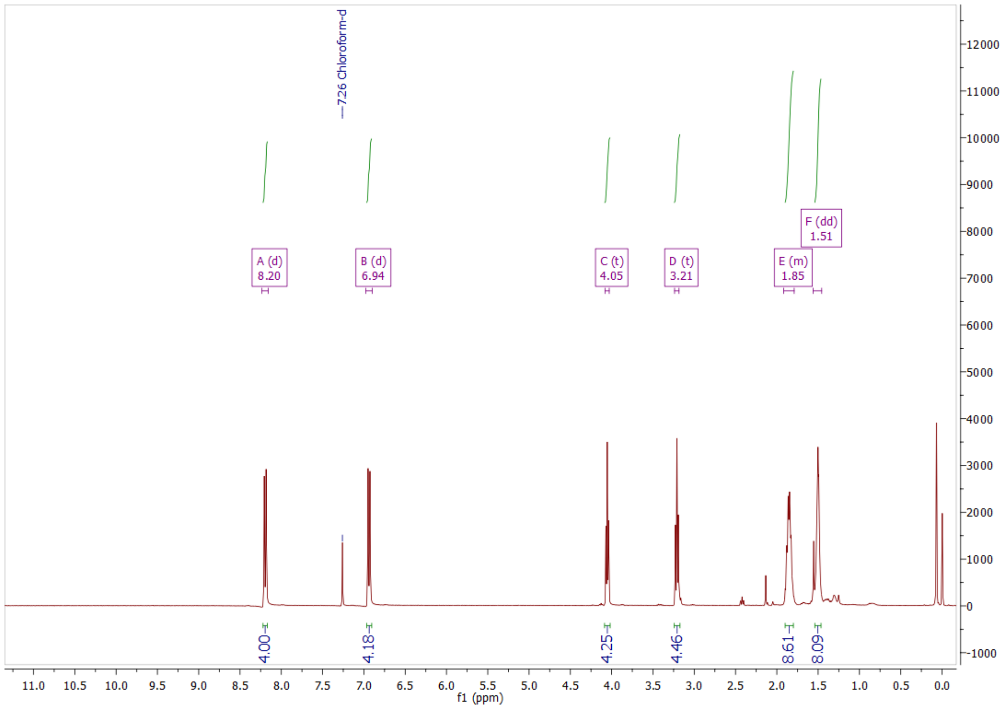

Figure S53. ^{1}H NMR spectrum of compound **14c** (400 MHz, $CDCl_3$)

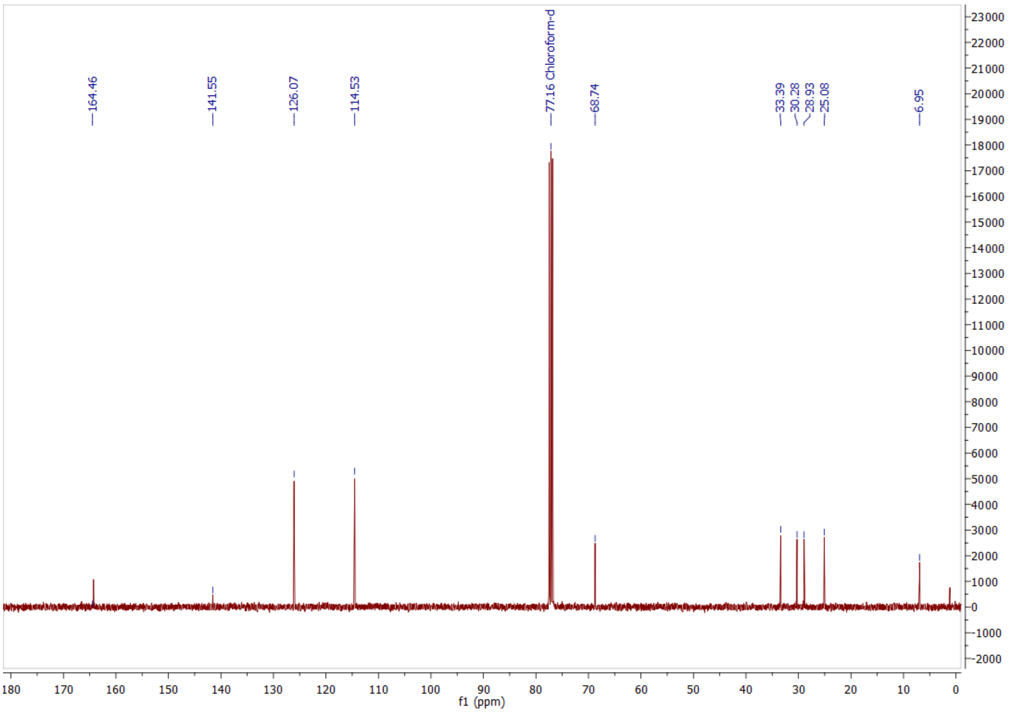

Figure S54. ^{13}C NMR spectrum of compound **14c** (101 MHz, $CDCl_3$)

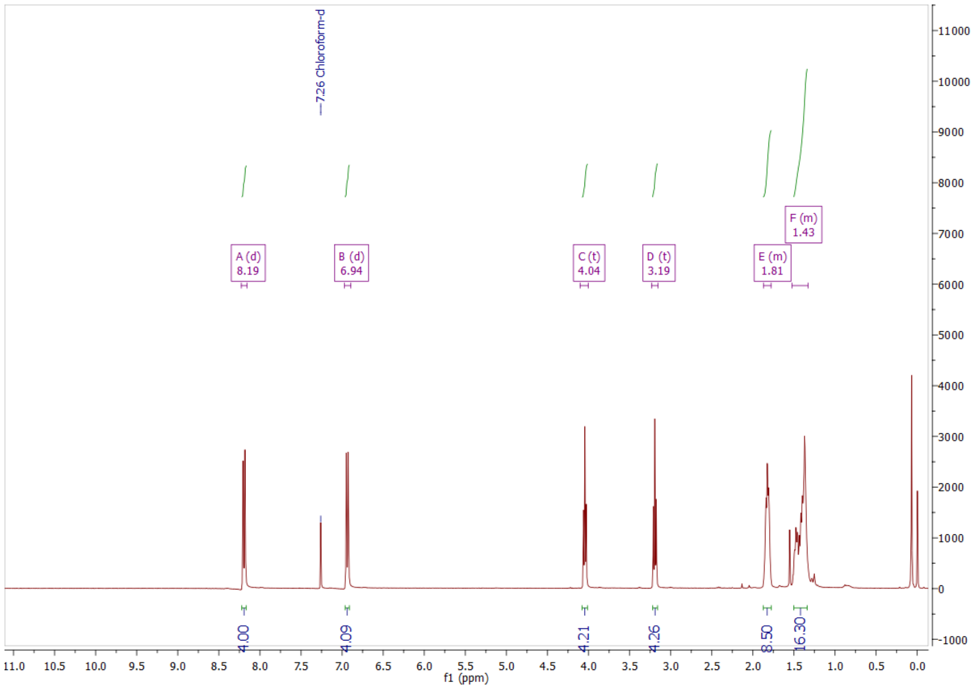

Figure S55. ^{1}H NMR spectrum of compound **14d** (400 MHz, $CDCl_3$)

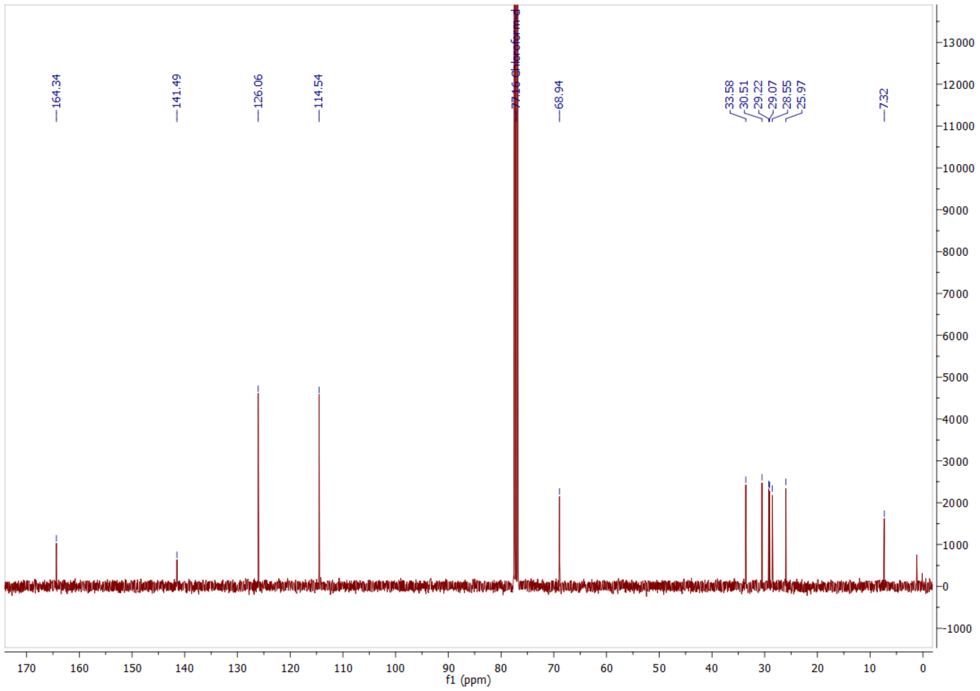

Figure S56. ^{13}C NMR spectrum of compound **14d** (101 MHz, $CDCl_3$)

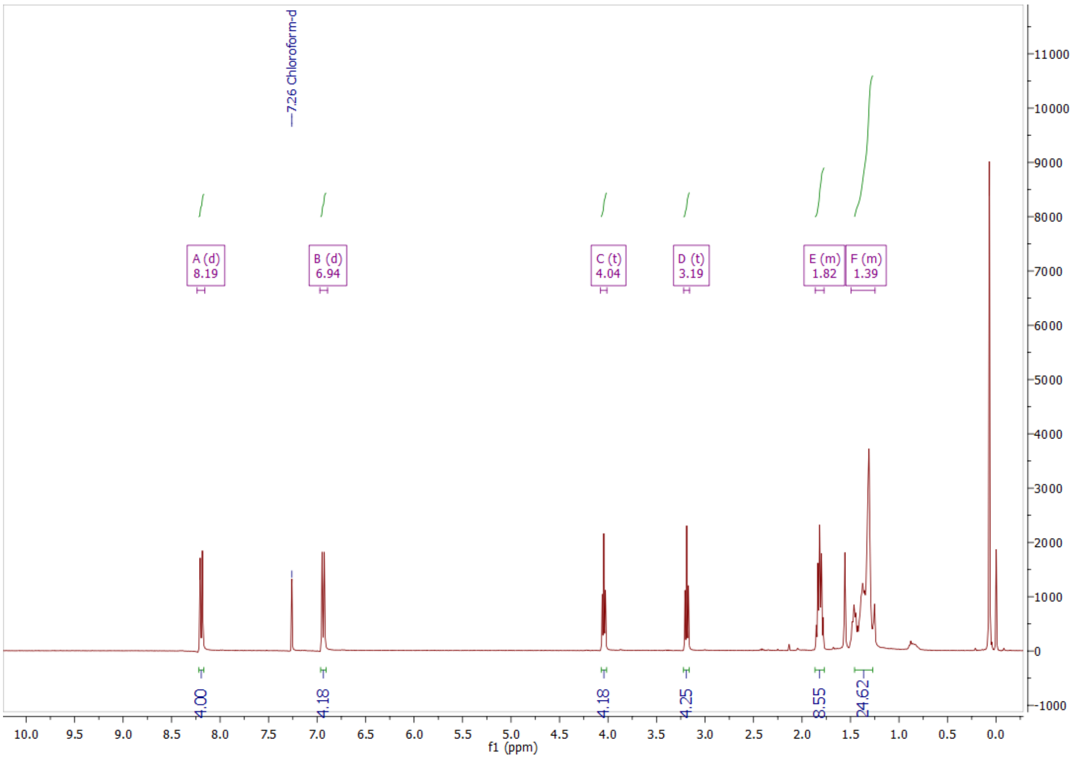

Figure S57. ^{1}H NMR spectrum of compound **14e** (400 MHz, $CDCl_3$)

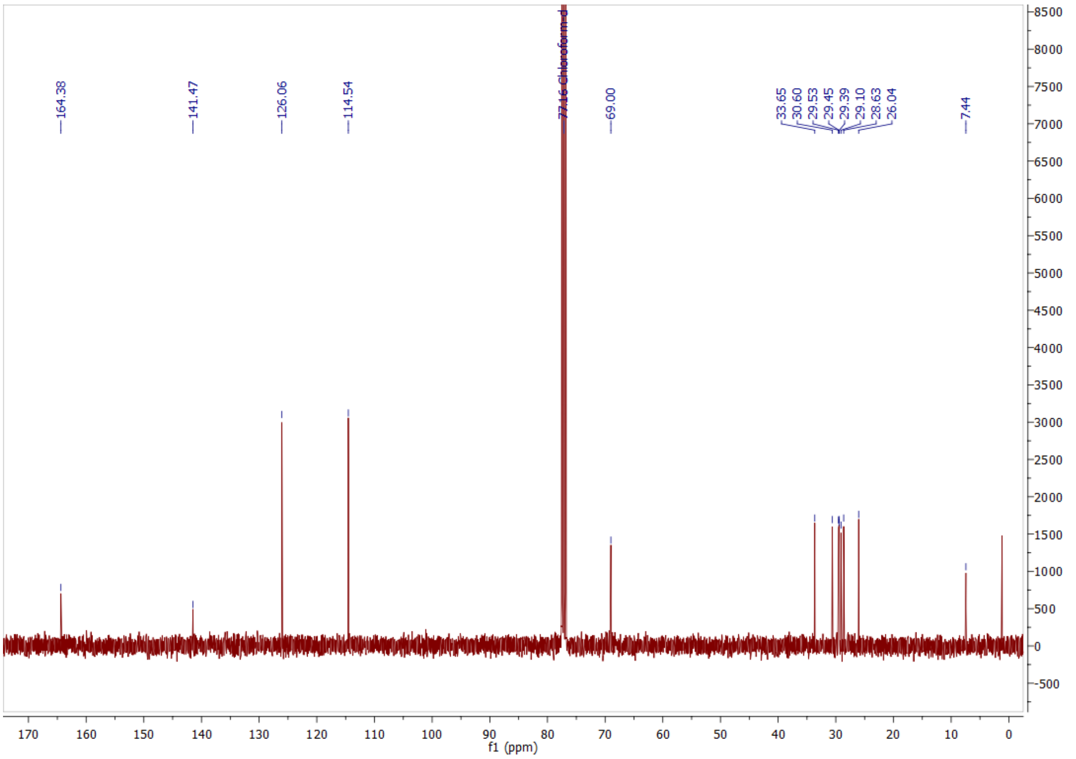

Figure S58. ^{13}C NMR spectrum of compound **14e** (101 MHz, $CDCl_3$)

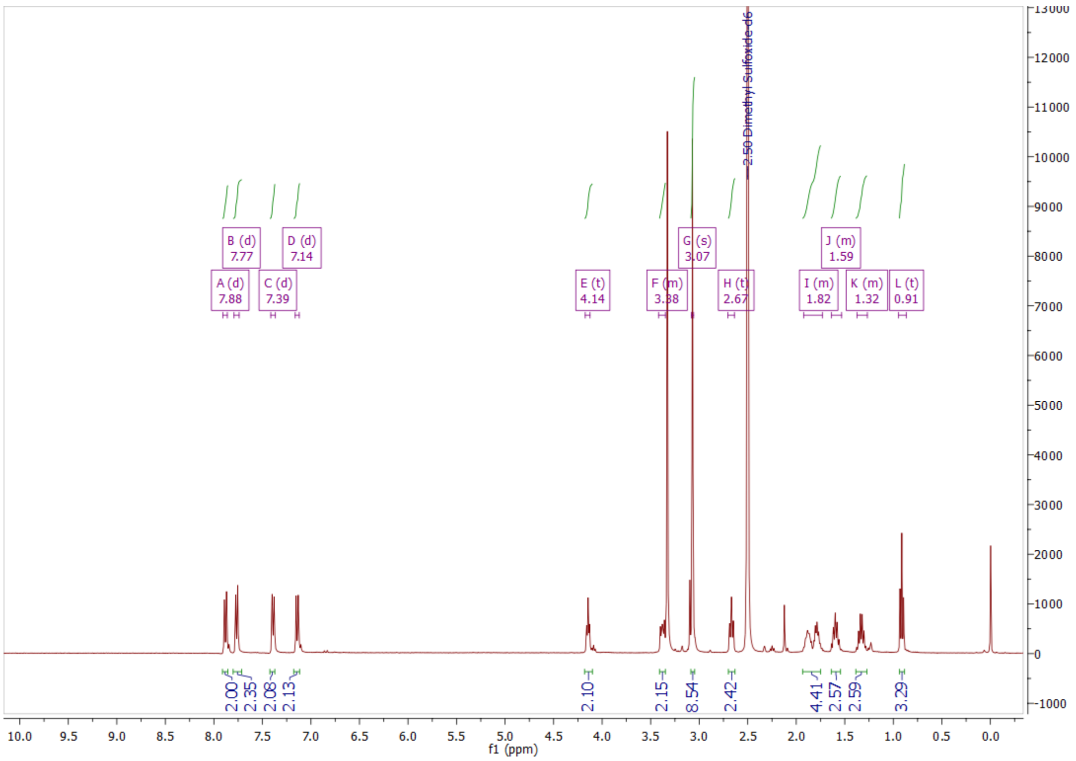

Figure S59. ^{1}H NMR spectrum of compound **10a** (400 MHz, DMSO-*d6*)

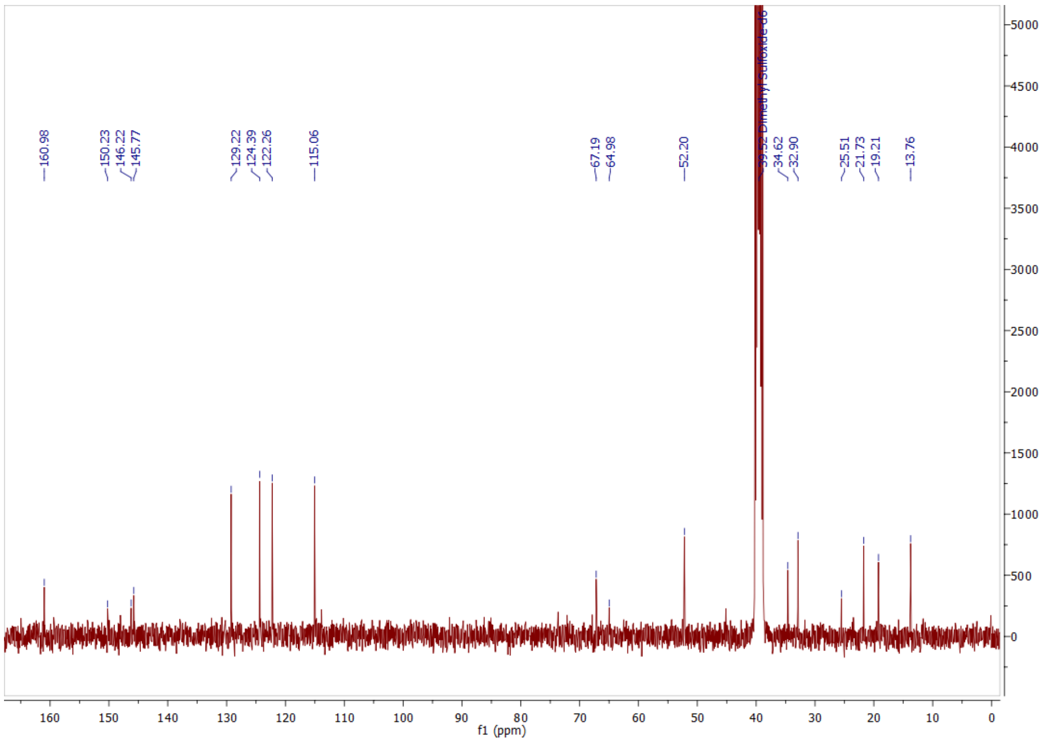

Figure S60. ^{13}C NMR spectrum of compound **10a** (101 MHz, DMSO-*d6*)

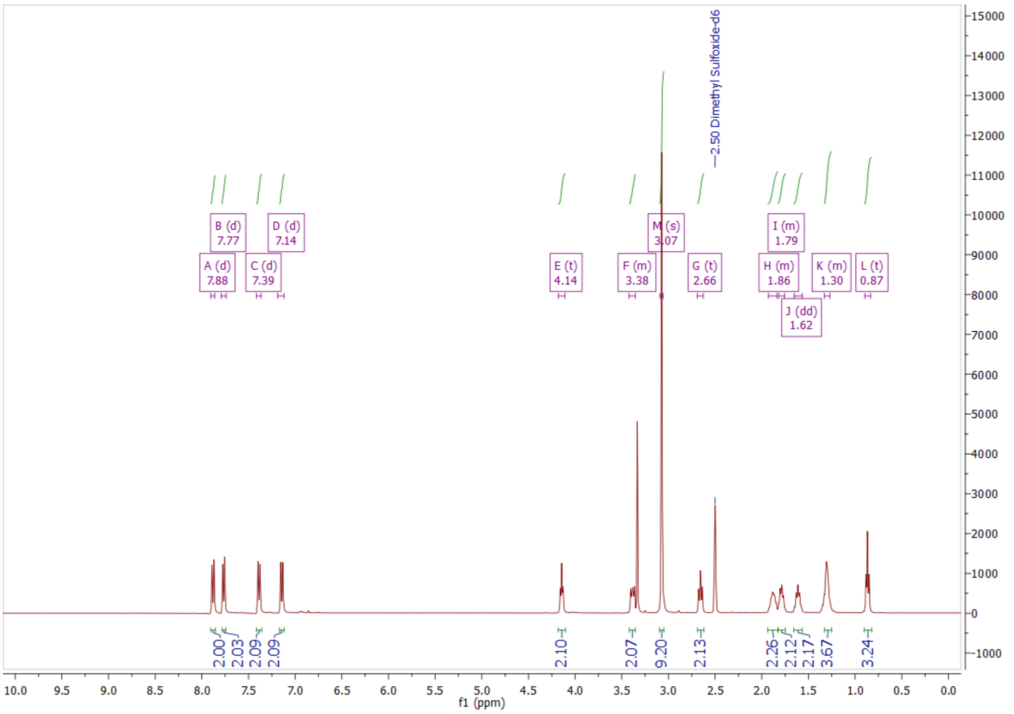

Figure S61. ^{1}H NMR spectrum of compound **10b** (400 MHz, DMSO-*d6*)

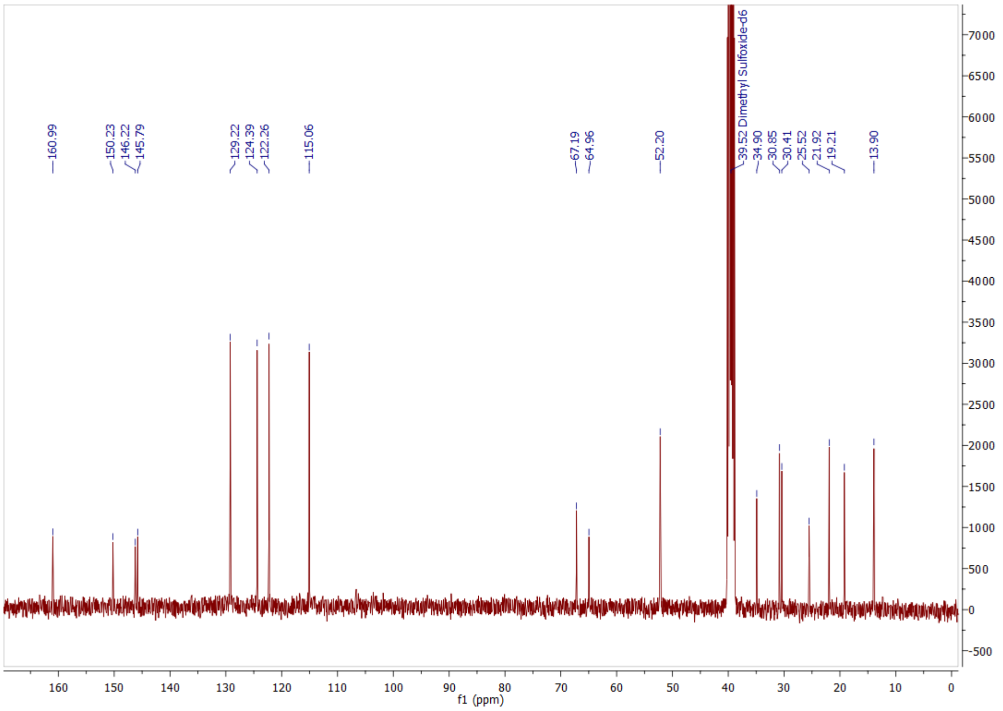

Figure S62. ^{13}C NMR spectrum of compound **10b** (101 MHz, DMSO-*d6*)

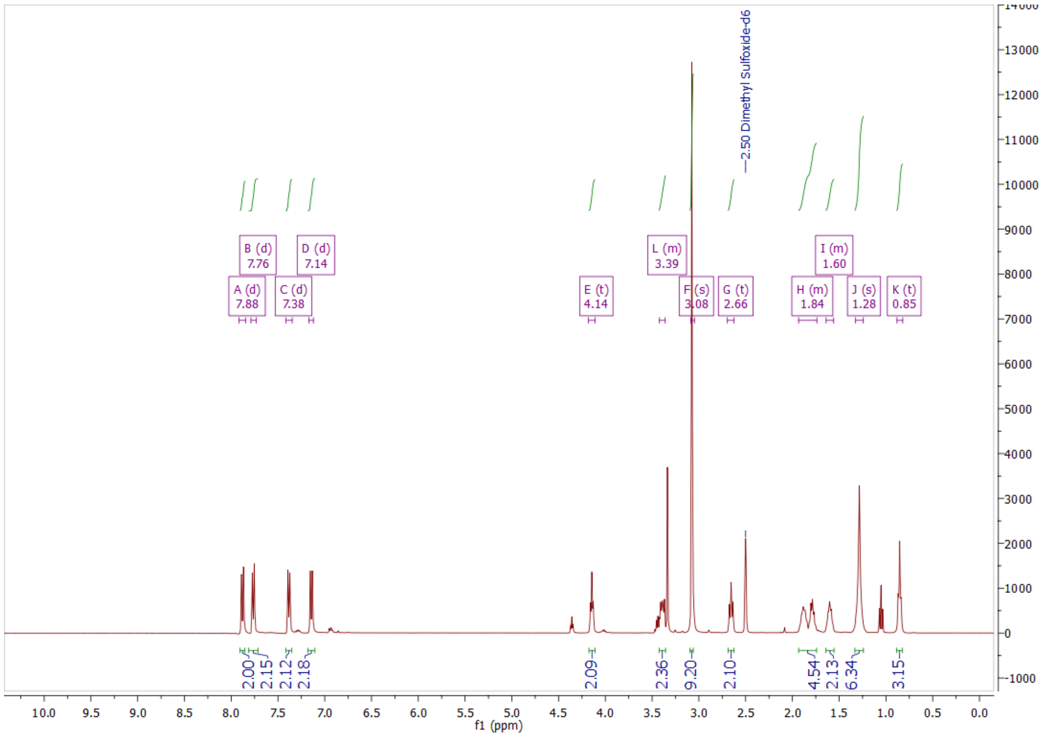

Figure S63. ^{1}H NMR spectrum of compound **10c** (400 MHz, DMSO-*d6*)

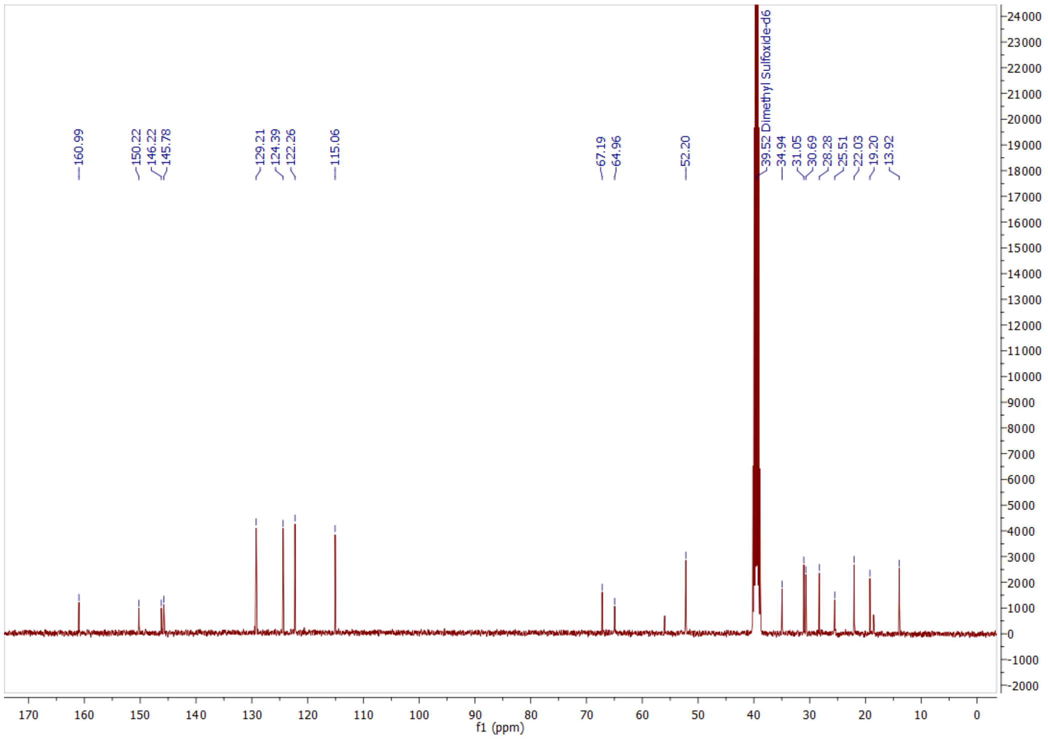

Figure S64. ^{13}C NMR spectrum of compound **10c** (101 MHz, DMSO-*d6*)

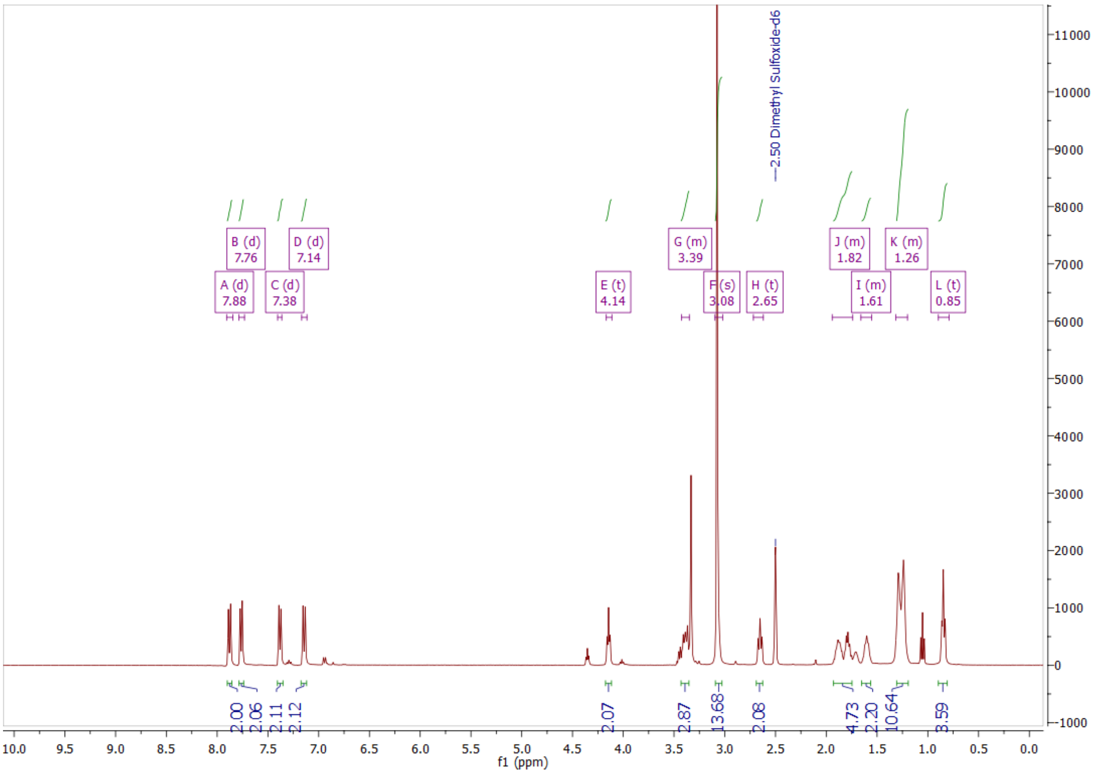

Figure S65. ^{1}H NMR spectrum of compound **10d** (400 MHz, DMSO-*d6*)

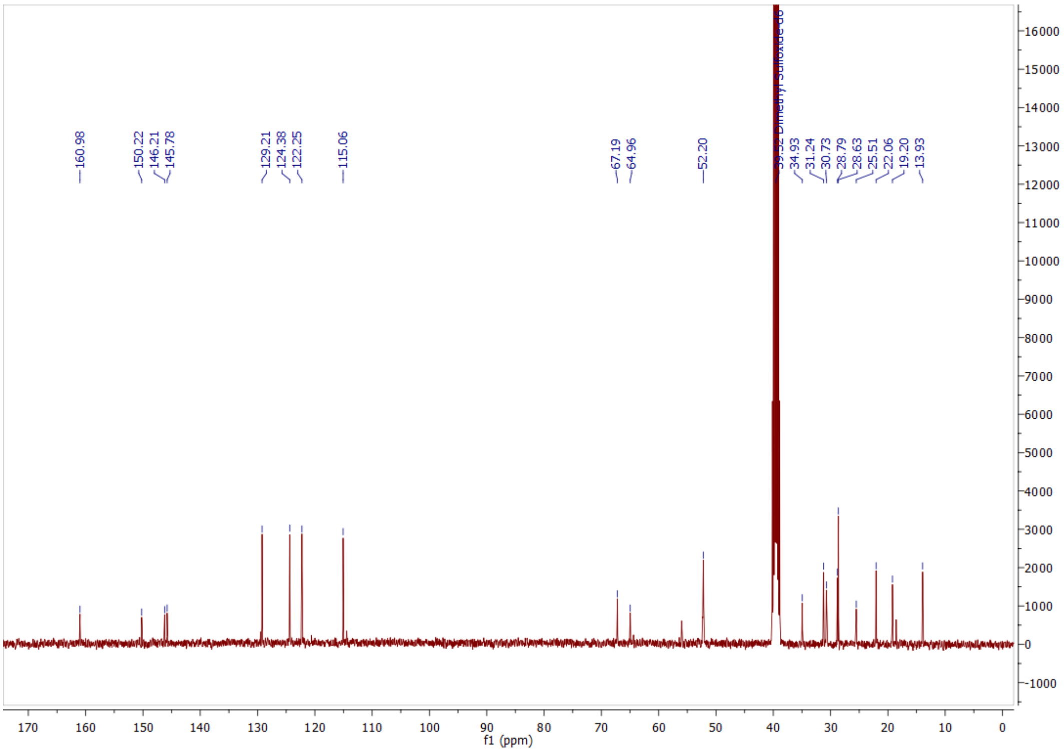

Figure S66. ^{13}C NMR spectrum of compound **10d** (101 MHz, DMSO-*d6*)

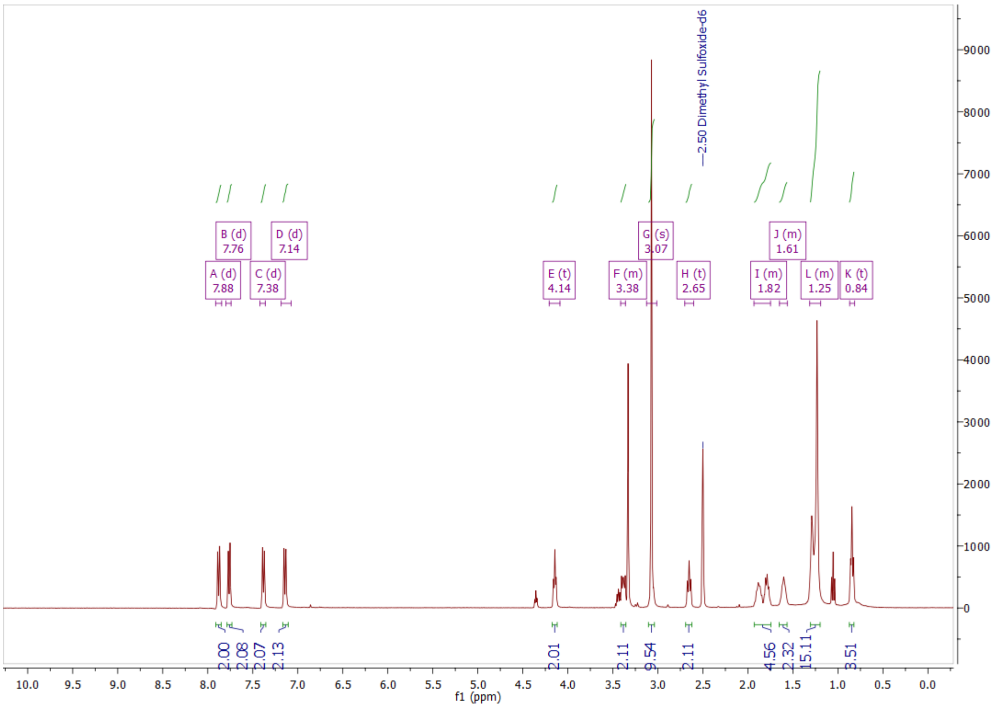

Figure S67. ^{1}H NMR spectrum of compound **10e** (400 MHz, DMSO-*d6*)

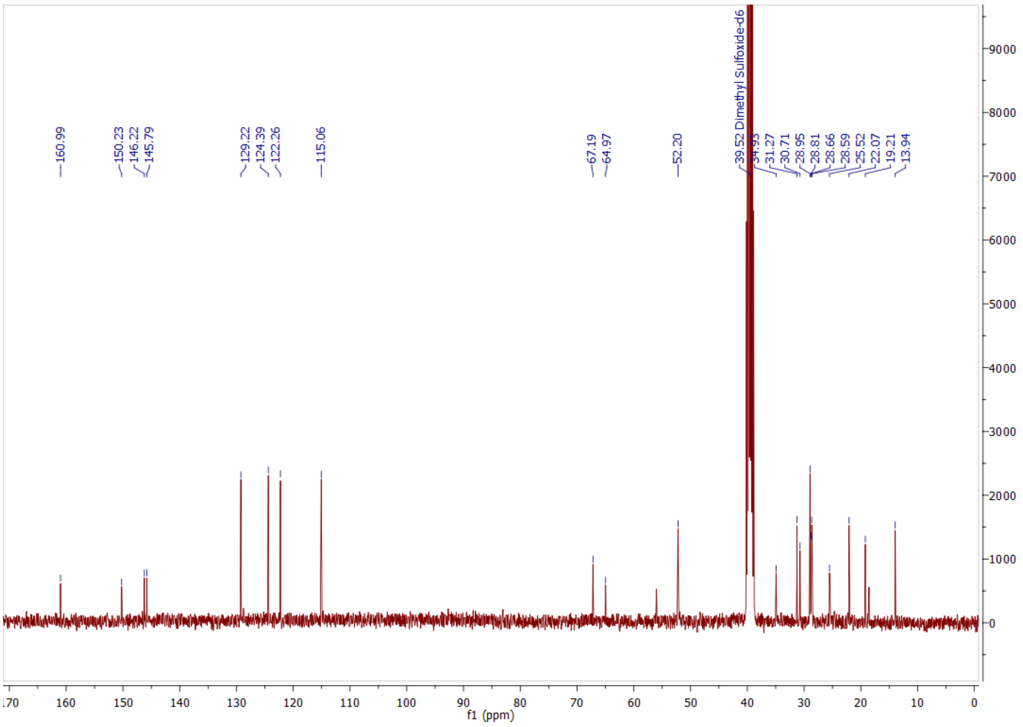

Figure S68. ^{13}C NMR spectrum of compound **10e** (101 MHz, DMSO-*d6*)

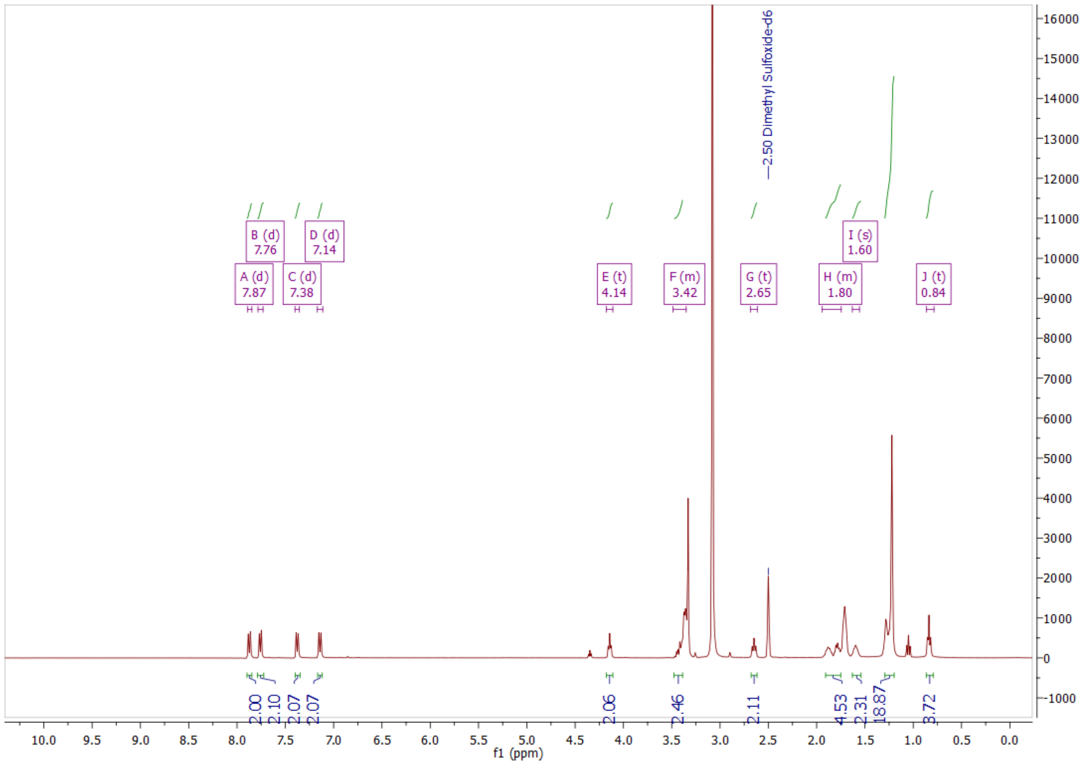

Figure S69. ^{1}H NMR spectrum of compound **10f** (400 MHz, DMSO-*d6*)

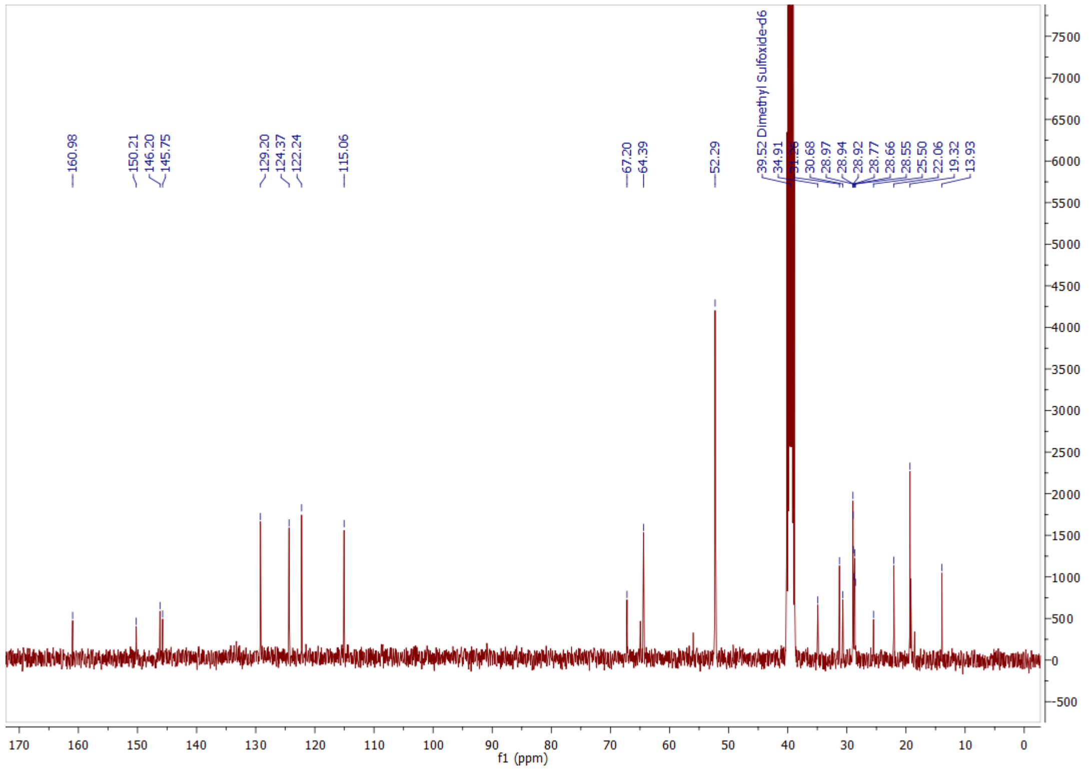

Figure S70. ^{13}C NMR spectrum of compound **10f** (101 MHz, DMSO-*d6*)

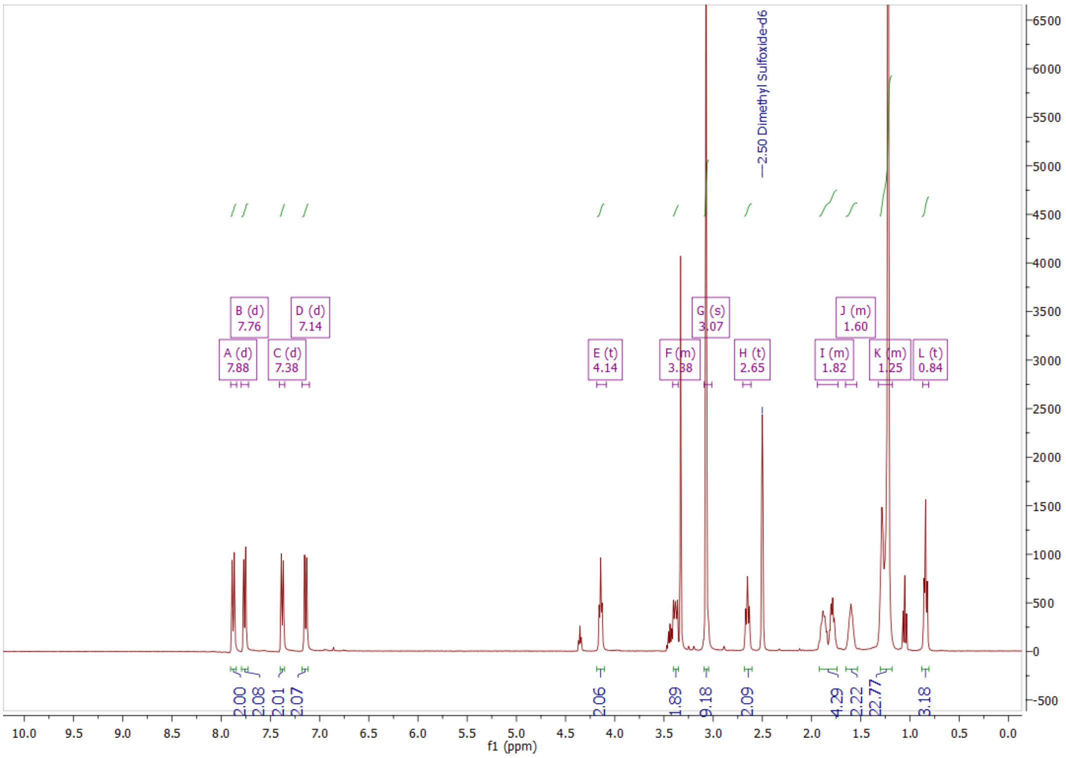

Figure S71. ^{1}H NMR spectrum of compound **10g** (400 MHz, DMSO-*d6*)

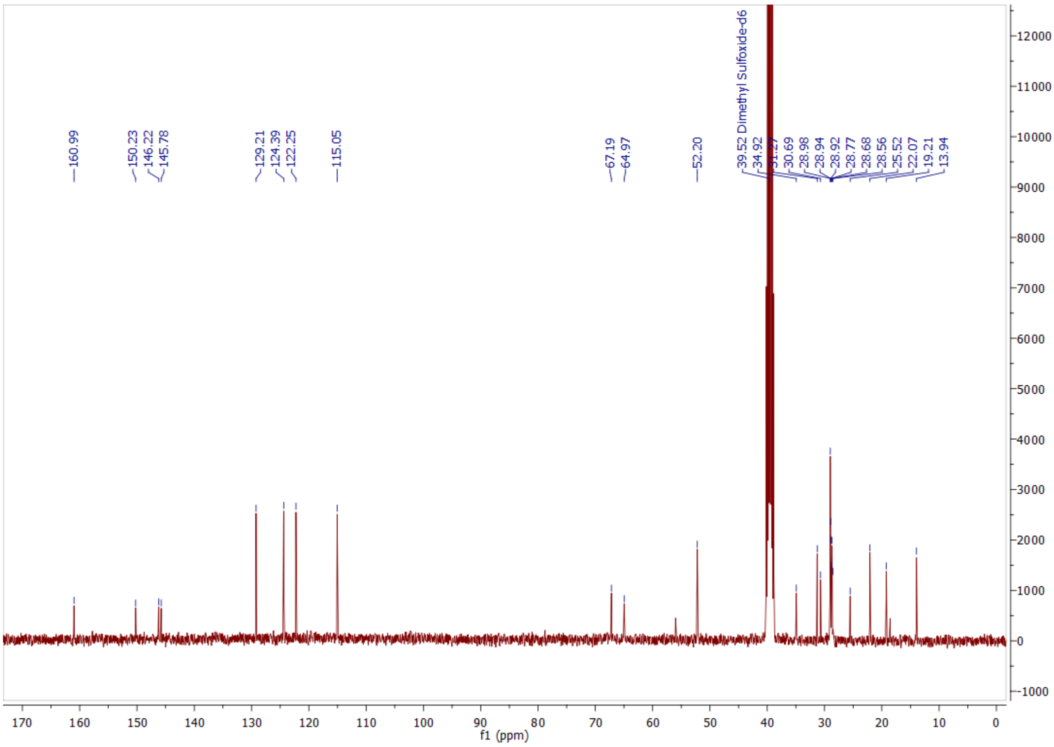

Figure S72. ^{13}C NMR spectrum of compound **10g** (101 MHz, DMSO-*d6*)

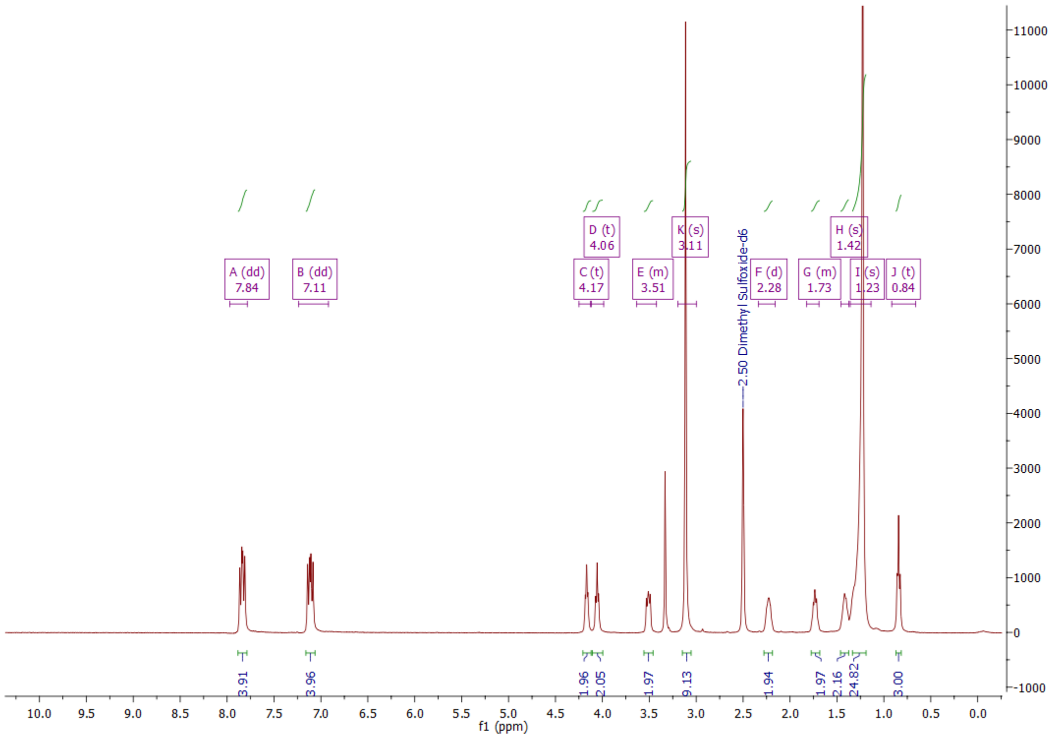

Figure S73. ^{1}H NMR spectrum of compound **10h** (400 MHz, DMSO-*d6*)

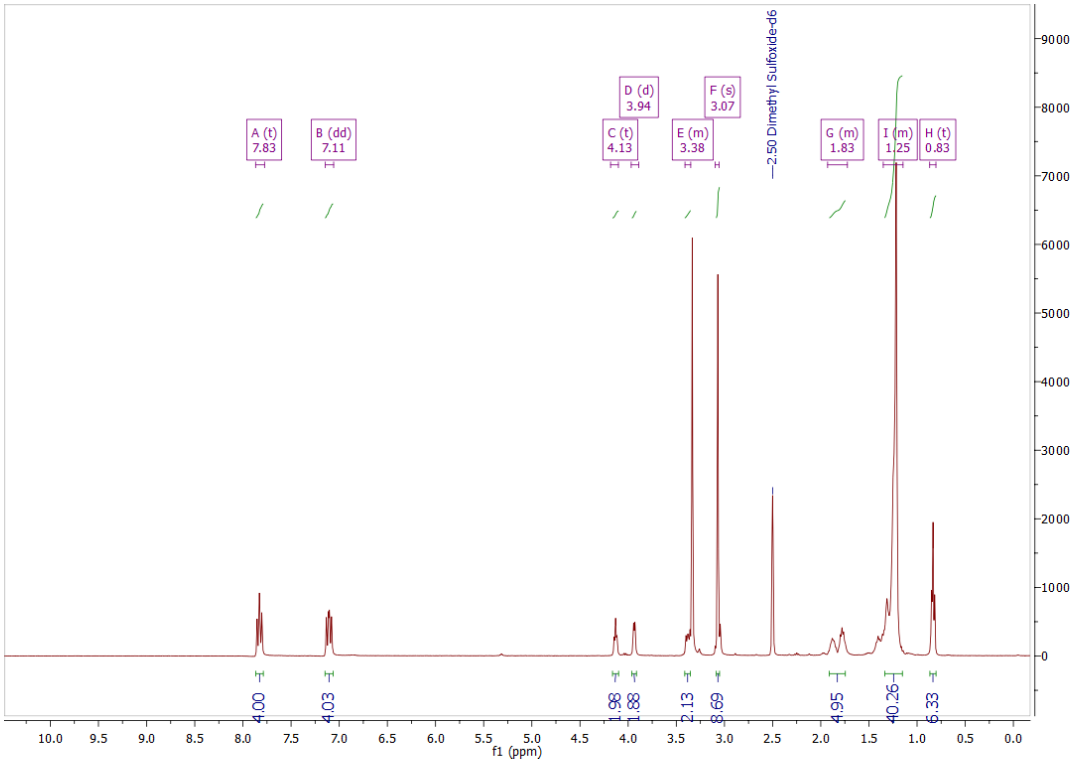

Figure S74. ^{1}H NMR spectrum of compound **10i** (400 MHz, DMSO-*d6*)

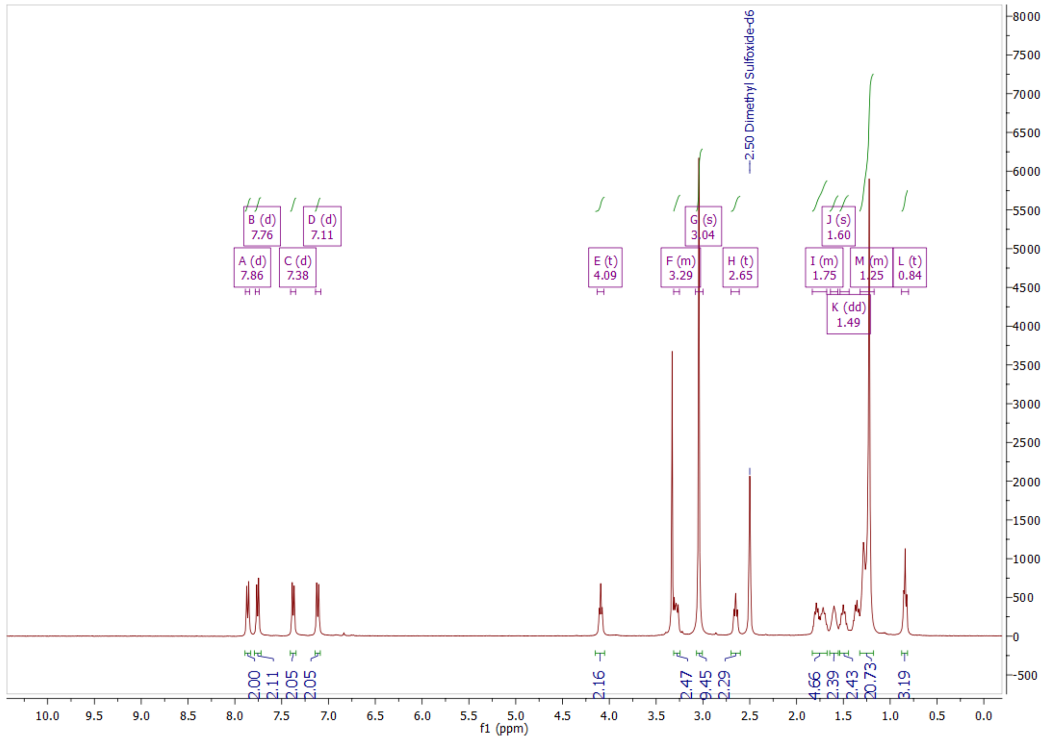

Figure S75. ^{1}H NMR spectrum of compound **12a** (400 MHz, DMSO-*d6*)

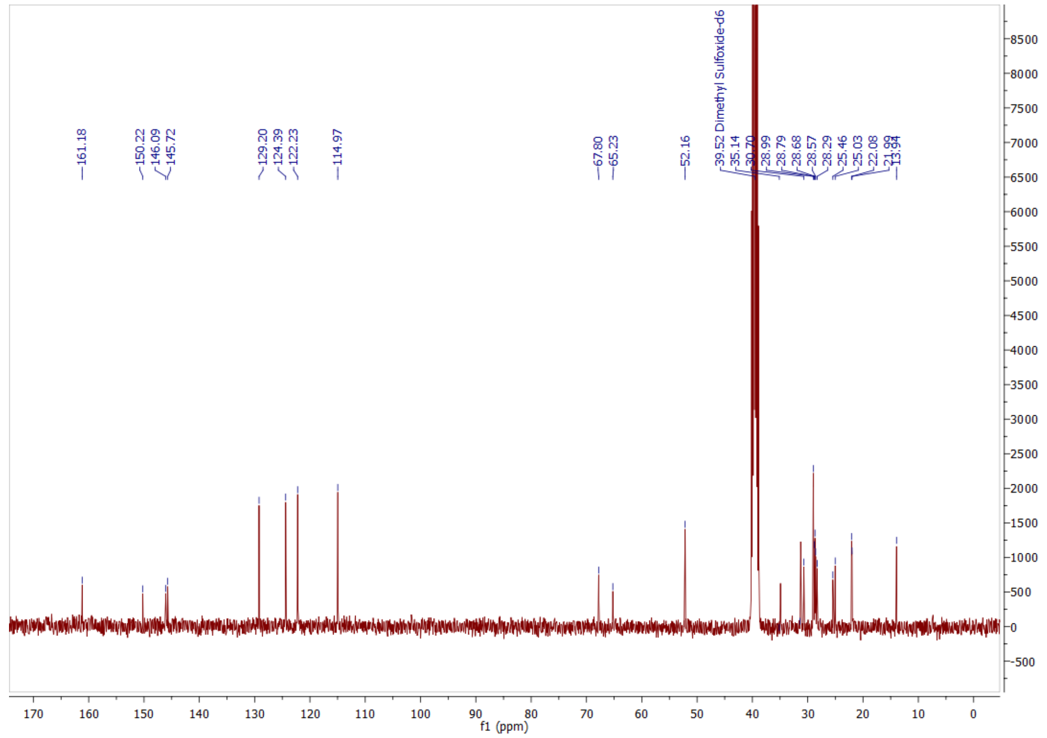

Figure S76. ^{13}C NMR spectrum of compound **12a** (101 MHz, DMSO-*d6*)

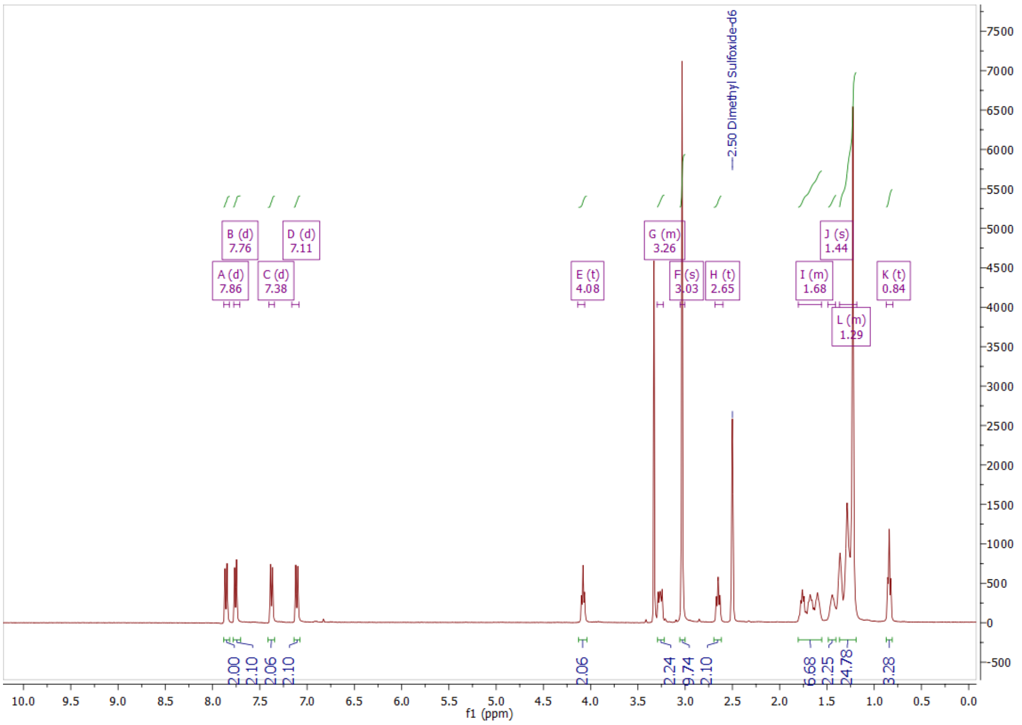

Figure S77. ^{1}H NMR spectrum of compound **12b** (400 MHz, DMSO-*d6*)

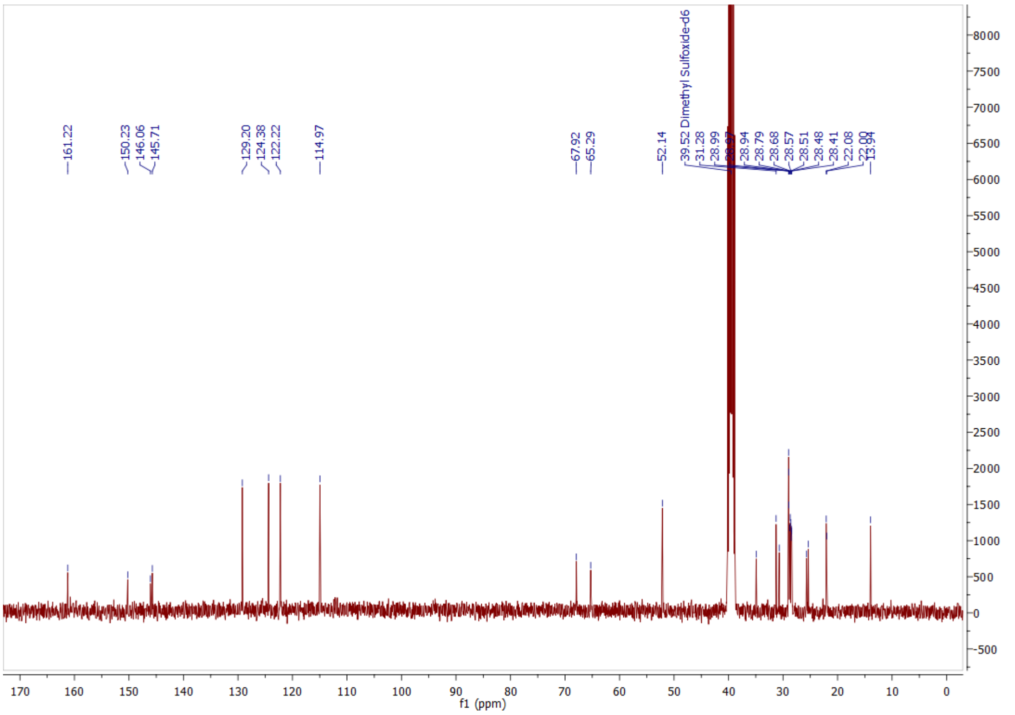

Figure S78. ^{13}C NMR spectrum of compound **12b** (101 MHz, DMSO-*d6*)

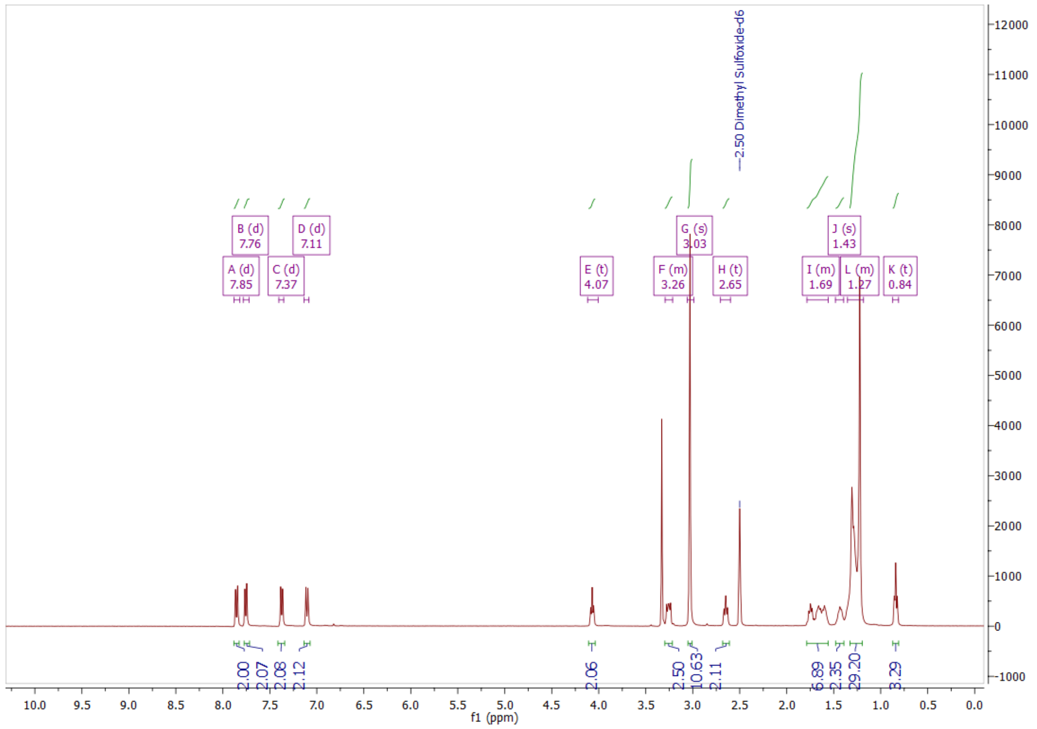

Figure S79. ^{1}H NMR spectrum of compound **12c** (400 MHz, DMSO-*d6*)

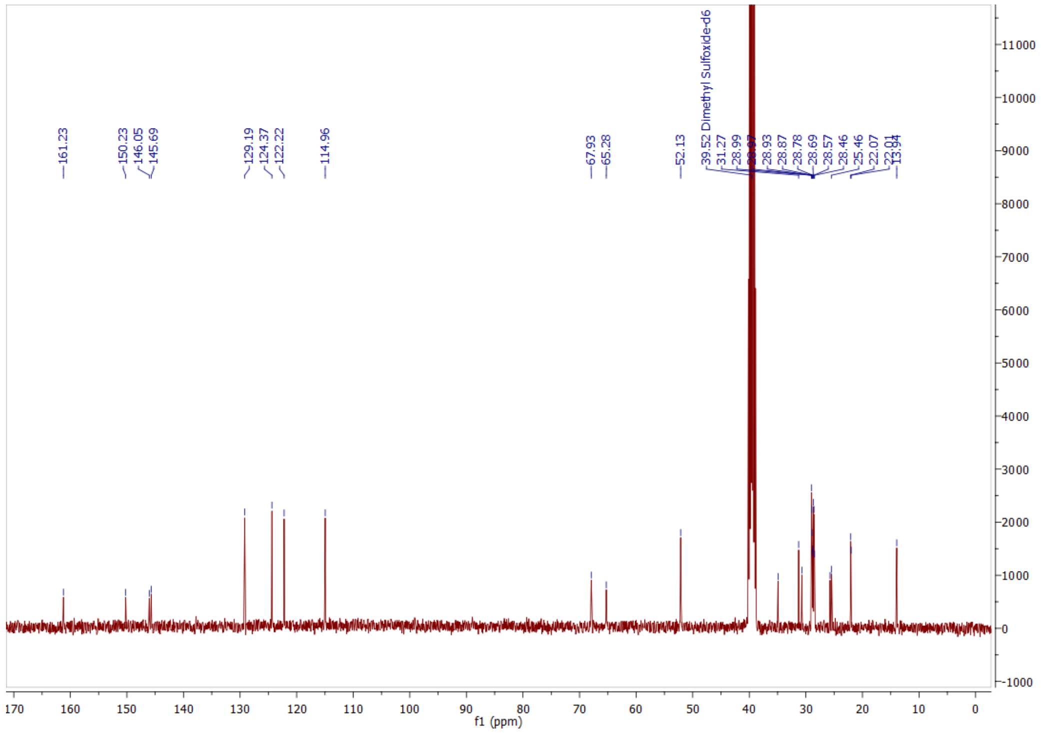

Figure S80. ^{13}C NMR spectrum of compound **12c** (101 MHz, DMSO-*d6*)

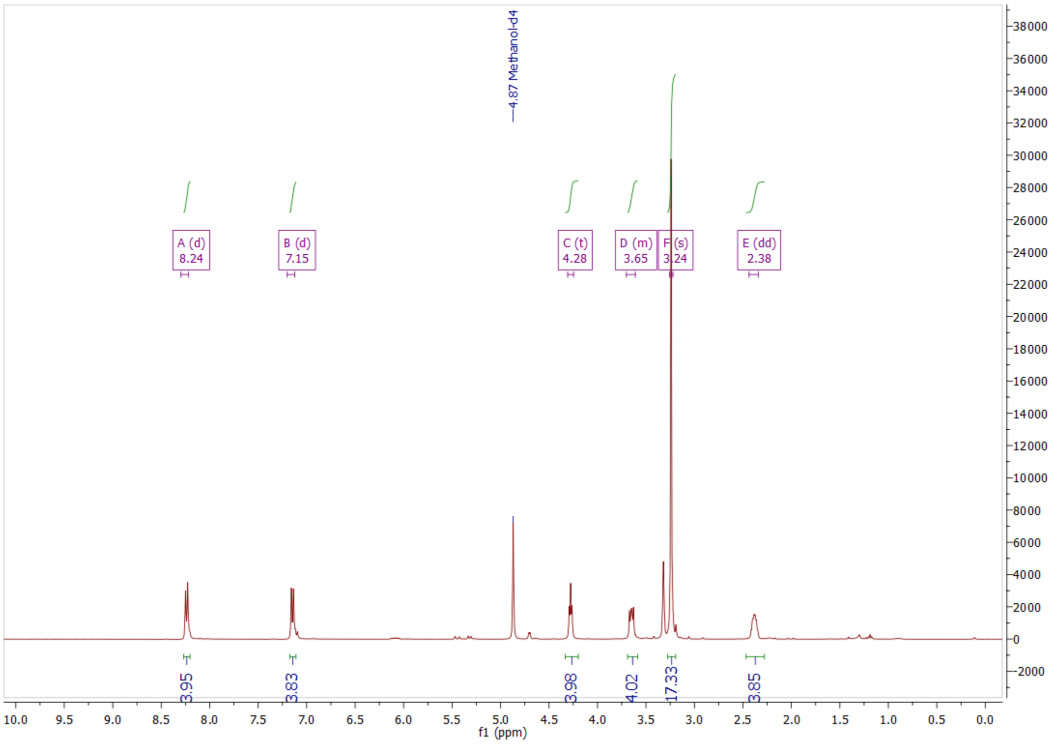

Figure S81. ^{1}H NMR spectrum of compound **15a** (400 MHz, Methanol-*d4*)

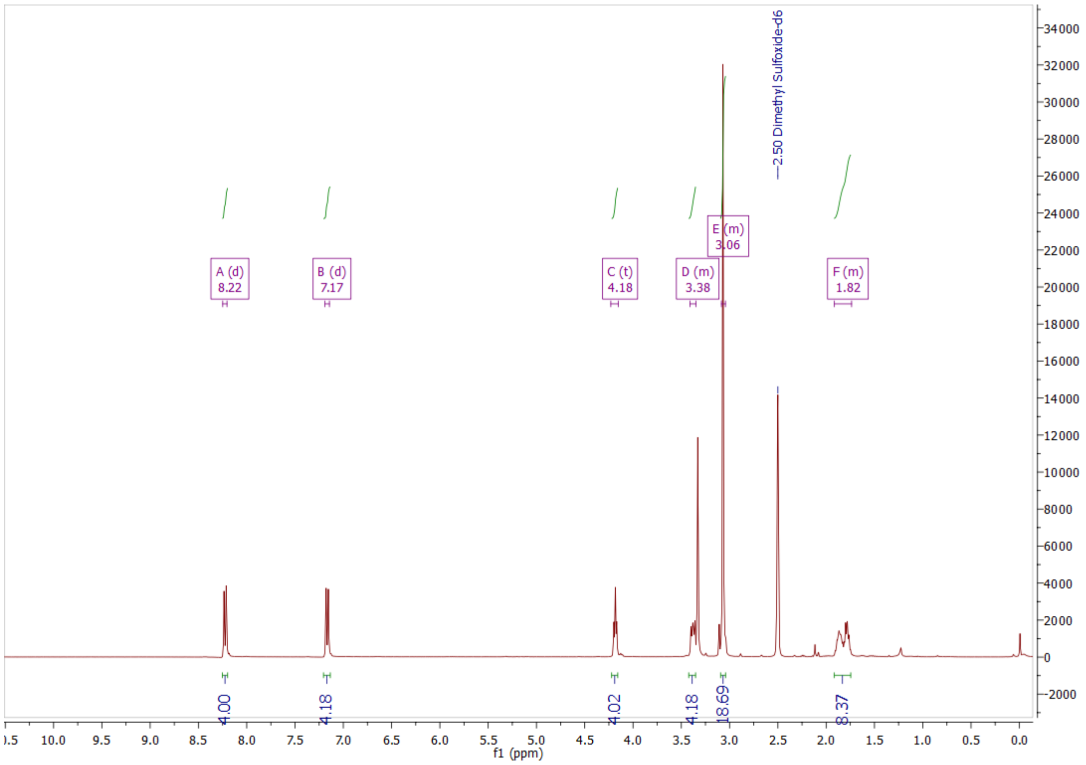

Figure S82. ^{1}H NMR spectrum of compound **15b** (400 MHz, DMSO-*d6*)

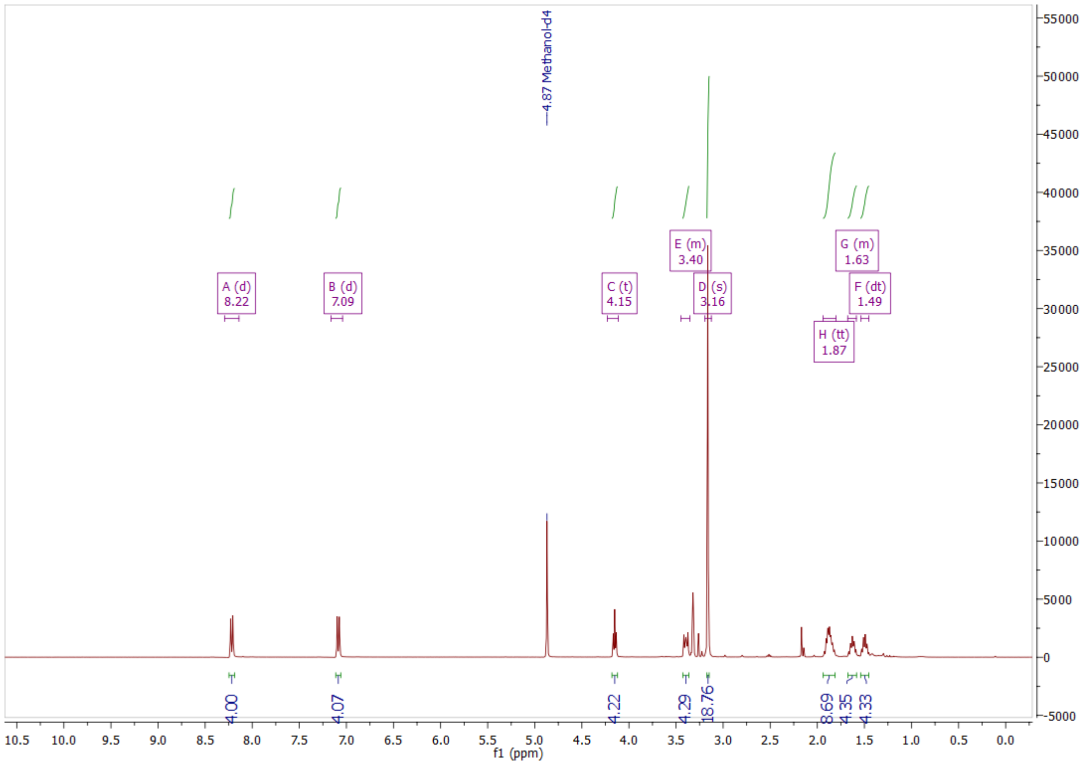

Figure S83. ^{1}H NMR spectrum of compound **15c** (400 MHz, Methanol-*d4*)

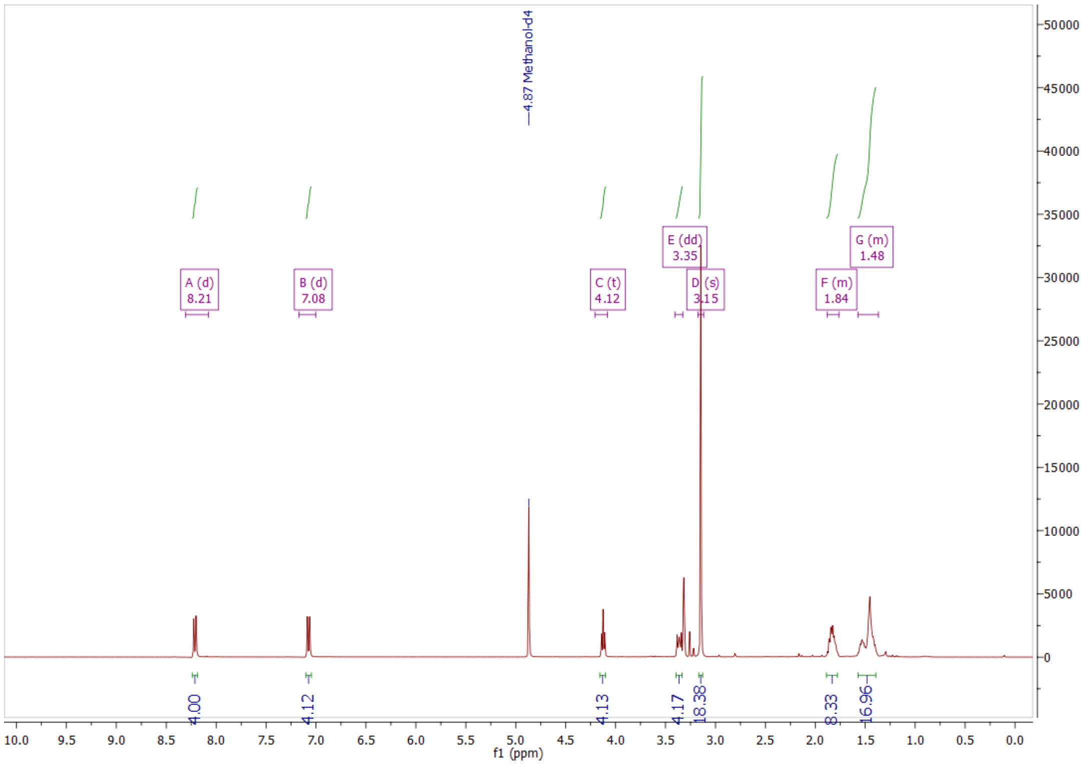

Figure S84. ^{1}H NMR spectrum of compound **15d** (400 MHz, Methanol-*d4*)

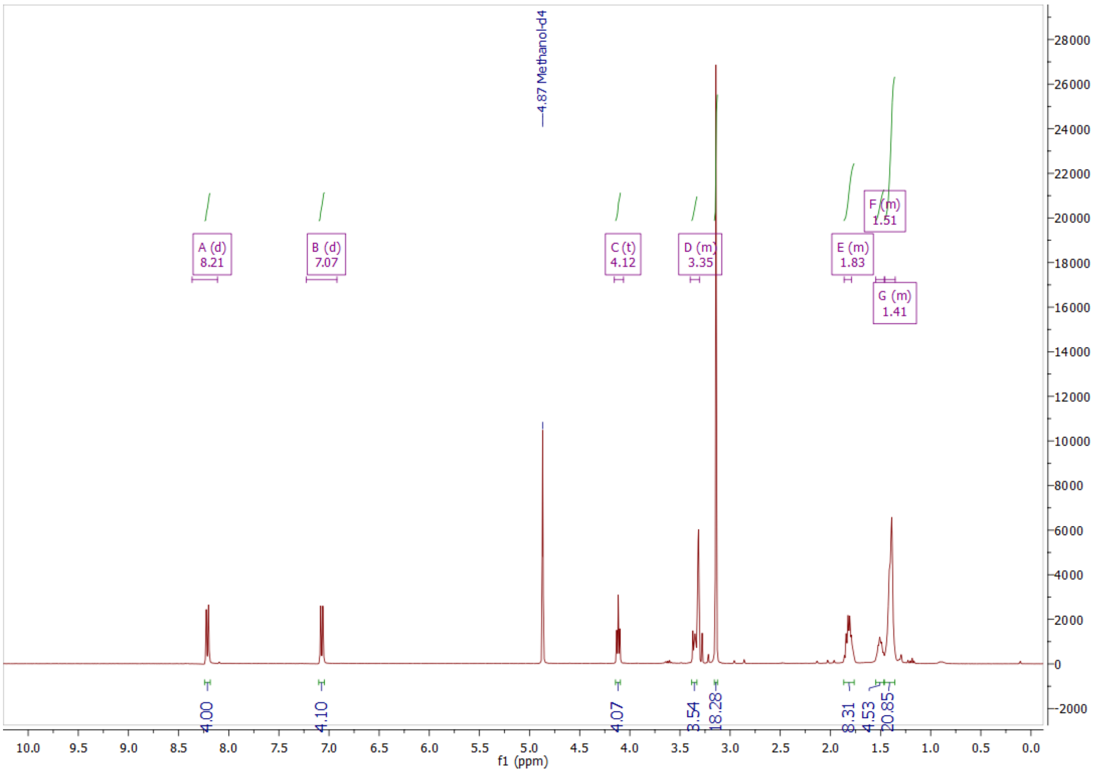

Figure S85. ^{1}H NMR spectrum of compound **15e** (400 MHz, Methanol-*d4*)

7. HPLC and HRMS spectra for compounds 10b and 10f

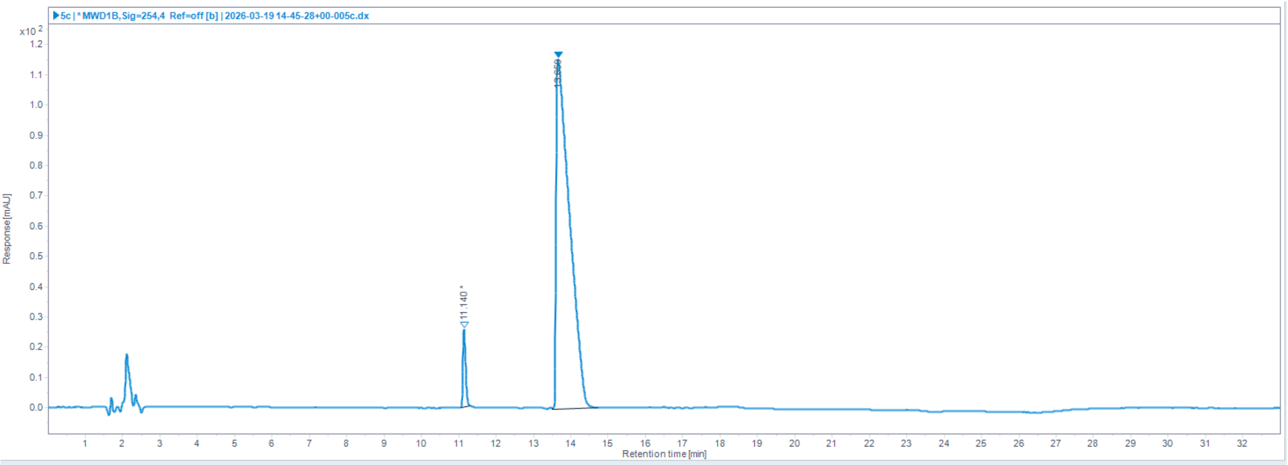

Injection Results

Peaks | Summary

#	Signal description	RT (min)	Area (mAU·s)	Area%	Height (mAU)	Height%	Amount	Start time (min)	End time (min)
1	* MWD1B,Sig=254,4 Ref=off [b]	11.140	126.862	4.230	25.586	18.13		11.062	11.304
2	* MWD1B,Sig=254,4 Ref=off [b]	13.659	2872.009	95.770	115.537	81.87		13.499	14.728

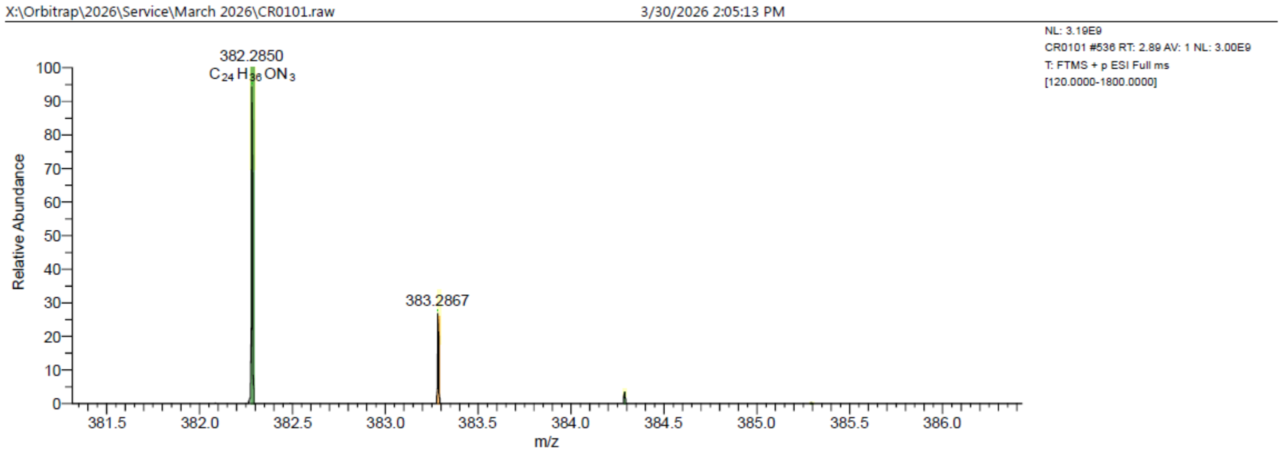

Figure S86. HPLC spectrum showing >95% purity (top) and HRMS spectrum (below) of compound **10b**

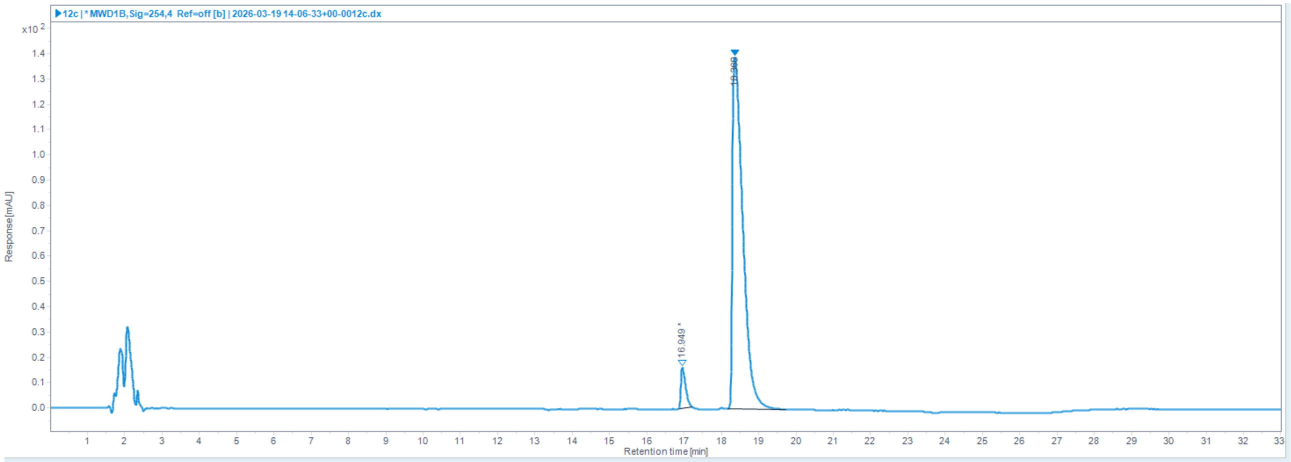

Injection Results

Peaks | Summary

#	Signal description	RT (min)	Area (mAU·s)	Area%	Height (mAU)	Height%	Amount	Start time (min)	End time (min)
1	* MWD1B,Sig=254,4 Ref=off [b]	16.949	139.336	4.888	16.118	10.39		16.870	17.206
2	* MWD1B,Sig=254,4 Ref=off [b]	18.360	2711.342	95.112	139.082	89.61		18.179	19.727

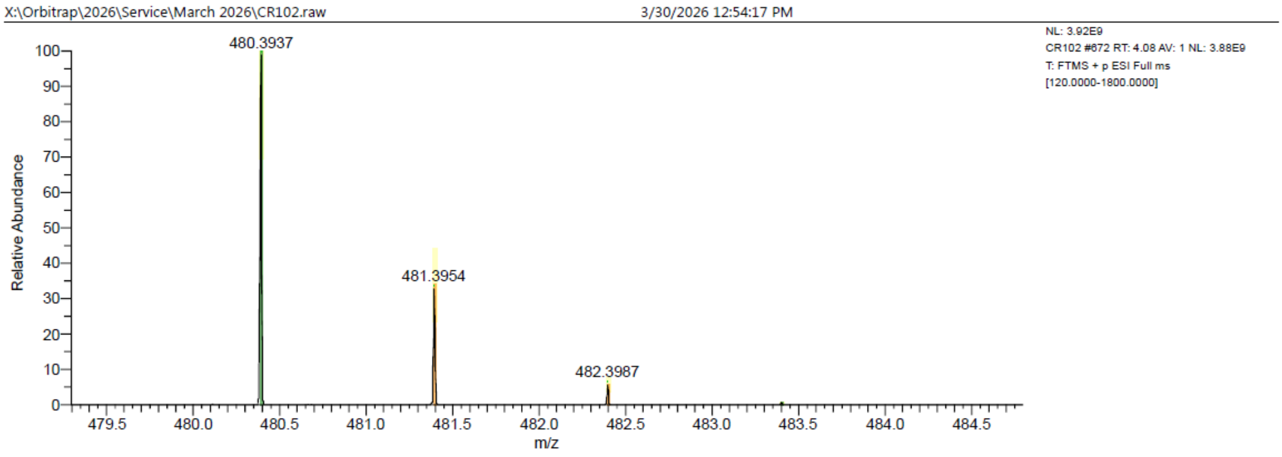

Figure S87. HPLC spectrum showing >95% purity (top) and HRMS spectrum (below) of compound **10f**
